# Generalised popularity-similarity optimisation model for growing hyperbolic networks beyond two dimensions

**DOI:** 10.1038/s41598-021-04379-1

**Published:** 2022-01-19

**Authors:** Bianka Kovács, Sámuel G. Balogh, Gergely Palla

**Affiliations:** 1 Department of Biological Physics, Eötvös Loránd University, Pázmány P. stny. 1/A, 1117 Budapest, Hungary; 2 MTA-ELTE Statistical and Biological Physics Research Group, Pázmány P. stny. 1/A, 1117 Budapest, Hungary; 3 Health Services Management Training Centre, Semmelweis University, 1125 Kútvölgyi út 2, Budapest, Hungary

**Keywords:** Complex networks, Statistical physics

## Abstract

Hyperbolic network models have gained considerable attention in recent years, mainly due to their capability of explaining many peculiar features of real-world networks. One of the most widely known models of this type is the popularity-similarity optimisation (PSO) model, working in the native disk representation of the two-dimensional hyperbolic space and generating networks with small-world property, scale-free degree distribution, high clustering and strong community structure at the same time. With the motivation of better understanding hyperbolic random graphs, we hereby introduce the *d*PSO model, a generalisation of the PSO model to any arbitrary integer dimension $d>2$. The analysis of the obtained networks shows that their major structural properties can be affected by the dimension of the underlying hyperbolic space in a non-trivial way. Our extended framework is not only interesting from a theoretical point of view but can also serve as a starting point for the generalisation of already existing two-dimensional hyperbolic embedding techniques.

## Introduction

Network theory has become an essential and ubiquitous tool for modelling various types of complex systems ranging from the level of interactions within cells to the level of the Internet, economic networks, and the society^1,2^. In the past decades, a vast number of related studies reported a few universal features that most of the real networks seem to have in common, such as sparsity^3^, small-world property^4,5^, inhomogeneous degree distribution^6,7^, high clustering coefficient^8^ or community structure^9–11^. Incorporating all, or at least some of these universal properties into a unified modelling framework is, however, a non-trivial issue and still presents a theoretical challenge of high relevance. Along this line, a variety of different network models have been proposed so far, including the celebrated Barabási–Albert (BA) model with preferential attachement^12^, the hidden variables formalism^13–17^ or models based on the mechanism of triadic closure, which has been specifically designed for explaining the high clustering of social networks^18,19^. Besides these examples, a further notable approach is given by hyperbolic network models that are capable of simultaneously explaining many observed network characteristics in a natural manner by assuming that nodes are embedded into a negatively curved hidden metric space^20–25^.

The random hyperbolic graph (RHG)^20^, for instance, is a static network model where nodes are placed at random on the hyperbolic disk of constant curvature $K=-\zeta ^2$, and the connection probability between any pair of nodes is a decreasing function of their hyperbolic distance. A mathematically equivalent model is given by the $\mathbb {S}^1$ model^26^, where the nodes are positioned on a circle and become connected according to a probability depending on the angular distance and a hidden variable drawn from a power-law distribution. By converting the hidden variables into radial coordinates we arrive to the hyperbolic $\mathbb {H}^2$ model^27^ that is equivalent to the RHG model; hence, the RHG is often also referred to as the $\mathbb {S}^1/\mathbb {H}^2$ model. (Actually, the most general form of the ${\mathbb {S}}^1/{\mathbb {H}}^2$ model described in Ref.^27^ is somewhat broader than the RHG model as it can accommodate any type of degree distributions.)

In contrast to the RHG, in the popularity-similarity optimisation (PSO) model^21^ the networks are not static but evolve over time via the continual appearance of new nodes on the hyperbolic plane. More precisely, new nodes are placed one by one in the native disk representation of the two-dimensional hyperbolic plane^20^ with logarithmically increasing radial coordinates and uniformly random angular coordinates. Once a new node appears, it establishes connections to the previous ones with a probability depending on the hyperbolic distance in a similar way as in the RHG model. The tendency to connect to hyperbolically close nodes can be interpreted as an optimisation of a trade-off between the popularity (arising from the node birth time and reflected by the radial coordinate) of a possible candidate and its similarity (the angular distance abstracting the distance in an attribute space) compared to the newly arriving node. In vague terms, the degree of the nodes is determined by the radial coordinate, and owing to an outward shift of the nodes (referred to as the popularity fading, controlled by a parameter $\beta$), the degree distribution takes the scaling form of $\mathcal {P}(k)\sim k^{-\gamma }$ with a tuneable decay exponent $\gamma =1+\frac{1}{\beta }$. By changing the sharpness of the cutoff in the connection probability as a function of the hyperbolic distance with another parameter *T* called temperature, the average clustering coefficient $\bar{c}$ of the resulting graphs can be adjusted as well. Although this model has been shown to be capable of generating networks that are small-world, highly clustered and scale-free at the same time, several other variants of the original PSO model have been suggested in order to explain further features of real-world graphs. Examples include the E-PSO model that inherently accounts for the creation of internal links, i.e. connections emerging between old nodes in the network^22^, or alternatively, the deletion of already existing links^28^. Or, the nonuniform popularity-similarity optimisation (nPSO) model^24,29^ that allows the generation of networks with an adjustable community structure by assuming a heterogeneous angular node distribution with multiple peaks is also worth mentioning. Nevertheless, it has also been revealed quite recently that both the RHG and the PSO models can generate networks that possess strong community structure despite lacking any explicitly built-in community generating mechanisms^25,30–34^.

In parallel with the developments of hyperbolic network models, another closely related field given by hyperbolic embedding techniques has also received great attention^22,27,28,35–38^. Briefly, this tackles the problem of inferring the most plausible coordinates for the network nodes based on the topology of a given network. One of the first methods pointing in this direction was HyperMap^22^, relying on a maximum likelihood estimation, where we assign hyperbolic coordinates to the nodes of the network by maximising the probability that the network was generated by the E-PSO model. Contrarily, in Refs.^36,37^ an embedding technique based on a nonlinear dimension reduction of the Laplacian matrix was introduced. Along similar lines, a whole set of embedding algorithms were studied in Ref.^38^, using different pre-weighted matrices encapsulating the network structure and multiple unsupervised dimension reduction techniques borrowed from machine learning. The rationale behind these approaches (usually coined as coalescent embeddings) is that when they are applied to hyperbolic networks, a common node aggregation pattern can be observed that is circularly or linearly ordered (angular coalescence) according to the original angular coordinates on the hyperbolic plane. An embedding algorithm that mixes the coalescent embedding with local angular optimisation based on likelihood maximisation was proposed in Ref.^28^. A further, very efficient embedding method is given by Mercator^27^, adopting the Laplacian eigenmaps approach with the coordinates optimised according to the RHG model.

Despite the excellent performance of the above embedding techniques, there is clearly a theoretical limitation behind most of them: they are defined on the hyperbolic disk, that is, in $d=2$ dimensions. Nevertheless, it has recently been revealed that higher-dimensional hyperbolic embeddings can outperform lower-dimensional ones in link prediction and in graph reconstruction with respect to mean average precision (MAP), for instance, in author collaboration networks^39^. Moreover, in Ref.^40^ it has also been shown that the presence of additional dimensions can lead to a clearer separation between the communities of a network, quantitatively confirmed by a higher value of the angular separation index (ASI). Furthermore, using higher number of dimensions can be advantageous also from the point of the behaviour of the optimisation procedures behind the embedding algorithms. I.e., in many cases the embedding is based on the maximisation of the likelihood that the network was generated according to a hyperbolic random graph model, and in 2 dimensions, this function can be extremely non-convex with respect to the node coordinates, making e.g. stochastic gradient descent methods inefficient^22^. However, raising the number of dimensions of the hyperbolic space can lift some of the local maxima, helping the optimisation methods in finding better solutions of the embedding problem. Uncovering the role of the number of dimensions in hyperbolic embeddings is, therefore, of great interest that simultaneously provides a strong motivation for investigating appropriate higher-dimensional hyperbolic network models as well.

The first hyperbolic network model that treats the number of dimensions as a model parameter by placing the nodes in a *d*-dimensional hyperbolic ball was considered in the Supplementary Information of Ref.^41^ in the context of its asymptotic equivalence to the causal sets in the *d*-dimensional de Sitter spacetime. Then, the RHG model has been extended to $d>2$ dimensions in Refs.^42,43^. Here, we introduce the *d*PSO model, a generalisation of the original two-dimensional popularity-similarity optimisation model to any arbitrary integer dimension of $d\ge 2$, which, we believe, provides a further substantial step towards a comprehensive theoretical characterisation of hyperbolic graphs.

Besides its theoretical relevance, our *d*PSO model opens up the possibility of systematically generalising already existing embedding techniques to higher dimensions, the necessity of which has explicitly been outlined e.g. for coalescent embeddings in Ref.^38^. Therein the authors claim that as a supplement to their findings, an additional interesting analysis could be to generate synthetic networks using for instance a three-dimensional PSO model and examine the accuracy of their methods by comparing the obtained embeddings to the original node arrangement. The present work contributes to this issue by thoroughly elaborating the PSO model for $d=3$ and higher dimensions. Since the higher number of dimensions of the underlying hyperbolic space allows a much richer characterisation of the nodes in general, here we conjecture that the suggested *d*-dimensional embeddings could provide further and deeper insights into the architecture of the hidden geometry behind the structure of complex networks.

In the present paper, we introduce the *d*PSO model as a natural generalisation of the well-known two-dimensional PSO model^21^ to hyperbolic spaces of dimension $d>2$. We show analytically that the degree distribution of *d*PSO networks can be written as $\mathcal {P}(k)\sim k^{-\gamma }$ in the large *k* regime, where the degree decay exponent $\gamma$ is directly related to the dimension *d* and the popularity fading parameter $\beta$ as $\gamma =1+\frac{1}{(d-1)\beta }$. Besides the scale-free behaviour, the networks generated by the *d*PSO model can exhibit a large average clustering coefficient $\bar{c}$ and a strong community structure for a relatively wide range of the parameter settings if the number of dimensions of the underlying hyperbolic space is not extremely high. According to our results, $\bar{c}$ is controlled by an interesting interplay between the dimension *d* and the temperature *T*, where for $T<\frac{1}{d-1}$ the average clustering coefficient is a decreasing function of *T*, whereas at temperatures near and above $T=\frac{1}{d-1}$, $\bar{c}$ becomes independent of *T*. A further noteworthy feature of the *d*PSO model is that in dimensions $d>2$ extremely skewed degree distributions with $\gamma <2$ become accessible, which can lead to networks displaying a number of exotic properties that are absent in scale-free networks with $2\le \gamma$. The rich variety of networks that can be obtained in our proposed framework together with the capability of reproducing the fundamental properties of real networks in a natural way make the *d*PSO model a very promising candidate upon which higher-dimensional hyperbolic embedding techniques may be developed in the future.

## Methods

The original popularity-similarity optimisation model places the network nodes in the native representation of the hyperbolic plane during the network generation. First, in the following section we describe the native representation of the *d*-dimensional hyperbolic space and the corresponding formula of the hyperbolic distance. Next, the network generation algorithm of the *d*PSO model is introduced by extending the PSO model to the hyperbolic space of any integer dimension $d\ge 2$.

### Native representation of the hyperbolic space

The *d*-dimensional hyperbolic space of constant curvature $K<0$ is represented in the so-called native representation^20^ by a *d*-dimensional ball of infinite radius in the Euclidean space (for which $K=0$). In this representation the Euclidean angles between hyperbolic lines are equal to their hyperbolic values, and the radial coordinate *r* of a point (defined as its Euclidean distance from the centre of the ball) is equal to its hyperbolic distance from the ball centre. The hyperbolic distance between two points is measured along their connecting hyperbolic line, which is either an arc going through the given points and intersecting the ball’s boundary perpendicularly or – if the ball centre falls on the Euclidean line connecting the two points in question – the corresponding diameter of the ball. The hyperbolic distance *x* between two points given by the Cartesian coordinate vectors $\underline{u}=(u_1,u_2,...,u_d)$ and $\underline{v}=(v_1,v_2,...,v_d)$ of norms $\Vert \underline{u}\Vert =\sqrt{\sum _{q=1}^d u_{q}^2}\equiv r_u$ and $\Vert \underline{v}\Vert =\sqrt{\sum _{q=1}^d v_{q}^2}\equiv r_v$ fulfills the hyperbolic law of cosines written as

1 $$\begin{aligned} \mathrm {cosh}(\zeta x)=\mathrm {cosh}(\zeta r_u)\,\mathrm {cosh}(\zeta r_v)-\mathrm {sinh}(\zeta r_u)\,\mathrm {sinh}(\zeta r_v)\,\mathrm {cos}(\theta _{u,v}), \end{aligned}$$

where $\zeta =\sqrt{-K}$, and $\theta _{u,v}=\mathrm {arccos}(\frac{\underline{u}\cdot \underline{v}}{\Vert \underline{u}\Vert \,\Vert \underline{v}\Vert })=\mathrm {arccos}(\frac{\sum _{q=1}^d u_{q} v_{q}}{r_u r_v})$ is the angle between the examined points. Note that in the case of $r_u=0$ simply $x=r_v$, and if $r_v=0$ then $x=r_u$. According to Ref.^20^, for sufficiently large $\zeta r_u$ and $\zeta r_v$ with an angular distance $\theta _{u,v}$ larger than $2\cdot \sqrt{e^{-2\zeta r_u}+e^{-2\zeta r_v}}$ but small enough to use the approximation $\sin (\theta _{u,v}/2)\approx \theta _{u,v}/2$, the hyperbolic distance can be approximated as

2 $$\begin{aligned} x {\,\,\approx r_u+r_v+\frac{2}{\zeta }\cdot \ln \left( \sin {\left( \frac{\theta _{u,v}}{2}\right) }\right) }\approx r_u+r_v+\frac{2}{\zeta }\cdot \ln \left( \frac{\theta _{u,v}}{2}\right) . \end{aligned}$$

### Description of the extended PSO model

The PSO model^21^ generates networks that have a scale-free degree distribution characterised by a degree decay exponent $\gamma$ that is determined by the radial arrangement of the network nodes. In terms of these, there are two natural possibilities for the extension of the two-dimensional PSO model to any integer number of dimensions $d\ge 2$ that both gives back the original PSO model at $d=2$: one can either make $\gamma$ independent of the number of dimensions by introducing a *d*-dependent multiplier in the radial coordinates of the low-temperature regime that yields highly clustered networks, or not change this coordinate formula compared to the two-dimensional case and make the degree decay exponent $\gamma$ dependent on the value of *d*. In the present study, we chose the latter option since it offers the opportunity to expand the range of the achievable $\gamma$ values below 2 by increasing the number of dimensions. This choice is established in more detail in Sect. S1.4 of the Supplementary Information, where we also introduce the *f*PSO model that provides the possibility to decrease the degree decay exponent $\gamma$ below 2 even in the two-dimensional case by treating the multiplying factor in the formula of the initial radial coordinates as a new model parameter.

In the *d*PSO model, the network nodes appear one by one in the above described native representation of the *d*-dimensional hyperbolic space and connect to previously appeared nodes with probabilities depending on the hyperbolic distances. The parameters of the model can be listed as follows:

- The curvature $K\in \mathbb {R}^-$ of the hyperbolic space, controlled by $\zeta =\sqrt{-K}>0$. Changing the value of $\zeta$ corresponds to a simple rescaling of the hyperbolic distances. The usual custom is to set the value of $\zeta$ to 1 (i.e. *K* to $-1$).
- The dimension $2\le d\in \mathbb {Z}^+$ of the hyperbolic space.
- The final number of nodes $N\in \mathbb {Z}^+$ in the network.
- The number of connections $m\in \mathbb {Z}^+$ established by each node after the *m*th one at its appearance. The average degree of the network is approximately $\bar{k}=2\cdot m$.
- The popularity fading parameter $\beta \in (0,1]$, controlling the outward drift of the nodes in the native ball. The exponent $\gamma$ of the power-law decaying tail of the degree distribution is related to the popularity fading parameter as
  3 $$\begin{aligned} \gamma =1+\frac{1}{(d-1)\cdot \beta }. \end{aligned}$$
  According to this relation between $\gamma$, $\beta$ and *d*, in the case of different dimensions different popularity fading parameters are needed to obtain the same degree decay exponent $\gamma$. Note that as the dimension *d* increases, the achievable smallest degree decay exponent ($\gamma _{\mathrm {min}}=1+1/(d-1)$, yielded by $\beta =1$) decreases, meaning that in higher dimensions the attainable highest degree is larger than in hyperbolic spaces of smaller dimensions. (The details of the derivation of Eq. (3) are given in Sect. Degree distribution and in Sect. S1 of the Supplementary Information.)
- The temperature $0\le T$, $T\ne \frac{1}{d-1}$, controlling the average clustering coefficient $\bar{c}$ of the network. As the temperature increases from 0, the average clustering coefficient decreases, and settles to a more or less constant value at $T=\frac{1}{d-1}$. For $\beta \le \frac{1}{d-1}$ (i.e., for $2\le \gamma$), the clustering is asymptotically zero for any $\frac{1}{d-1}<T$, while for larger popularity fading parameters (i.e., for $\gamma <2$) the lowest possible $\bar{c}$ is an increasing function of $\beta$.

During the random graph generation process, initially the network is empty, and at each time step $j=1,2,...,N$ a new node joins the network as follows:

1. The new node *j* appears at radial distance $r_{jj}$ from the origin with a position chosen uniformly at random on the surface of the corresponding *d*-dimensional ball by sampling independently *d* number of Cartesian coordinates from the standard normal distribution and then normalising the obtained position vector (i.e., dividing all the sampled coordinates $u_{q},\,q\in [1,d]$ by $\sqrt{\sum _{q=1}^d u_{q}^2}$ and then multiplying all of them by $r_{jj}$). The radial coordinate $r_{jj}$ is calculated as
  - $$r_{jj}=\frac{2}{\zeta }\ln {j}$$ if $$T<\frac{1}{d-1}$$, and
  - $$r_{jj}=\frac{2T(d-1)}{\zeta }\ln {j}$$ if $$\frac{1}{d-1}<T$$,
  where the change in the multiplying factor at $T=1/(d-1)$ was introduced to ensure that the formula of the degree decay exponent $\gamma$ remains the same (given by Eq. (3)) for all temperature settings. (Note that a similar adjustment of $r_{jj}$ was already introduced at $T=1$ in the original PSO model of $d=2$^21^.)
2. The radial coordinate of each previously (at time $i<j$) appeared node *i* is increased according to the formula $r_{ij}=\beta r_{ii}+(1-\beta )r_{jj}$ in order to simulate popularity fading.
3. The new node *j* establishes connections with *m* number of previously appeared nodes. Only single links are permitted. If the number of previously appeared nodes is not larger than *m*, then node *j* connects to all of them. Otherwise (i.e., for $m+1<j$),
  - node *j* connects to the *m* hyperbolically closest nodes if $T=0$, and
  - at temperatures $0<T$, any previous node $i=1,2,...,j-1$ gets connected to node *j* with probability
    4 $$\begin{aligned} p(x_{ij})=\frac{1}{1+e^{\frac{\zeta }{2T}(x_{ij}-R_j)}}, \end{aligned}$$
    where the hyperbolic distance $x_{ij}$ between the node pair $i-j$ can be calculated based on Eq. (1) and the so-called cutoff distance $R_j$ can be obtained by solving the equation $m=\bar{k}_j$, where the expected number $\bar{k}_j$ of the realised connections of the new node *j* at its arrival time *j* can be written as
    5 $$\begin{aligned} \bar{k}_j=\eta (d)\cdot \int _1^j \int _0^{\pi } \frac{\sin ^{d-2}{\theta _{ij}}}{1+\left( e^{\frac{\zeta }{2}\cdot (r_{ij}+r_{jj}-R_j)}\cdot \sin {\left( \frac{\theta _{ij}}{2}\right) }\right) ^{\frac{1}{T}}} \,\mathrm {d}\theta _{ij}\,\mathrm {d}i \end{aligned}$$
    using
    6 $$\begin{aligned} \eta (d)=\left\{ \begin{array}{ll} \frac{\left( \frac{d}{2}-1\right) !\cdot \frac{d-2}{2}!\cdot 2^{d-2}}{(d-2)!\cdot \pi } &{} \text{ if }\; d \text{ is } \text{ even, } \\ \frac{(d-1)!}{\left( \frac{d-1}{2}-1\right) !\cdot \frac{d-1}{2}!\cdot 2^{d-1}} &{} \text{ if }\; d \text{ is } \text{ odd. } \end{array} \right. \end{aligned}$$
    As detailed in Sect. S2 of the Supplementary Information, at $\beta \ne 1/(d-1)$ the cutoff distance can be approximated as
    7 $$\begin{aligned} R_j\approx \left\{ \begin{array}{ll} \frac{2}{\zeta \cdot (d-1)}\cdot \ln \left( \frac{m\cdot \sin ((d-1)\cdot T\cdot \pi )\cdot (1-(d-1)\cdot \beta )}{\eta (d)\cdot \pi \cdot 2^{d-1}\cdot T\cdot \left( j^{3-2\cdot d}- j^{(d-1)\cdot (\beta -2)}\right) }\right) &{} \text{ if }\; 0<T<\frac{1}{d-1}, \\ \\ \frac{2\cdot T}{\zeta }\cdot \ln \left( \frac{m\cdot (1-(d-1)\cdot \beta )}{\eta (d)\cdot \int _0^{\pi }\sin ^{d-2}(\phi )\cdot \sin ^{-1/T}({\phi /2})\,\,\mathrm {d}\phi \,\cdot \left( j^{3-2\cdot d}- j^{(d-1)\cdot (\beta -2)}\right) }\right) &{} \text{ if }\; \frac{1}{d-1}<T, \end{array} \right. \end{aligned}$$
    whereas at $\beta =1/(d-1)$ the formula
    8 $$\begin{aligned} R_j\approx \left\{ \begin{array}{ll} \frac{2}{\zeta \cdot (d-1)}\cdot \ln \left( \frac{m\cdot \sin ((d-1)\cdot T\cdot \pi )\cdot j^{2\cdot d-3}}{\eta (d)\cdot \pi \cdot 2^{d-1}\cdot T\cdot \ln {j}}\right) &{} \text{ if }\; 0<T<\frac{1}{d-1}, \\ \\ \frac{2\cdot T}{\zeta }\cdot \ln \left( \frac{m\cdot j^{2\cdot d-3}}{\eta (d)\cdot \int _0^{\pi }\sin ^{d-2}(\phi )\cdot \sin ^{-1/T}(\phi /2)\,\,\mathrm {d}\phi \,\cdot \ln {j}}\right) &{} \text{ if }\; \frac{1}{d-1}<T. \end{array} \right. \end{aligned}$$
    can be used, where $\int _0^{\pi }\sin ^{d-2}(\phi )\cdot \sin ^{-1/T}({\phi /2})\,\,\mathrm {d}\phi$ can be calculated numerically.

## Results

**Figure 1:**
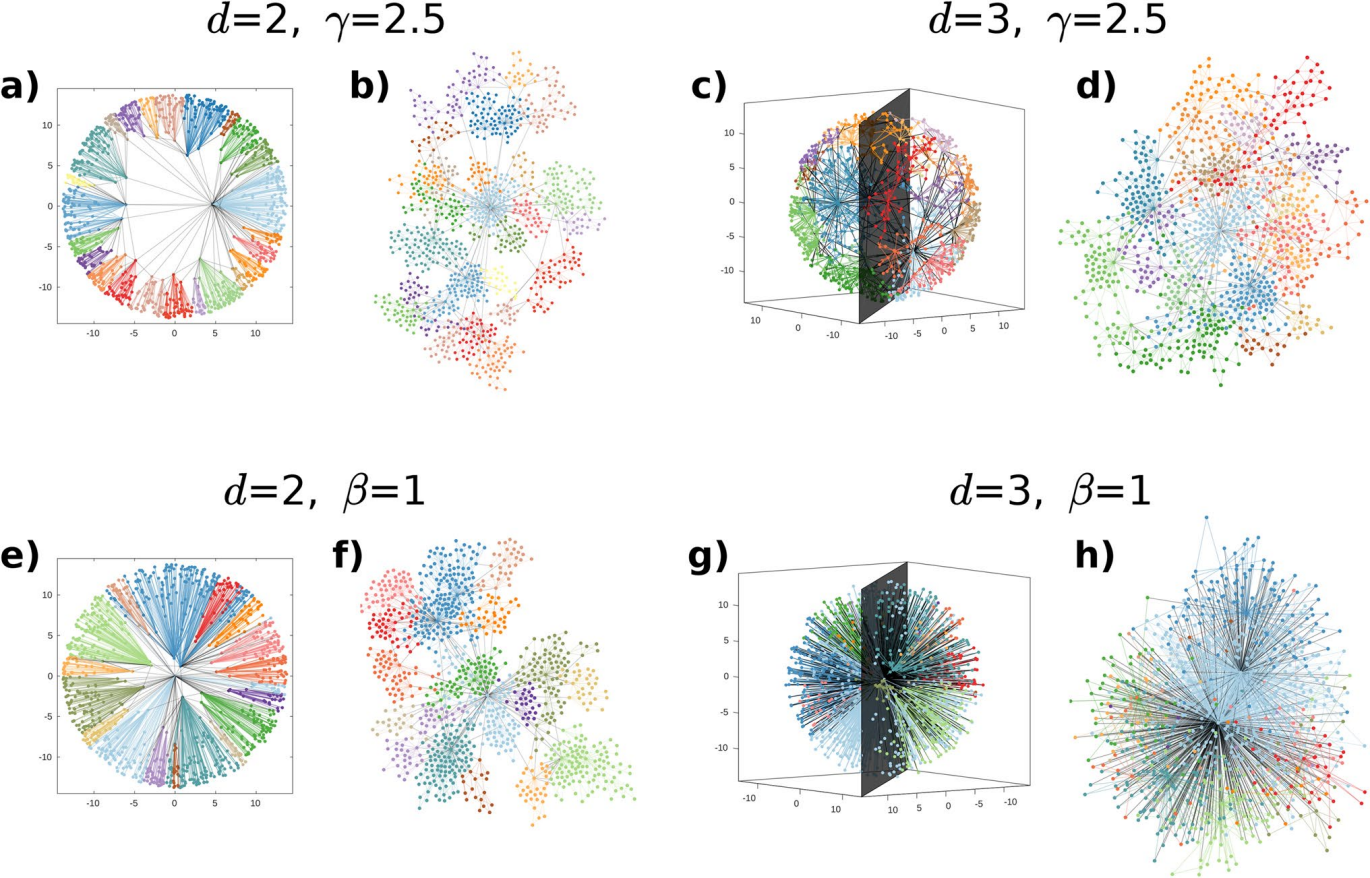
Layouts of networks generated by the *d*PSO model in 2- and 3-dimensional hyperbolic spaces of curvature $K=-1$. The colouring of the nodes and the links indicates communities found by the Louvain algorithm. In panels **a**, **c**, **e**, **g** we display the network in the native *d*-dimensional hyperbolic ball, whereas panels **b**,** d**, **f**, **h** show the standard Euclidean layout of the same graph for comparison. In the top row, we present networks of the same degree decay exponent $\gamma =2.5$, generated on the 2-dimensional hyperbolic plane, setting the popularity fading parameter $\beta$ to 2/3 (panels **a**,** b**), and in the 3-dimensional hyperbolic space, setting the popularity fading parameter $\beta$ to 1/3 (panels **c**,** d**). In the bottom row, we show networks of the same popularity fading parameter $\beta =1$, corresponding to the smallest degree decay exponent achievable in a given dimension, namely $\gamma =2.0$ in the 2-dimensional case (panels **e**,** f**) and $\gamma =1.5$ in the 3-dimensional case (panels **g**,** h**). For each network, we set the number of nodes *N* to 1000, the expected average degree 2*m* to 4 and the temperature *T* to 0. The average clustering coefficient $\bar{c}$ of the displayed networks and the modularity *Q* for their displayed partitions are the following: $\bar{c}=0.729$ and $Q=0.882$ for panels **a**, **b**; $\bar{c}=0.644$ and $Q=0.845$ for panels **c**,** d**; $\bar{c}=0.788$ and $Q=0.829$ for panels **e**, **f**; while $\bar{c}=0.964$ and $Q=0.354$ for panels **g**,** h**.

We generated networks with the above-described *d*PSO model using numerous parameter settings. As an illustration, in Fig. 1 we show layouts of *d*PSO networks of size $N=1000$ both in the native representation of the *d*-dimensional hyperbolic space and according to a standard force-directed layout algorithm in the Euclidean plane. We analyzed the major structural properties of *d*PSO networks from various points of view. The results along with their detailed explanations are presented in the following subsections, focusing on a few fundamental network characteristics such as the degree distribution (Sect. Degree distribution), the average clustering coefficient (Sect. Clustering coefficient) and the community structure (Sect. Finding and evaluating communities). Further plots regarding simulation results are shown in Sect. S3 of the Supplementary Information. Moreover, in Sect. S4 of the Supplementary Information we also study a three-dimensional extension of the nonuniform popularity-similarity optimisation model (nPSO)^24,29^ that samples the angular coordinates of the network nodes from a multimodal distribution to allow control over the number and the size of the communities.

### Degree distribution

Our analytical calculations show that when generating networks according to the *d*PSO model presented in the Methods section, we obtain degree distributions that follow a scaling form of

9 $$\begin{aligned} \mathcal {P}(k)\sim k^{-\gamma }, \end{aligned}$$

where the degree decay exponent $\gamma$, given by Eq. (3), depends on the popularity fading parameter $\beta$ and also the number of dimensions *d*, but is independent of the temperature *T*. This is a direct consequence of the fact that the probability for node *i* (appearing at time *i*) to attract a link from node *j* (appearing at time $j > i$) in the above model can be written as

10 $$\begin{aligned} \Pi _{d\text {PSO}}(i,j)=m\cdot \frac{i^{-(d-1)\beta }}{\int ^{j}_{1}\ell ^{-(d-1)\beta }\mathrm {d}\ell }, \end{aligned}$$

with *m* denoting the number of connections established by node *j* at its appearance. (The derivation of the above formula is given in Sect. S1 of the Supplementary Information.) Indeed, by following the idea in Ref.^21^, it can be shown that Eq. (10) is equivalent to imposing an extended preferential attachment rule (EPA) that defines the connection probability between an existing node *i* with a degree $k_i(j)$ and the newly appearing node *j* as

11 $$\begin{aligned} \Pi _{\text {EPA}}[k_i(j)]=m\frac{k_i(j)-m+A}{(m+A)j}, \end{aligned}$$

where $A=(\gamma -2)m$ is a parameter called initial attractiveness. According to Ref.^44^, in networks generated by this EPA rule, the expected degree of node *i* becomes

12 $$\begin{aligned} \overline{k_i(j)}=m+A\left[ \left( \frac{i}{j}\right) ^{-\alpha }-1\right] \end{aligned}$$

at time *j*, and the degree distribution develops into a scale-free form of $\mathcal {P}(k)\sim k^{-\gamma }$, where the exponents $\alpha$ and $\gamma$ are connected by $\alpha (\gamma )=\frac{1}{\gamma -1}$. We can relate Eqs. (11) and (12) to Eq. (10) by identifying $\alpha$ as $\alpha =(d-1)\beta$, verifying that

13 $$\begin{aligned} \Pi _{d\text {PSO}}(i,j)=\Pi _{\text {EPA}}[\overline{k_i(j)}]. \end{aligned}$$

**Figure 2:**
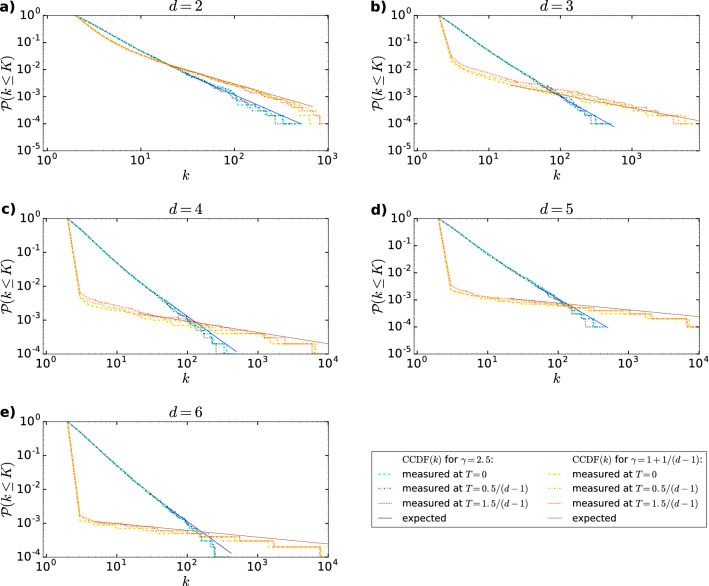
Degree distribution of networks generated by the *d*PSO model at different parameter settings. Panels **a**–**e** correspond to different dimensions *d* from 2 to 6. We display in all the panels the complementary cumulative distribution function (CCDF) of the node degrees for networks of two different degree decay exponents: $\gamma =2.5$ (blue) and $\gamma =1+1/(d-1)$ (orange). In both cases, we generated a network using the temperature setting $T=0$ (dashed lines), $T=0.5/(d-1)$ (dash-dotted lines) or $T=1.5/(d-1)$ (dotted lines). The curvature of the hyperbolic space, the number of nodes and the half of the expected average degree were the same for all networks, namely $K=-\zeta ^2=-1$, $N=10,000$ and $m=2$. All the indicated simulation results match well the curve $\mathcal {P}(k\le K) \sim k^{-(\gamma -1)}$ (shown by solid lines) that was expected based on the analytical calculations.

Finally, since $\gamma$ can be expressed from $\alpha (\gamma )$ as $\gamma =1+\frac{1}{\alpha }$, we arrive at the formula in Eq. (3) for the decay exponent of the degree distribution in Eq. (9). Thus, networks that grow according to the rules outlined in the Methods section inherit the scale-free property from the original PSO model, albeit using the same $\beta$ parameter leads to a smaller $\gamma$ exponent for greater values of *d*. Note that by setting *d* to 2, one can easily recover the results of the original PSO model in Ref.^21^, where the degree decay exponent has been found to be $\gamma =1+\frac{1}{\beta }$. The analytical results are in perfect agreement with the numerical simulations, as indicated by Fig. 2, where we display the complementary cumulative distribution function (CCDF) of the node degrees for several networks obtained from the *d*PSO model with different combinations of the *d* and the $\beta$ parameters at different values of the temperature *T*.

### Clustering coefficient

**Figure 3:**
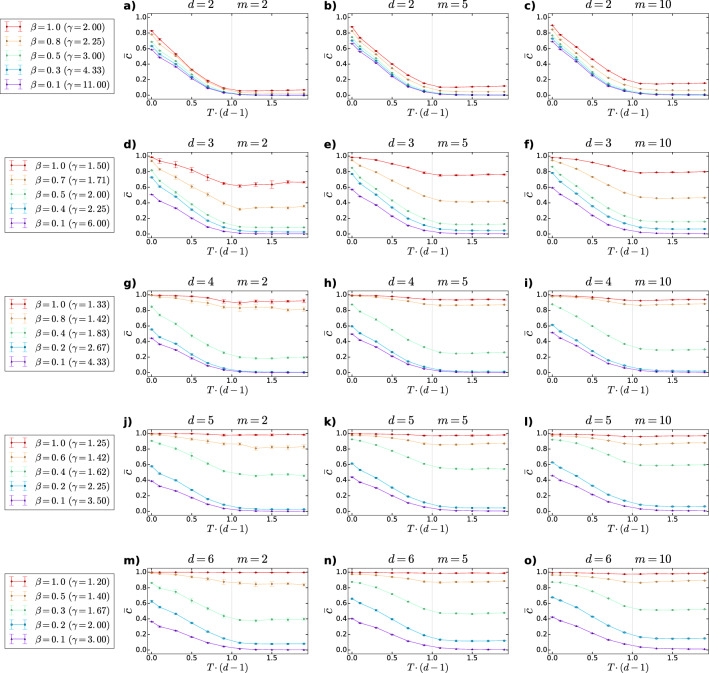
Average clustering coefficient $\bar{c}$ of *d*PSO networks as a function of the rescaled temperature $T\cdot (d-1)$. Each row of panels (i.e., panels **a**–**c**, **d**–**f**, **g**–**i**, **j**–**l** and **m**–**o**) was created using a given dimension *d*, and each column of subplots presents the results obtained with a given value of the expected average degree 2*m*, as written in the panel titles. The different curves of each panel correspond to different values of the popularity fading parameter $\beta$ (yielding different degree decay exponents $\gamma$), listed for each dimension in the leftmost panel of the corresponding row. The displayed data points were obtained by averaging over 5 *d*PSO networks generated independently with a given set of model parameters, setting the number of nodes to 10, 000 and the curvature of the hyperbolic space to $-1$ in each case. The error bars show the standard deviations measured among the 5 networks. The grey vertical lines indicate the critical point $T_{\mathrm {c}}=1/(d-1)$.

Based on simulations, we have found that the average clustering coefficient $\bar{c}$ of *d*PSO networks displays an unusually rich behaviour depending on the specific choices of the model parameters. First, in Fig. 3 we show the measured $\bar{c}$ as a function of the rescaled temperature $T \cdot (d-1)$ for different settings of the further model parameters. A rather simple observation regarding this figure is that $\bar{c}$ is an increasing function of *m*. This is reasonable in the light of the role of this model parameter outlined in the Methods section: *m* is related to the expected average degree of the *d*PSO networks as $\bar{k}=2\cdot m$, meaning that higher values of *m* correspond to higher average degrees.

Besides, according to Fig. 3, the average clustering coefficient is always maximal at $T=0$, i.e. when newly appearing nodes connect only to the hyperbolically closest existing nodes. If, however, the temperature increases, the cutoff in the connection probability becomes milder. This implies that rather distant nodes also become likely to be connected and consequently, the value of $\bar{c}$ decreases. Nevertheless, above a certain point $T_{\mathrm {c}}=\frac{1}{d-1}$, hereinafter referred to as the critical temperature, the average clustering coefficient remains more or less constant. This can be attributed to the fact that at the critical temperature we change the formula of the radial coordinates from $r_{jj}=\frac{2}{\zeta }\ln {j}$ to $r_{jj}=\frac{2T(d-1)}{\zeta }\ln {j}$, meaning that the radial coordinates become an increasing function of the temperature above $T_{\mathrm {c}}$. According to the approximating formula given by Eq. (2), the larger radial coordinates obviously yield larger hyperbolic distances between all node pairs. Therefore, despite the continuous slowing down of the decay in the connection probability as a function of the hyperbolic distance, above $T_{\mathrm {c}}=\frac{1}{d-1}$ the increase in the temperature does not increment further the number of nodes that are reachable for a newly coming node, and thus, the average clustering coefficient becomes settled to a constant value.

Finally, it can be clearly seen in Fig. 3 that the average clustering coefficient is an increasing function of the popularity fading parameter $\beta$. This is especially striking in the higher-dimensional cases, where the range of the degree decay exponents that are achievable extends to lower values. When $\gamma =1+\frac{1}{(d-1)\beta }$ is decreased, the degree distribution of the emerging network decays more slowly and, consequently, the largest occurring degrees increase. This is realised by the increase in the preference of the newly coming nodes for connecting to the early-appeared ones , overshadowing the attractiveness of the angular neighbours located at larger radii.

Now let us concentrate on the subgraph of nodes that appeared before a given time point. Since each node has to create *m* links at its appearance, the ratio between the connected and the non-connected node pairs in this subgraph increases towards earlier times (i.e., as the number of nodes in the subgraph decreases). Therefore, if the degree decay exponent $\gamma$ of the network is decreased, the set of the primarily attractive inner nodes tightens and the new nodes tend to connect into a more densely connected group of nodes, which increases the number of triangles and, accordingly, the average clustering coefficient. It is important to note that at extremely small values of $\gamma$, the high density of the connections among the most popular inner nodes moderates the effect of the temperature increase on $\bar{c}$ by limiting the probability to connect to nodes that are not connected to each other. Due to this, the networks of small enough degree decay exponents are highly clustered not only at small temperatures, but in the high-temperature regime as well.

**Figure 4:**
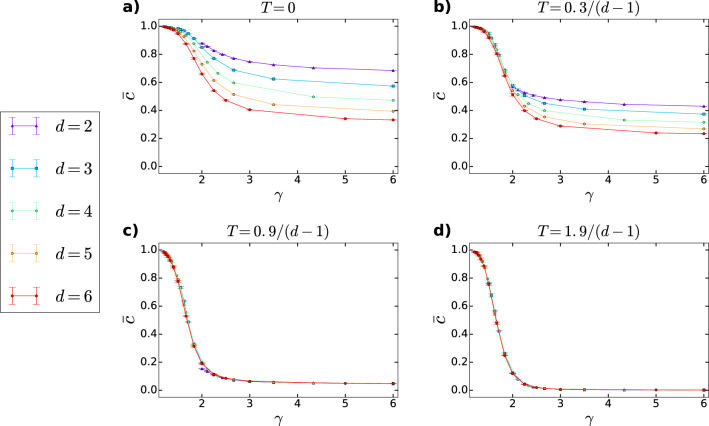
Average clustering coefficient $\bar{c}$ measured in *d*-dimensional PSO networks as a function of the degree decay exponent $\gamma$ at different values of the temperature *T* and the dimension *d*. We plotted the average clustering coefficient averaged over 5 networks for each parameter setting, with the error bars indicating the standard deviations among the 5 networks. The size of the networks was $N=10,000$, the expected average degree was $\bar{k}=2m=10$, and each network was generated in a hyperbolic space of curvature $K=-1$. The curves of different colours correspond to different values of the dimension *d* of the hyperbolic space, listed in the legend. The popularity fading parameter was always set to $\beta =\frac{1}{(d-1)\cdot (\gamma -1)}$. The different panels **a**–**d** were created using different values of the temperature *T*, specified in the panel title.

To provide further insight into how the model parameters affect the triangle formation in *d*PSO networks, in Fig. 4 we show the average clustering coefficient as a function of the degree decay exponent $\gamma$ at different settings of the temperature *T* and the dimension *d*. This figure again proves that the critical temperature $T_{\mathrm {c}}=\frac{1}{d-1}$ separates two distinct regimes, where $\bar{c}$ shows a fundamentally different nature.

Below the critical temperature $T_{\mathrm {c}}=\frac{1}{d-1}$ (Fig. 4a,b), the value of $\bar{c}$ measured at a given degree decay exponent $\gamma$ is affected by the number of dimensions of the underlying hyperbolic space: *d*PSO networks created in higher-dimensional spaces using smaller popularity fading parameters display smaller values of $\bar{c}$ compared to networks that have the same degree decay exponent $\gamma$ but were generated in lower-dimensional hyperbolic spaces with higher popularity fading parameters. This can be explained roughly by considering that at not too high temperatures, the newly appearing nodes tend to connect to already existing nodes that are relatively similar to them, where high similarity means small angular distance^21^. If the number of dimensions or, equivalently, the number of independent angular coordinates characterising the node attributes is increased, the number of possible coordinate combinations that define the set of positions of similar attributes for a given new node also increases. Therefore, any two nodes that can be considered to be similar from a third node’s point of view are less and less likely to have angular coordinates that are relatively close to each other as well with the increase in the number of dimensions. Consequently, the selected nodes to which the new node connects tend to share fewer links in higher dimensions; thus, the number of triangles in the emerging network is reduced.

As we approach the critical point from below, the role of the number of dimensions in local triangle formation gradually weakens. Near to and above $T = T_{\mathrm {c}}$ (Fig. 4c,d), where connections between rather distant nodes are likely to occur too, the value of $\bar{c}$ is independent of the individual values of the *d* and the $\beta$ parameters that produce the same degree decay exponent $\gamma$. Here, it seems that the randomising influence of the high temperature on link formation is so strong that the similar effect of the large number of dimensions is negligible compared to it.

Finally, let us consider the case where both the degree decay exponent $\gamma$ and the average clustering coefficient $\bar{c}$ are fixed by some external condition (e.g., when modelling real networks), and the goal is to provide parameter settings using which the *d*PSO model is capable of reproducing these fixed values. As described above, $\gamma$ is controlled by $(d-1)\cdot \beta$, whereas $\bar{c}$ can be regulated by tuning $(d-1)\cdot T$. This would allow in principle multiple solutions with different *d* values, where of course the popularity fading parameter $\beta$ and the temperature *T* have to be adjusted correspondingly to leave $\gamma$ and $\bar{c}$ unaltered. However, under certain circumstances this freedom in the choice of *d* can become restricted. On the one hand, extreme low required $\gamma$ values impose a lower boundary on the number of dimensions as $d\ge 1+\frac{1}{\gamma -1}$. On the other hand, high required $\bar{c}$ values can impose an upper bound on *d* at a given $\gamma$. Nevertheless, allowing the number of dimensions *d* to take also other values than $d=2$ is certainly increasing our freedom in the choice of the model parameters in general.

### Finding and evaluating communities

Communities are very important structural units in complex networks at the “mesoscopic” scale, without a widely accepted unique definition, but usually associated with subgraphs with a larger internal and a smaller external link density. The automated extraction of communities based solely on the network topology is a challenging problem, with an immense number of different solutions proposed in the literature^9–11^. An interesting related feature of two-dimensional hyperbolic networks is that they also contain communities for the major part of the parameter space, in spite of the lack of any explicit built-in community formation mechanism in the graph generation algorithms^25^. Motivated by this and the overall importance of communities in network science, here we examine also the community structure of *d*-dimensional PSO networks.

Along this line, we apply three independent and well-established community finding algorithms to locate the modules, namely the asynchronous label propagation algorithm (we used the Python function ‘asyn_lpa_communities’ available in the ‘networkx.algorithms.community.label_propagation’ package)^45^, the Infomap method (we used the Python package available at https://pypi.org/project/infomap/)^46^ and the Louvain algorithm (we used the Python implementation available at https://github.com/taynaud/python-louvain)^47^. The basic idea of asynchronous label propagation is to simulate the diffusion of community labels along the examined network, where the regular updating of the labels based on the neighbouring nodes brings a rapid consensus among the members of a dense group on a unique label. In contrast, the Infomap algorithm provides an information-theoretic approach for finding communities, taking advantage of the fact that communities can actively help in achieving the most parsimonious description of the trajectory of an infinitely long random walk on the network. The algorithm itself searches for the minimum of the so-called map equation, which expresses the code length for an average movement in the above-mentioned random walk process. Finally, the Louvain method performs a fast and efficient heuristic maximisation of modularity, which corresponds to the most widely used quality measure for communities^48^, expressed in general as

14 $$\begin{aligned} Q = \frac{1}{2E}\sum _{i=1}^N\sum _{j=1}^N\left[ A_{ij} -P_{ij}\right] \delta _{c_i,c_j}, \end{aligned}$$

where *N* is the number of nodes in the network, $A_{ij}$ denotes an element of the adjacency matrix (${A_{ij}\equiv A_{ji}=1}$ if *i* is connected to *j*, and otherwise $A_{ij}\equiv A_{ji}=0$), $P_{ij}$ gives the connection probability between nodes *i* and *j* in a random null model, *E* stands for the total number of links in the network, $c_i$ is the community to which node *i* belongs and the Kronecker delta $\delta _{c_i,c_j}$ ensures that non-zero contribution can come only from node pairs of the same community. A natural choice for the null model is given by the configuration model, yielding $P_{ij}=\frac{k_ik_j}{2E}$. In the Louvain approach, *Q* is optimised in a hierarchical manner, where after finding the local maximum at a given organisation level of the network, in the next step we move up to the next level by aggregating the current communities into single nodes.

As demonstrated by Fig. 1, a community in a hyperbolic network arises from some inner nodes that serve as community cores and the outer nodes of the corresponding angular sector that are held together by their common preference toward the same attractive centres. As it was detailed in Ref.^25^ in the case of the two-dimensional PSO model, the emergence of a strong community structure can be achieved under two conditions: the existence of inner nodes that are distant from each other enough to provide well-separated attractive centres for the different angular regions, and the localisation of the connections. The distance between the inner community cores can be increased by accelerating their outward drift that simulates the popularity fading via decreasing the popularity fading parameter $\beta$. The strong localisation of the connections can be ensured primarily by setting the temperature *T* to a small value and thus making the cutoff in the connection probability sharp as a function of the hyperbolic distance. However, it has to be also taken into consideration that if the ratio between the number *N* of nodes and the number *m* of connections established by each node at its appearance is smaller, then, to create all the *m* number of links, the nodes are forced more often to connect even to farther nodes. As a consequence, a small temperature in itself is not always enough to make the connections localised, but it has to be complemented with a relatively large *N*/*m* ratio in order to make the connections more strongly determined by the hyperbolic distances and create hereby a more clear separation between the angular regions with respect to the links, thus supporting community formation.

**Figure 5:**
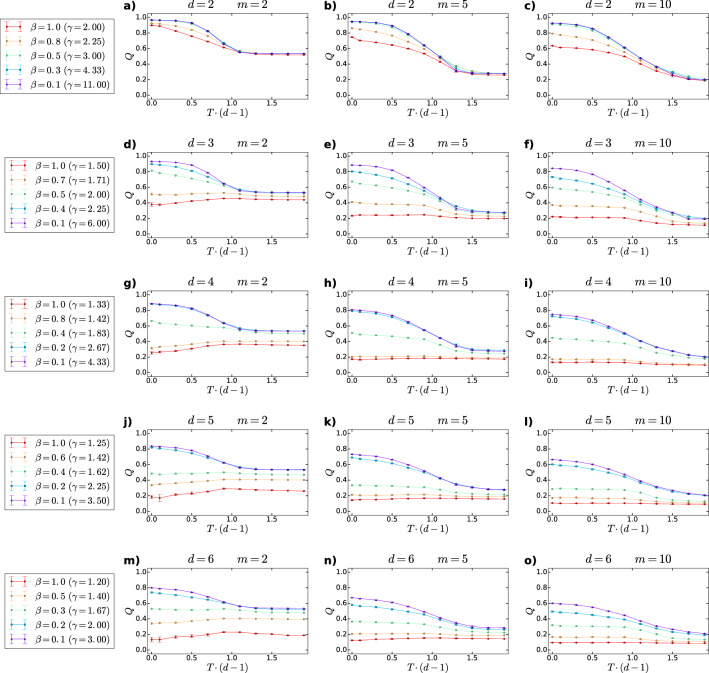
The highest modularity *Q* achieved among the communities obtained by the asynchronous label propagation, the Louvain and the Infomap algorithms in *d*PSO networks, as a function of the rescaled temperature $T\cdot (d-1)$. The dimension *d* is constant across the panel rows (i.e., in panels **a**–**c**, **d**–**f**, **g**–**i**, **j**–**l** and **m**–**o**), whereas the expected average degree 2*m* is constant across the panel columns, as indicated by the panel titles. The different curves in a given subplot correspond to different values of the popularity fading parameter $\beta$ (yielding different degree decay exponents $\gamma$), listed for each dimension in the leftmost panel of the corresponding row. The displayed data points were obtained by averaging over 5 *d*PSO networks of $N=10,000$ nodes at curvature $K=-\zeta ^2=-1$, the error bars indicate the standard deviations.

In Fig. 5, we show the highest modularity *Q* (calculated with the Python function ‘modularity' available in the ‘networkx.algorithms.community.quality' package) obtained among the applied three community finding methods as a function of the rescaled temperature $T\cdot (d-1)$ for *d*PSO networks generated at different parameter settings. In each panel, the bundle of curves (showing *Q* for networks with different degree decay exponents) seems to form a fork-like pattern, in which the curves are more distant from each other in the low-temperature regime and approach each other at high values of $T\cdot (d-1)$, where *Q* becomes more or less constant and independent from the rescaled temperature.

**Figure 6:**
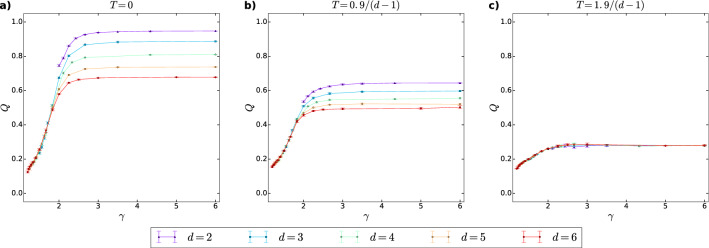
The highest modularity *Q* achieved between the asynchronous label propagation, the Louvain and the Infomap algorithms in *d*PSO networks as a function of the degree decay exponent $\gamma$ at different values of the temperature *T* and the dimension *d*. The panels **a**–**c** refer to different values of the temperature *T*, given in the title of the subplots. The curves of different colours correspond to different number of dimensions *d*, as listed below the panels. The popularity fading parameter was calculated as $\beta =\frac{1}{(d-1)\cdot (\gamma -1)}$. We always set the curvature $K=-\zeta ^2$ of the hyperbolic space to $-1$, the network size *N* to 10, 000 and the expected average degree 2*m* to 10. We searched for communities once with all three community detection methods on 5 *d*PSO networks and plotted the obtained highest modularity averaged over the 5 networks for each parameter setting, with the error bar indicating the standard deviation among the 5 networks.

In Fig. 6, we show the achieved highest modularity *Q* as a function of the degree decay exponent $\gamma$ for different number of dimensions at a low temperature (Fig. 6a), a moderate temperature (Fig. 6b) and a high temperature (Fig. 6c). For all of the examined temperatures, *Q* starts at low values and shows first a strong, then a mild increase toward the higher values of $\gamma$, i.e. as the largest node degrees decrease. The effect of the temperature can be observed by comparing the three panels, where we can see that the constant value to which *Q* settles in the large $\gamma$ regime is higher if *T* is lower, as expected. Furthermore, according to Fig. 6c, when the rescaled temperature $T\cdot (d-1)$ is high enough, the $Q(\gamma )$ curves seem to collapse onto a universal curve for all the examined dimensions, while at smaller temperatures (Fig. 6a,b), the modularity measured at a given degree decay exponent $\gamma$ is a decreasing function of the dimension *d*, similarly to what has been seen on the local scale for the average clustering coefficient in Fig. 4.

## Discussion

The PSO model^21^ is arguably one of the most successful hyperbolic network models since it offers a quite natural way to reproduce the major structural properties of real-world networks. The original formulation of this approach is, however, given only for the two-dimensional hyperbolic disk. Therefore, a question arising naturally in this context is how it can reasonably be extended to higher-dimensional hyperbolic spaces. In the present paper, we studied this issue in detail and introduced the *d*PSO model as a *d*-dimensional generalisation of the original PSO model.

The obtained analytical results show that the scale-free property of *d*PSO networks is inherited from the original PSO model, meaning that the tail of the degree distribution decays as a power-law, $\displaystyle {\lim _{k \rightarrow \infty }} \mathcal {P}(k)\sim k^{-\gamma }$, for any number of dimensions $d\ge 2$. The exponent $\gamma$ is determined by the popularity fading parameter $\beta$ and the number of dimensions *d* via the formula $\gamma =1+\frac{1}{(d-1)\cdot \beta }$, which can be basically explained as follows. The solid angle subtended by the entire surface of the *d*-dimensional ball representing the *d*-dimensional hyperbolic space is an increasing function of the number of dimensions, meaning that in the case of a higher *d*, the uniform distribution of the same number of nodes on the surface results in larger angular distances between the nearest neighbours. Therefore, at a given popularity fading parameter (controlling the attractiveness of the early-appeared nodes arising from their relatively low radial coordinate), in higher dimensions we see an increased propensity of the newly coming nodes to connect to the innermost nodes of any angular position instead of the angular neighbours located at larger radii. Due to this, the same popularity fading parameter $\beta$ yields larger maximum degree, and thus, smaller degree decay exponent $\gamma$ for larger values of *d*. To compensate for the decrease in the attractiveness of the angular neighbours caused by the increase in the number of dimensions and keep the degree decay exponent $\gamma$ at a given value, one has to reduce the distinguished attractiveness of the inner nodes and limit the angular range in which they are preferred. For this, the advantage of the inner nodes in their radial position has to be decreased by shifting them more outwards, which can be achieved by setting the popularity fading parameter $\beta$ to a smaller value and enhancing thereby the process of popularity fading.

It is worth emphasising that the smallest attainable degree decay exponent (obtained at $\beta =1$) is 2 in the original PSO model^21^, whereas in *d*PSO networks

15 $$\begin{aligned} \gamma _{\text {min}}(d)=1+\frac{1}{d-1}, \end{aligned}$$

which can be decreased below the two-dimensional limit $\gamma _{\text {min}}(2)=2$ by simply increasing the number of dimensions *d* above 2. Note that the original formulation of the *d*-dimensional RHG model as it is introduced in Refs.^42,43^ does not allow the generation of such heavy-tailed networks with $\gamma <2$. Nevertheless, with an appropriate modification of the radial coordinate distribution $\rho (r)$, one can obtain a wide range of degree distributions within this model as well, similarly to what has been shown for the $\mathbb {S}^1/\mathbb {H}^2$ model in Ref.^27^. The analysis of scale-free networks with a degree decay exponent smaller than two is in itself an interesting topic since such networks exhibit many exotic features that can not be observed in scale-free networks with $2\le \gamma$, including the divergence of the average degree or the presence of macroscopical hubs being connected to a finite fraction of the nodes even in the thermodynamic limit $N\rightarrow \infty$^49,50^.

In terms of *d*PSO networks, we have found that such extremely skewed degree distributions lead to further unexpected results. As indicated in Figs. 4 and 6, *d*PSO networks of $\gamma <2$ are characterised by high average clustering coefficient $\bar{c}$, but remarkably at the same time relatively small modularity *Q* at basically any temperature *T*. Intuitively, this behaviour can be understood if we consider that on the one hand, since the largest hubs are formed from the first few nodes of the network generation process, they are densely connected to each other. Thus, in the presence of extremely large hubs to which most of the nodes connect at their appearance, triangles are formed with large probability, resulting in a large $\bar{c}$. On the other hand, these large hubs make the partitioning of the network into disjunct communities with high modularity practically impossible since the community of any such hub has a macroscopic number of links pointing outside of the given community, resulting in low *Q* values.

In addition, we found that the number of dimensions *d* along with the temperature *T* play a joint role in controlling the average clustering coefficient $\bar{c}$ of *d*PSO networks as well. More precisely, as it can be seen in Fig. 3, we can distinguish two phases separated by the critical point $T_{\text {c}}=\frac{1}{d-1}$, where the average clustering coefficient of the networks behaves in a fundamentally different way. At temperatures $T<T_{\text {c}}$, $\bar{c}$ is a decreasing function of the temperature; however, at temperatures near and above $T_{\text {c}}$, $\bar{c}$ tends to become independent from *T*. Besides, based on Fig. 4, at low temperatures we can state that the average clustering coefficient measured at a given degree decay exponent $\gamma$ decreases with the number of dimensions *d*, in perfect accordance with the results found in Ref.^51^ for the RHG model. Meanwhile, near the critical temperature $\bar{c}$ begins to show a universal decay with $\gamma$, irrespectively of the separate values of the popularity fading parameter $\beta$ and the dimension *d*.

Remarkably, a similar separation of phases with the same critical temperature has also been observed in the *d*-dimensional RHG model^42,43^. Although the *d*-dimensional PSO and RHG models share some other common characteristics, such as the Fermi-Dirac connection probability or the uniform angular distribution of the nodes, they fairly differ from each other. One of the most significant differences is apparently the process of network construction: the *d*-dimensional RHG model generates static hyperbolic graphs with a fixed number of nodes, while the *d*PSO model simulates the growth of networks in the hyperbolic space, thus providing a dynamic network model. The latter can be advantageous for embeddings by naturally allowing the calculation of local likelihoods instead of the optimisation of global values^22^. Another difference is that the radial coordinates *r* of the nodes are sampled from a predefined distribution $\rho (r)$ in the *d*-dimensional RHG model^43^, whereas in the case of *d*PSO networks a logarithmically increasing radial sequence of the nodes is imposed. This implies that in the *d*-dimensional RHG model two distinct nodes can have the same radial coordinates, a phenomenon which is impossible in the *d*PSO model. A somewhat less beneficial property of *d*-dimensional RHG networks is that they are not necessarily sparse: when $\gamma =2$ and the temperature *T* is below $1/(d-1)$, the expected average degree of the network scales with the system size as $\left\langle k \right\rangle \sim \ln ^2 (N)$^43^. In contrast to this, the expected average degree of *d*PSO networks is given by $\left\langle k \right\rangle =2m$; therefore, their sparseness can always be assured by controlling the parameter *m*.

As it has already been reported in Ref.^25^, the original, two-dimensional PSO model^21^ is capable of generating networks with strong communities for a wide range of the parameter settings, despite the fact that it does not include any explicitly built-in community structure generating mechanism. Based on the high modularity values measured on *d*PSO networks (Figs. 5 and 6), here we conclude that the emergence of a strong community structure is not a peculiar feature of the two-dimensional case, but it can be observed even if the number of dimensions is larger than 2, provided that the degree decay exponent $\gamma$ is not too small and the temperature *T* is not too high. Nevertheless, at such settings of $\gamma$ and *T*, the modularity *Q* obtained at a given $\gamma$ decreases as *d* is increased, similarly to the average clustering coefficient $\bar{c}$.

In conclusion, motivated by the fact that there is evidently no particular reason to assume that the underlying hyperbolic space of complex networks is certainly two-dimensional, here we proposed a generalisation of the two-dimensional popularity-similarity optimisation model of network growth, namely the *d*PSO model, in which the dimension *d* acts as an additional degree of freedom. A further motivation for higher-dimensional hyperbolic models is given by higher-dimensional embedding methods, where the behaviour of the likelihood optimisation can be more favourable compared to the traditional approaches working in two dimensions. In our studies, we found that *d*PSO networks are suitable for capturing many essential characteristics of real-world networks such as the scale-free property, the high value of the average clustering coefficient or the strong community structure. Namely, *d*PSO networks generated in lower-dimensional hyperbolic spaces using not too high temperatures and not too low degree decay exponents simultaneously exhibit all the above-mentioned features of real-world networks. Nevertheless, as the number of dimensions *d* increases, both the maximal average clustering coefficient and the maximal modularity that can be obtained at a given degree decay exponent $\gamma$ (by setting the temperature *T* to 0) decreases. This implies that *d*PSO networks generated in high-dimensional spaces (e.g., at $d>10$) can be characterised by neither a clustering nor a community structure of similar strength that can be observed in various real-world examples; hence, a reasonable upper limit can be found on the number of dimensions of the hyperbolic space that underlies real networks. By following similar considerations regarding the clustering coefficient of RHG networks, in Ref.^52^ the authors claim that in terms of both the modelling and the embedding techniques, the most suitable choice for the number of dimensions of the underlying hyperbolic space is $d=2$. Here, we complement this result by showing that in general, the *d*PSO model with *d* slightly above 2 also performs excellently on the modelling ground (still yielding relatively high values of the average clustering coefficient and the modularity), which, along with the recent success of $d>2$ hyperbolic embedding techniques for instance in link prediction and in graph reconstruction^39^ or the separation of communities^40^, confirmes the relevance of low-dimensional hyperbolic spaces (e.g. with $d=3$ or $d=4$) in the theory of complex networks. In Ref.^38^, several different methods have already been provided for assigning angular coordinates to the network nodes in hyperbolic spaces of arbitrary curvature $K=-\zeta ^2$ and number of dimensions *d*. Using the angular positions yielded by one of these methods, in order to create an embedding that corresponds to our *d*PSO model, the radial coordinate of the node having the $\ell$th ($\ell =1,2,...,N$) largest degree (with ties in the order of node degrees broken arbitrarily) has to be calculated as $r_{\ell N}=\beta \cdot (2/\zeta )\cdot \ln {\ell }+(1-\beta )\cdot (2/\zeta )\cdot \ln {N}$, where the popularity fading parameter $\beta$ is determined by the degree decay exponent $\gamma$ and the number of dimensions *d* as $\beta =\frac{1}{(d-1)\cdot (\gamma -1)}$.

## Supplementary Information

Supplementary Information.

## Data Availability

All data generated during the current study are available from the corresponding author upon request.

## Code availability

The code used for generating networks with the *d*-dimensional PSO model will be available at https://github.com/BianKov/dPSO upon publication.
