# Supplementary material for: Generalised popularity-similarity optimisation model for growing hyperbolic networks beyond two dimensions
Source: Sci Rep. 2022 Jan 19;12:968. doi: 10.1038/s41598-021-04379-1 (PMC8770586; doi:10.1038/s41598-021-04379-1)
Supplement: Supplementary file 1 — Supplementary Information. [file 41598_2021_4379_MOESM1_ESM.pdf]

Generalised popularity-similarity optimisation model for growing hyperbolic networks beyond two dimensions – Supplementary Information

Bianka Kovács[1], Sámuel G. Balogh[1,*], and Gergely Palla[1,2,3]

[1]Dept. of Biological Physics, Eötvös Loránd University, H-1117 Budapest, Pázmány P. stny. 1/A, Hungary
[2]MTA-ELTE Statistical and Biological Physics Research Group, H-1117 Budapest, Pázmány P. stny. 1/A, Hungary
[3]Health Services Management Training Centre, Semmelweis University, H-1125, Kútvölgyi út 2, Budapest, Hungary
[*]balogh@hal.elte.hu

S1 Degree distribution of dPSO networks

It has been shown in Ref. [1] that in the two-dimensional PSO model of expected average degree $\bar{k} \approx 2 \cdot m$ and popularity fading parameter β, the probability that node i (appearing at time i) and node j (appearing at time $j > i$) connect to each other can be written as

$$\Pi(i,j) = m \cdot \frac{i^{-\beta}}{\int_1^j i^{-\beta}\,\mathrm{d}i}. \tag{S1.1}$$

The preferential attachment model in Ref. [2] yields the same connection probability with $\beta = 1/(\gamma - 1)$ when the number of connections created at the appearance of a new node is set to m and the degree distribution $\mathcal{P}(K = k)$ is proportional to $k^{-\gamma}$. In this section we show that in the d-dimensional popularity-similarity optimisation model

$$\Pi(i,j) = m \cdot \frac{i^{-(d-1)\cdot\beta}}{\int_1^j i^{-(d-1)\cdot\beta}\,\mathrm{d}i}, \tag{S1.2}$$

which – according to the analogy with the preferential attachment model – means that the degree distribution of the networks generated by the dPSO model takes the form of $\mathcal{P}(K = k) \sim k^{-\gamma}$ with $\gamma = 1 + \frac{1}{(d-1)\cdot\beta}$. Sect. S1.1 deals with the case of $T = 0$, where the new node always connects to the m hyperbolically closest nodes, while Sect. S1.2 describes settings where the temperature is strictly larger than zero ($0 < T$), enabling hyperbolically father nodes to become connected as well.

S1.1 Connection probability in the case of deterministic connection

At temperature $T = 0$, each node $j = 1, 2, ..., N$ connects at its appearance to the m hyperbolically closest nodes or, in other words, to all the previously appeared nodes that lie from node j within a certain hyperbolic distance R_j. Thus, the probability $\Pi(i,j)$ of the emergence of a link between nodes i and j (with $i < j$) equals to the probability that the hyperbolic distance of node i from node j at the appearance of the latter is not larger than R_j, i.e.

$$\Pi(i,j) = P(x_{ij}(j) \leq R_j), \tag{S1.1.1}$$

where R_j is determined by the equation

$$m = \int_1^j \Pi(i,j)\,\mathrm{d}i \tag{S1.1.2}$$

expressing that the expected number of the previously appeared nodes that connect to node j must be m. The hyperbolic distance between nodes i and j at time j can be approximated as

$$x_{ij}(j) \approx r_{ij} + r_{jj} + \frac{2}{\zeta} \cdot \ln\left(\frac{\theta_{ij}}{2}\right), \tag{S1.1.3}$$

assuming that ζr_{ij} and ζr_{jj} are sufficiently large (which is true for most of the network nodes if the total number of nodes N is large enough) and therefore, $2 \cdot \sqrt{e^{-2\zeta r_{ij}} + e^{-2\zeta r_{jj}}} < \theta_{ij}$, but in the meantime the angular distance between the possibly

connecting nodes i and j is small enough to use the approximation $\sin(\theta_{ij}/2) \approx \theta_{ij}/2$ [3]. Using Eq. (S1.1.3), the connection probability of nodes i and j can be written for $T=0$ as

$$\Pi(i,j) = P(x_{ij}(j) \leq R_j) = P\left(\theta_{ij} \leq 2 \cdot e^{-\frac{\zeta}{2}\cdot(r_{ij}+r_{jj}-R_j)}\right), \tag{S1.1.4}$$

i.e., we are searching for the probability that the angular distance between nodes i and j is not larger than a given value, namely $\theta_{ij}^{\max} = 2 \cdot e^{-\frac{\zeta}{2}\cdot(r_{ij}+r_{jj}-R_j)}$. This can be rephrased as the probability that the angular position of node i falls in the spherical sector characterised by an apex angle $2 \cdot \theta_{ij}^{\max}$ and an axis going through node j.

Since the angular position of the network nodes is chosen uniformly at random on the surface of a d-dimensional ball, the probability that the angular location of a given node falls in a certain spherical sector can be written simply as the fraction of the solid angle subtended by the sector in question and the solid angle subtended by the complete d-dimensional ball, independently of the direction of the axis of the examined spherical sector. Consequently, the probability that the angular distance measured between a new node and a previously appeared node is not larger than a given value $\theta^{\max}$ equals to the solid angle subtended by a spherical sector of apex angle $2 \cdot \theta^{\max}$ divided by the solid angle subtended by the complete ball. Thus,

$$\Pi(i,j) = \frac{\Omega_d\left(2 \cdot e^{-\frac{\zeta}{2}\cdot(r_{ij}+r_{jj}-R_j)}\right)}{\Omega_d^{\text{total}}} = \frac{\Omega_{d-1}^{\text{total}}}{\Omega_d^{\text{total}}} \cdot \int_0^{2\cdot e^{-\frac{\zeta}{2}\cdot(r_{ij}+r_{jj}-R_j)}} \sin^{d-2}\phi \, \mathrm{d}\phi, \tag{S1.1.5}$$

where Ω_d^{total} denotes the solid angle subtended by the complete surface of a d-dimensional ball, namely

$$\Omega_d^{\text{total}} = \begin{cases} \frac{2\cdot\pi^{\frac{d}{2}}}{\left(\frac{d}{2}-1\right)!} & \text{if } d \text{ is even,} \\ \\ \frac{\frac{d-1}{2}!\cdot 2^d \cdot \pi^{\frac{d-1}{2}}}{(d-1)!} & \text{if } d \text{ is odd,} \end{cases} \tag{S1.1.6}$$

and in the last step we used that the solid angle subtended by a d-dimensional spherical sector of an apex angle 2ψ can be calculated as

$$\Omega_d(\psi) = \Omega_{d-1}^{\text{total}} \cdot \int_0^{\psi} \sin^{d-2}\phi \, \mathrm{d}\phi. \tag{S1.1.7}$$

In the case of large networks, the maximum angular distance $\theta_{ij}^{\max} = 2 \cdot e^{-\frac{\zeta}{2}\cdot(r_{ij}+r_{jj}-R_j)}$ that still enables the link formation is small enough for most of the nodes to assume that in the range of the integration in Eq. (S1.1.5), $\sin^{d-2}\phi \approx \phi^{d-2}$. Based on this,

$$\Pi(i,j) \approx \frac{\Omega_{d-1}^{\text{total}}}{\Omega_d^{\text{total}}} \cdot \int_0^{2\cdot e^{-\frac{\zeta}{2}\cdot(r_{ij}+r_{jj}-R_j)}} \phi^{d-2} \, \mathrm{d}\phi = \frac{\Omega_{d-1}^{\text{total}}}{\Omega_d^{\text{total}}} \cdot \left[\frac{\phi^{d-1}}{d-1}\right]_0^{2\cdot e^{-\frac{\zeta}{2}\cdot(r_{ij}+r_{jj}-R_j)}} = \frac{\Omega_{d-1}^{\text{total}}}{\Omega_d^{\text{total}}} \cdot \frac{2^{d-1} \cdot e^{-\frac{\zeta\cdot(d-1)}{2}\cdot(r_{ij}+r_{jj}-R_j)}}{d-1}. \tag{S1.1.8}$$

Introducing the notation

$$\eta(d) = \frac{\Omega_{d-1}^{\text{total}}}{\Omega_d^{\text{total}}} = \begin{cases} \frac{\left(\frac{d}{2}-1\right)!\cdot\frac{d-2}{2}!\cdot 2^{d-2}}{(d-2)!\cdot\pi} & \text{if } d \text{ is even,} \\ \\ \frac{(d-1)!}{\left(\frac{d-1}{2}-1\right)!\cdot\frac{d-1}{2}!\cdot 2^{d-1}} & \text{if } d \text{ is odd,} \end{cases} \tag{S1.1.9}$$

we arrive at the formula

$$\Pi(i,j) = \eta(d) \cdot \frac{2^{d-1}}{d-1} \cdot e^{-\frac{\zeta\cdot(d-1)}{2}\cdot(r_{ij}+r_{jj}-R_j)} \tag{S1.1.10}$$

of the probability that nodes i and j get connected during the network growth.

According to the model definition, here the initial radial coordinate of the jth node is $r_{jj} = (2/\zeta)\ln j$ and the radial coordinate of node i at time j is $r_{ij} = \beta \cdot r_{ii} + (1-\beta)\cdot r_{jj} = \beta \cdot (2/\zeta)\ln i + (1-\beta)\cdot(2/\zeta)\ln j$, while R_j can be expressed from the equation

$$m = \eta(d) \cdot \frac{2^{d-1}}{d-1} \cdot e^{-\frac{\zeta\cdot(d-1)}{2}\cdot(2-\beta)\cdot r_{jj}} \cdot e^{\frac{\zeta\cdot(d-1)}{2}\cdot R_j} \cdot \int_1^j e^{-\frac{\zeta\cdot(d-1)}{2}\cdot\beta\cdot r_{ii}} \, \mathrm{d}i \tag{S1.1.11}$$

obtained by substituting (S1.1.10) into Eq. (S1.1.2). After some rearrangement, we can express R_j as

$$R_j = \frac{2}{\zeta\cdot(d-1)}\cdot\ln\left(\frac{(d-1)\cdot m}{\eta(d)\cdot 2^{d-1}\cdot e^{-\frac{\zeta\cdot(d-1)}{2}\cdot(2-\beta)\cdot r_{jj}}\cdot\int_1^j e^{-\frac{\zeta\cdot(d-1)}{2}\cdot\beta\cdot r_{ii}}\,\mathrm{d}i}\right). \tag{S1.1.12}$$

Substituting this expression back into (S1.1.10) yields

$$\Pi(i,j) = m\cdot\frac{e^{-\frac{\zeta\cdot(d-1)}{2}\cdot\beta\cdot r_{ii}}}{\int_1^j e^{-\frac{\zeta\cdot(d-1)}{2}\cdot\beta\cdot r_{ii}}\,\mathrm{d}i} = m\cdot\frac{i^{-(d-1)\cdot\beta}}{\int_1^j i^{-(d-1)\cdot\beta}\,\mathrm{d}i}. \tag{S1.1.13}$$

Hereby we proved that the probability that nodes i and j connect to each other indeed can be written in the form of Eq. (S1.2) at $T=0$.

S1.2 Connection probability in the case of $0<T$

At temperature $0<T$, the newly appearing node j ($j=1,2,...,N$) repeatedly makes attempts to create connections with the previously appeared nodes (indexed by $i=1,2,...,j-1$) until the emergence of m number of links. Thus, using the probability $P(i,j)$ that the new node j connects in a given connection attempt to node i and the probability $P(j)=\int_1^j P(i,j)\,\mathrm{d}i$ that the new node j connects in a given connection attempt to any of the already existing nodes, the probability that node i becomes connected to node j can be written as

$$\Pi(i,j) = m\cdot\frac{P(i,j)}{P(j)}. \tag{S1.2.1}$$

In one connection attempt, the new node j chooses randomly one of the previously appeared nodes, each with probability $1/(j-1)$, and if there is still no connection between the new node and the selected one, then the link formation occurs with probability $p(x)=1/\left[1+e^{\zeta(x-R_j)/(2T)}\right]$, given that the current hyperbolic distance between the two nodes in question equals to x. Notice that in the case of large networks, most of the nodes appear at times $j\gg m$, when the probability that the new node selects such a random node to which it is already connected is insignificant and can be ignored to ease the analysis. However, it has to be taken into consideration that – contrary to the radial coordinates – the angular coordinates of the nodes are not strictly determined by the node identifiers, but are random variables; therefore, the hyperbolic distance between two nodes is also a random variable, and the probability that a given link creation attempt succeeds can be formulated as $\int_0^\infty p(x)\cdot P(x_{ij}(j)=x)\,\mathrm{d}x$. This can be rewritten as $\int_0^\pi p(x)\cdot P(\theta_{ij}=\phi)\,\mathrm{d}\phi$, since for given nodes with given radial coordinates the only source of randomness in the hyperbolic distance is the angular distance θ_{ij} between the nodes. As described in Sect. S1.1, due to the uniformity of the angular node arrangement, the probability that the angular distance between two nodes falls in the range $[\phi,\phi+\mathrm{d}\phi)$ can be calculated by dividing the solid angle subtended by the volume enclosed between two coaxial spherical sectors of apex angles $2\cdot\phi$ and $2\cdot(\phi+\mathrm{d}\phi)$ by the solid angle subtended by the complete d-dimensional ball, i.e.,

$$P(\theta_{ij}=\phi)\,\mathrm{d}\phi = P(\phi\le\theta_{ij}<\phi+\mathrm{d}\phi) = \frac{\Omega_d(\phi+\mathrm{d}\phi)-\Omega_d(\phi)}{\Omega_d^{\mathrm{total}}} = \frac{\frac{\mathrm{d}\Omega_d}{\mathrm{d}\phi}\,\mathrm{d}\phi}{\Omega_d^{\mathrm{total}}}. \tag{S1.2.2}$$

Using Eq. (S1.1.7) and that

$$\frac{\mathrm{d}}{\mathrm{d}y}\int_{f(y)}^{g(y)} h(z)\mathrm{d}z = \frac{\mathrm{d}g}{\mathrm{d}y}\cdot h(g(y)) - \frac{\mathrm{d}f}{\mathrm{d}y}\cdot h(f(y)), \tag{S1.2.3}$$

we arrive at the formula

$$P(\theta_{ij}=\phi)\,\mathrm{d}\phi = \frac{\Omega_{d-1}^{\mathrm{total}}\cdot\sin^{d-2}\phi\,\mathrm{d}\phi}{\Omega_d^{\mathrm{total}}}, \tag{S1.2.4}$$

which, using the notation introduced in Eq. (S1.1.9), takes the form of

$$P(\theta_{ij}=\phi)\,\mathrm{d}\phi = \eta(d)\cdot\sin^{d-2}\phi\,\mathrm{d}\phi. \tag{S1.2.5}$$

All things considered, the probability that node i attracts a link from node j in a given connection attempt can be formulated as

$$P(i,j) = \frac{\eta(d)}{j-1}\cdot\int_0^\pi\frac{\sin^{d-2}\phi}{1+e^{\frac{\zeta(x-R_j)}{2T}}}\,\mathrm{d}\phi. \tag{S1.2.6}$$

Using that at the arrival of node j its hyperbolic distance from node i can be written as [3]

$$x_{ij}(j) \approx r_{ij} + r_{jj} + \frac{2}{\zeta} \cdot \ln\left(\sin\left(\frac{\theta_{ij}}{2}\right)\right), \tag{S1.2.7}$$

we arrive at the formula

$$P(i,j) = \frac{\eta(d)}{j-1} \cdot \int_0^\pi \frac{\sin^{d-2}\phi}{1 + \left(e^{\frac{\zeta}{2}\cdot(r_{ij}+r_{jj}-R_j)} \cdot \sin\left(\frac{\phi}{2}\right)\right)^{\frac{1}{T}}} \mathrm{d}\phi. \tag{S1.2.8}$$

If the temperature T is small enough, then in the case of sufficiently large networks we can assume for most of the node pairs that the main contribution of the above integral comes from the range of small angular distances. This implies that the approximation $\sin\phi \approx \phi$ can be used, and after that, changing the upper limit of the integral from π to infinity practically does not affect the value of the integral. Using these two assumptions, the connection probability becomes

$$P(i,j) \approx \frac{\eta(d)}{j-1} \cdot \int_0^\infty \frac{\phi^{d-2}}{1 + \left(e^{\frac{\zeta}{2}\cdot(r_{ij}+r_{jj}-R_j)} \cdot \frac{\phi}{2}\right)^{\frac{1}{T}}} \mathrm{d}\phi =$$
$$= \frac{\eta(d)}{j-1} \cdot 2^{d-1} \cdot T \cdot \Gamma((d-1)\cdot T) \cdot \Gamma(1-(d-1)\cdot T) \cdot \frac{1}{\left(e^{\frac{\zeta}{2}\cdot(r_{ij}+r_{jj}-R_j)}\right)^{d-1}}. \tag{S1.2.9}$$

Finally, the substitution of Euler's reflection formula $\Gamma(z)\cdot\Gamma(1-z) = \pi/\sin(z\cdot\pi)$ and the radial coordinate formulas $r_{jj} = (2/\zeta)\ln j$ and $r_{ij} = \beta \cdot r_{ii} + (1-\beta)\cdot r_{jj} = \beta\cdot(2/\zeta)\ln i + (1-\beta)\cdot(2/\zeta)\ln j$ yields

$$P(i,j) \approx \frac{\eta(d)\cdot\pi\cdot 2^{d-1}\cdot T}{(j-1)\cdot\sin((d-1)\cdot T\cdot\pi)} \cdot \frac{1}{\left(e^{\frac{\zeta}{2}\cdot(r_{ij}+r_{jj}-R_j)}\right)^{d-1}} =$$
$$= \frac{\eta(d)\cdot\pi\cdot 2^{d-1}\cdot T}{(j-1)\cdot\sin((d-1)\cdot T\cdot\pi)} \cdot j^{(d-1)\cdot(\beta-2)} \cdot i^{-(d-1)\cdot\beta} \cdot e^{\frac{\zeta\cdot(d-1)}{2}\cdot R_j}, \tag{S1.2.10}$$

which gives back for $d=2$ the result of Ref. [1] in the $0<T<1$ case.

Note that since $\Gamma(1-(d-1)\cdot T)$ is not defined for $T = 1/(d-1)$ and becomes negative as T exceeds $1/(d-1)$, the approximation in Eq. (S1.2.9) can be valid only in the case of $T < 1/(d-1)$. However, at least in the case of sufficiently large networks, one can neglect the 1 in the denominator of the formula S1.2.8 for most of the node pairs at higher temperatures, yielding

$$P(i,j) \approx \frac{\eta(d)}{j-1} \cdot \int_0^\pi \frac{\sin^{d-2}\phi}{\sin^{\frac{1}{T}}\left(\frac{\phi}{2}\right)} \mathrm{d}\phi \cdot \frac{1}{\left(e^{\frac{\zeta}{2}\cdot(r_{ij}+r_{jj}-R_j)}\right)^{\frac{1}{T}}}. \tag{S1.2.11}$$

This approximation can hold only for $1/(d-1) < T$, as otherwise the integral $\int_0^\pi \sin^{d-2}(\phi)\cdot\sin^{-1/T}(\phi/2)\,\mathrm{d}\phi$ is divergent since $\lim_{\phi\to 0}\sin^{d-2}(\phi)\cdot\sin^{-1/T}(\phi/2) = 2^{1/T}\cdot\phi^{d-2-1/T}$ increases slower than ϕ^{-1} towards $\phi\to 0$ only if $1/(d-1) < T$. To ensure that the term of the connection probability $P(i,j)$ depending on the smaller node index i remains the same for $1/(d-1) < T$ as in the case of $0 \le T < 1/(d-1)$, the initial radial coordinate of each node $\ell \ge 1$ must be set to $r_{\ell\ell} = (2T(d-1)/\zeta)\cdot\ln\ell$ instead of $r_{\ell\ell} = (2/\zeta)\cdot\ln\ell$. This means that in Eq. (S1.2.11) $r_{jj} = (2T(d-1)/\zeta)\cdot\ln j$ and $r_{ij} = \beta\cdot r_{ii} + (1-\beta)\cdot r_{jj} = \beta\cdot(2T(d-1)/\zeta)\ln i + (1-\beta)\cdot(2T(d-1)/\zeta)\ln j$. Thus,

$$P(i,j) \approx \frac{\eta(d)}{j-1} \cdot \int_0^\pi \frac{\sin^{d-2}\phi}{\sin^{\frac{1}{T}}\left(\frac{\phi}{2}\right)} \mathrm{d}\phi \cdot j^{(d-1)\cdot(\beta-2)} \cdot i^{-(d-1)\cdot\beta} \cdot e^{\frac{\zeta}{2\cdot T}\cdot R_j}, \tag{S1.2.12}$$

which, using also the approximation $\sin(\phi/2) \approx \phi/2$, can be written for $d=2$ as

$$P(i,j) \approx \frac{1/\pi}{j-1} \cdot 2^{\frac{1}{T}} \cdot \int_0^\pi \phi^{-\frac{1}{T}} \mathrm{d}\phi \cdot j^{\beta-2} \cdot i^{-\beta} \cdot e^{\frac{\zeta}{2\cdot T}\cdot R_j} = \frac{1/\pi}{j-1} \cdot 2^{\frac{1}{T}} \cdot \frac{\pi^{1-1/T}}{1-1/T} \cdot j^{\beta-2} \cdot i^{-\beta} \cdot e^{\frac{\zeta}{2\cdot T}\cdot R_j} =$$
$$= \left(\frac{2}{\pi}\right)^{\frac{1}{T}} \cdot \frac{T}{(j-1)\cdot(T-1)} \cdot j^{\beta-2} \cdot i^{-\beta} \cdot e^{\frac{\zeta}{2\cdot T}\cdot R_j}, \tag{S1.2.13}$$

corresponding to the result of Ref. [1] in the case of $1 < T$.

Based on Eqs. (S1.2.10) and (S1.2.12), the probability that node j connects to any of the previously appeared nodes in a given link formation attempt is

$$P(j) = \int_1^j P(i,j)\,\mathrm{d}i = C \cdot \int_1^j i^{-(d-1)\cdot\beta}\,\mathrm{d}i = C \cdot \frac{j^{1-(d-1)\cdot\beta} - 1}{1-(d-1)\cdot\beta} \tag{S1.2.14}$$

with

$$C = \frac{P(i,j)}{i^{-(d-1)\cdot\beta}} = \begin{cases} \frac{\eta(d)\cdot\pi\cdot 2^{d-1}\cdot T}{(j-1)\cdot\sin((d-1)\cdot T\cdot\pi)} \cdot j^{(d-1)\cdot(\beta-2)} \cdot e^{\frac{\zeta\cdot(d-1)}{2}\cdot R_j} & \text{if } T < \frac{1}{d-1}, \\ \frac{\eta(d)}{j-1} \cdot \int_0^\pi \frac{\sin^{d-2}\phi}{\sin^{\frac{1}{T}}\left(\frac{\phi}{2}\right)}\,\mathrm{d}\phi \cdot j^{(d-1)\cdot(\beta-2)} \cdot e^{\frac{\zeta}{2\cdot T}\cdot R_j} & \text{if } \frac{1}{d-1} < T \end{cases} \tag{S1.2.15}$$

denoting the term in $P(i,j)$ that is independent of the node index i.

Finally, using Eqs. (S1.2.1), (S1.2.14) and (S1.2.15), the probability that nodes i and j connect to each other can be written as

$$\Pi(i,j) = m \cdot \frac{P(i,j)}{P(j)} = m \cdot \frac{C \cdot i^{-(d-1)\cdot\beta}}{C \cdot \int_1^j i^{-(d-1)\cdot\beta}\,\mathrm{d}i} = m \cdot \frac{i^{-(d-1)\cdot\beta}}{\int_1^j i^{-(d-1)\cdot\beta}\,\mathrm{d}i}, \tag{S1.2.16}$$

which is the same formula as the one given by Eq. (S1.2) that we wanted to prove.

S1.3 Replacement of the sinus functions by their argument in higher-dimensional connection probabilities

Although in earlier works on two-dimensional hyperbolic networks it became a common procedure [1, 3, 4, 5] to replace $\sin(\phi/2)$ by $\phi/2$ in the hyperbolic distance formula

$$x_{ij}(j) \approx r_{ij} + r_{jj} + \frac{2}{\zeta} \cdot \ln\left(\sin\left(\frac{\theta_{ij}}{2}\right)\right), \tag{S1.3.1}$$

it is important to clarify that the approximation $\sin\phi \approx \phi$ (already used for d-dimensional hyperbolic networks in Ref. [6]) is also well applicable in the higher-dimensional connection probabilities. In Fig. S1.3.1, we compare at temperature $T = 0.5/(d-1)$ the integrand

$$I_{\text{sinAngle}} = \frac{\sin^{d-2}\phi}{1 + \left(z \cdot \sin\left(\frac{\phi}{2}\right)\right)^{\frac{1}{T}}} \tag{S1.3.2}$$

of Eq. (S1.2.8) with its approximated form

$$I_{\text{Angle}} = \frac{\phi^{d-2}}{1 + \left(z \cdot \frac{\phi}{2}\right)^{\frac{1}{T}}} \tag{S1.3.3}$$

given in Eq. (S1.2.9). For the sake of simplicity, here we introduced the notation $z = e^{\frac{\zeta}{2}\cdot(r_{ij}+r_{jj}-R_j)}$, denoting a term that is an increasing function of the node indexes i and j. As such, for networks of larger number of nodes N, the value of z becomes large for a higher proportion of the node pairs. According to Fig. S1.3.1, the replacement of the sinus functions by their argument works well even at a relatively low z if the considered angles are small enough. However, as z increases, the range of proper angles broadens at each examined d, suggesting that one can find for any number of dimensions high enough values of z at which the approximation of the integrand is valid for the angular distances between the nodes that have the potential to become connected during the network growth.

Note that in the case of networks of a given number of nodes N, towards the higher-dimensional spaces the angular distance between the (possibly connecting) angular neighbours becomes larger and can fall out of the range that is well approximable for a given value of z. Besides, for networks of a given degree decay exponent γ, the popularity fading parameter $\beta = \frac{1}{(d-1)\cdot(\gamma-1)}$ decreases with the increasing number of dimensions d, resulting in smaller values of $z = e^{\frac{\zeta}{2}\cdot(r_{ij}+r_{jj}-R_j)}$ for a given N, i.e. a tighter range of angular distances that is proper for the approximation. Nevertheless, as it is shown in Fig. S1.3.2, if the number of nodes N is increased simultaneously with the number of dimensions d, the degree distributions measured on dPSO networks

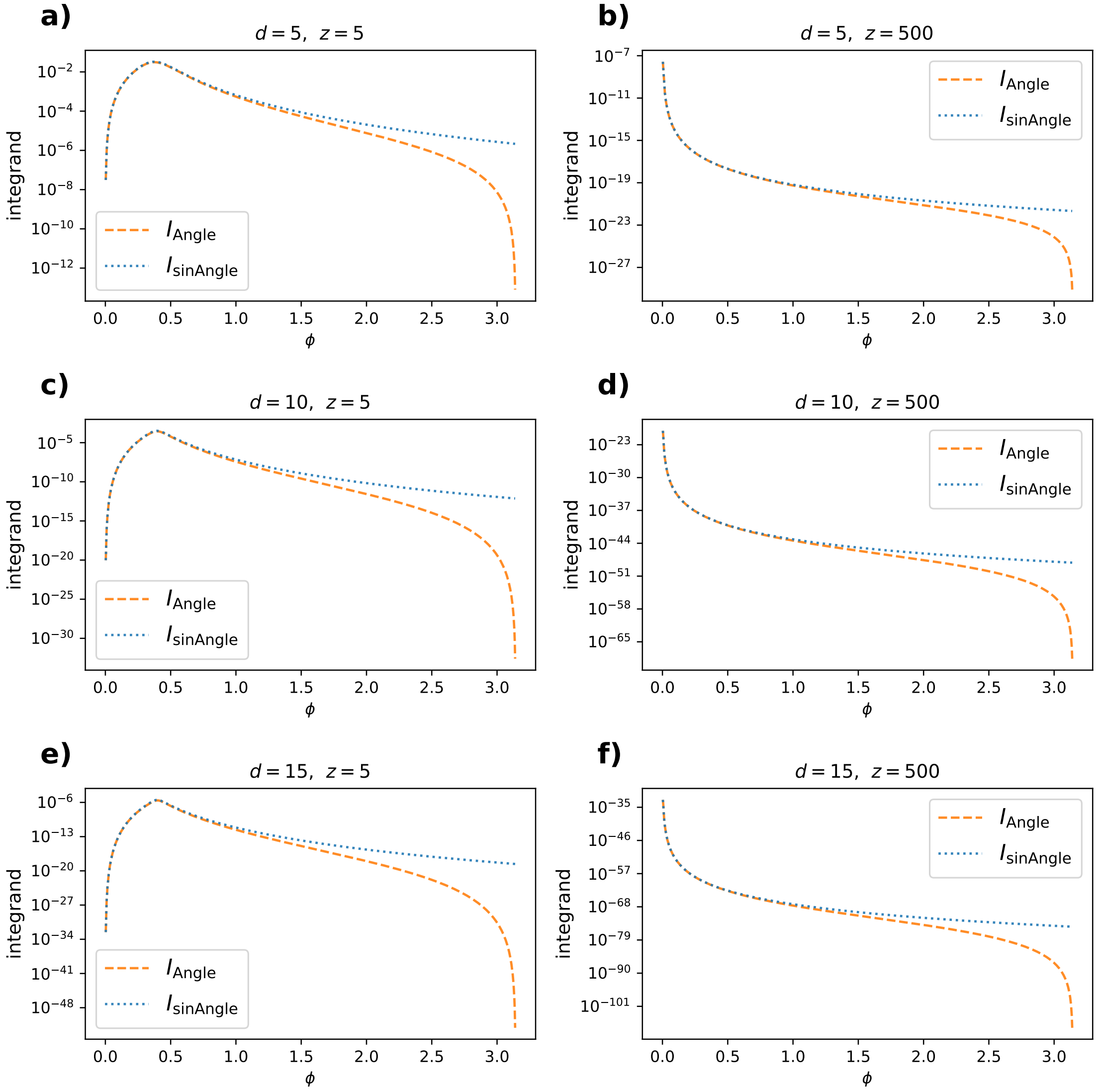

Figure S1.3.1. The effect of replacing the sinus functions by their argument in the integrand occurring at temperatures $T > 0$ in the probability that a link emerges between two nodes in a given connection attempt. The different panels were created using different number of dimensions d and different values of $z = e^{\frac{\zeta}{2}\cdot(r_{ij}+r_{jj}-R_j)}$, specified in the panel titles. The temperature T was set to $0.5/(d-1)$ in each case.

stay close to the expected curve even if we increase the number of dimensions d to 10 or 15 – not only at $T = 0.5/(d-1)$, but also at $T = 0$ and $T = 1.5/(d-1)$. This indicates that although the proper number of nodes is an increasing function of the number of dimensions d, in the case of large enough networks all of our approximations carried out in Sects. S1.1 and S1.2 work well even in hyperbolic spaces of relatively high number of dimensions. It is important to keep in mind, however, that the increase in the number of dimensions is accompanied by a decrease in both the average clustering coefficient of the dPSO networks of a given degree decay exponent and the modularity of their community structure, as indicated by Figs. 4 and 6 of the main text. Therefore, using extremely high-dimensional hyperbolic spaces for network generation may not be a reasonable thing to do in practical applications of the model.

S1.4 The role of the multiplying factor in the initial radial coordinates and the fPSO model

According to Refs. [1, 2], in the properly parametrised preferential attachment model where the connection probability of nodes i (appearing at time i) and j (appearing at time $j > i$) can be written as $\Pi(i,j) = m \cdot \frac{i^{-q}}{\int_1^j \ell^{-q}\mathrm{d}\ell}$ (as described in Eq. (S1.1)), the

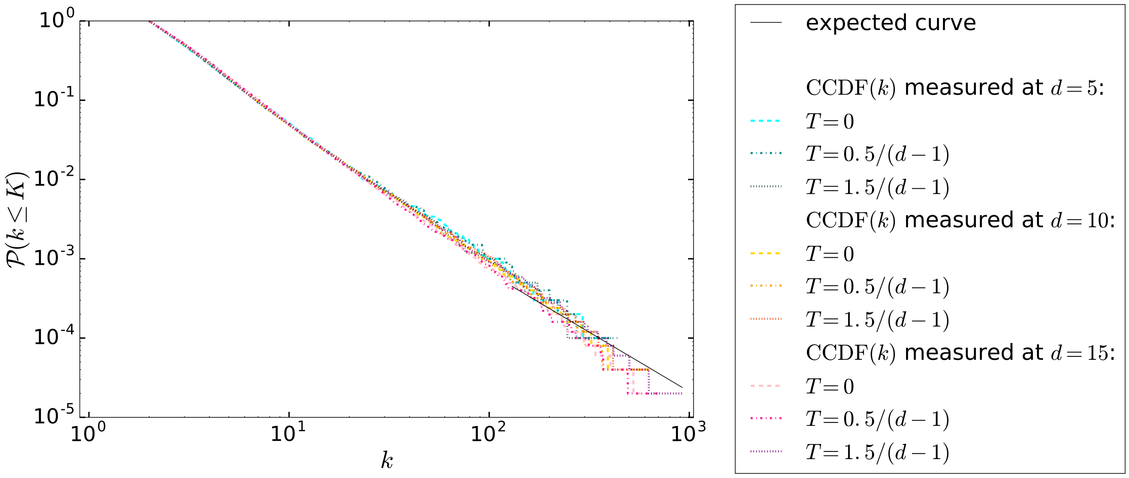

Figure S1.3.2. Complementary cumulative distribution function (CCDF) of the node degrees in networks generated by the dPSO model in higher-dimensional spaces. Each color palette corresponds to a given number of dimensions d, while each line style corresponds to a given temperature T. The number of nodes N was $10,000$ at $d=5$, $25,000$ at $d=10$ and $50,000$ at $d=15$. The expected degree decay exponent was set to $\gamma = 2.5$ in each case by using the popularity fading parameter $\beta = \frac{1}{(d-1)\cdot(2.5-1)}$. One network was generated in each case. The curvature of the hyperbolic space and the half of the expected average degree were the same for all networks, namely $K = -\zeta^2 = -1$ and $m = 2$. All the indicated simulation results match well the curve $\mathcal{P}(k \leq K) \sim k^{-(\gamma-1)}$ (shown by the solid line) that was expected based on the analytical calculations.

degree decay exponent takes the form of $\gamma = 1 + \frac{1}{q}$. The results of the previous sections show that in the dPSO model

$$\Pi(i,j) = \begin{cases} m \cdot \dfrac{e^{-\frac{\zeta\cdot(d-1)}{2}\cdot\beta\cdot r_{ii}}}{\int_1^j e^{-\frac{\zeta\cdot(d-1)}{2}\cdot\beta\cdot r_{\ell\ell}}\,\mathrm{d}\ell} & \text{if } 0 \leq T < \frac{1}{d-1}, \\ m \cdot \dfrac{e^{-\frac{\zeta}{2\cdot T}\cdot\beta\cdot r_{ii}}}{\int_1^j e^{-\frac{\zeta}{2\cdot T}\cdot\beta\cdot r_{\ell\ell}}\,\mathrm{d}\ell} & \text{if } \frac{1}{d-1} < T. \end{cases} \tag{S1.4.1}$$

Thus, setting the initial radial coordinate of each node $\ell \geq 1$ to $r_{\ell\ell} = f \cdot \ln \ell$ yields

$$\Pi(i,j) = \begin{cases} m \cdot \dfrac{i^{-f\cdot\frac{\zeta\cdot(d-1)}{2}\cdot\beta}}{\int_1^j \ell^{-f\cdot\frac{\zeta\cdot(d-1)}{2}\cdot\beta}\,\mathrm{d}\ell} & \text{if } 0 \leq T < \frac{1}{d-1}, \\ m \cdot \dfrac{i^{-f\cdot\frac{\zeta}{2\cdot T}\cdot\beta}}{\int_1^j \ell^{-f\cdot\frac{\zeta}{2\cdot T}\cdot\beta}\,\mathrm{d}\ell} & \text{if } \frac{1}{d-1} < T, \end{cases} \tag{S1.4.2}$$

i.e.,

$$\gamma = \begin{cases} 1 + \dfrac{1}{f\cdot\frac{\zeta\cdot(d-1)}{2}\cdot\beta} & \text{if } 0 \leq T < \frac{1}{d-1}, \\ 1 + \dfrac{1}{f\cdot\frac{\zeta}{2T}\cdot\beta} & \text{if } \frac{1}{d-1} < T. \end{cases} \tag{S1.4.3}$$

Since at $T < 1/(d-1)$ we used for any dimension d the same formula

$$r_{\ell\ell} = \frac{2}{\zeta} \cdot \ln \ell \tag{S1.4.4}$$

that was introduced in the original, two-dimensional PSO model [1], below the critical temperature $T_c = 1/(d-1)$ the degree decay exponent became dependent on the number of dimensions d besides the popularity fading parameter β and, at the same

time, independent of all the other model parameters ($\gamma = 1 + \frac{1}{(d-1)\cdot\beta}$). Then, in order to ensure that the formula of the degree decay exponent remains the same above the critical temperature (just as in the original PSO model), we defined the initial radial coordinates as

$$r_{\ell\ell} = \frac{2T(d-1)}{\zeta} \cdot \ln \ell \tag{S1.4.5}$$

at temperatures $1/(d-1) < T$. Note that using the same initial radial coordinates as in the $T < T_\text{c}$ case would yield $\gamma = 1 + T/\beta$ above the critical temperature.

Nevertheless, by redefining $r_{\ell\ell}$ as

$$r_{\ell\ell} = \begin{cases} \frac{2}{\zeta\cdot(d-1)} \cdot \ln \ell & \text{if } 0 \leq T < \frac{1}{d-1}, \\ \frac{2T}{\zeta} \cdot \ln \ell & \text{if } \frac{1}{d-1} < T, \end{cases} \tag{S1.4.6}$$

we can obtain a degree decay exponent formula that coincides with the well-known expression $\gamma = 1 + 1/\beta$ of the two-dimensional PSO model [1] for any number of dimensions, and we again recover the original radial coordinate formulas

$$r_{\ell\ell} = \begin{cases} \frac{2}{\zeta} \cdot \ln \ell & \text{if } 0 \leq T < \frac{1}{d-1}, \\ \frac{2T}{\zeta} \cdot \ln \ell & \text{if } \frac{1}{d-1} < T \end{cases} \tag{S1.4.7}$$

for $d = 2$. However, Eq. (S1.4.6) does not provide the possibility to achieve a degree decay exponent below 2, since in this case the smallest possible value of γ (obtained at $\beta = 1$) is

$$\gamma_{\min}(d) = \min\left(1 + \frac{1}{\beta}\right) = 2, \tag{S1.4.8}$$

independently of the number of dimensions. In contrast, if the initial radial coordinates are defined by Eqs. (S1.4.4) and (S1.4.5), then by increasing the number d of dimensions, the lower limit of the degree decay exponent can be decreased as

$$\gamma_{\min}(d) = \min\left(1 + \frac{1}{(d-1)\cdot\beta}\right) = 1 + \frac{1}{(d-1)}. \tag{S1.4.9}$$

With respect to the clustering and the community structure, the behaviour of the above-highlighted two variations of the dPSO model (differing in the initial radial coordinates given by either Eqs. (S1.4.4) and (S1.4.5) or Eq. (S1.4.6)) is the same, since for given values of ζ, d, N, m, γ and T, all the connection probabilities emerging during the network growth are equal. Looking at the approximating formula of the hyperbolic distance [3] written up for a new node j and a previously appeared node i as

$$x_{ij}(j) \approx r_{ij} + r_{jj} + \frac{2}{\zeta} \cdot \ln\left(\sin\left(\frac{\theta_{ij}}{2}\right)\right) = \beta \cdot r_{ii} + (1-\beta) \cdot r_{jj} + r_{jj} + \frac{2}{\zeta} \cdot \ln\left(\sin\left(\frac{\theta_{ij}}{2}\right)\right), \tag{S1.4.10}$$

one can observe that the differences in the attractiveness of the already existing nodes arise from the term $\beta \cdot r_{ii}$ and from the angular term. However, in a network characterised by a degree decay exponent γ, the term $\beta \cdot r_{ii}$ is equal to $\frac{2}{\zeta\cdot(d-1)\cdot(\gamma-1)} \cdot \ln i$ for both model variants. Thus, the difference in the initial radial coordinate formulas appears only in the term $(2-\beta) \cdot r_{jj}$ that is independent of i. And since the cutoff distance R_j is always set to that value at which the expected number of connections will be m, together with the equal change of the (radial) distances between the new node and all of its possible neighbours, the connection probability function

$$p(x_{ij}) = \frac{1}{1 + e^{\frac{\zeta(x_{ij} - R_j)}{2T}}} \tag{S1.4.11}$$

becomes shifted as well, leading to the emergence of a connection between node j and any previous node i eventually with the same probability in the case of using Eq. (S1.4.6) as in the case of defining the initial radial coordinates by Eqs. (S1.4.4) and (S1.4.5).

According to the above, in the $2 \leq \gamma$ regime these two model variants are equivalent. Consequently, although both definition of the initial radial coordinates are applicable, because of the wider range of achievable degree decay exponents, we stick to using Eqs. (S1.4.4) and (S1.4.5).

Let us now take a look at the effects of using the most general choice of the initial radial coordinates $r_{\ell\ell} = f \cdot \ln \ell$ in the two-dimensional case, which we will refer to as the fPSO model. According to Eq. (S1.4.3), the initial radial coordinates $r_{\ell\ell} = f \cdot \ln \ell$ with $2/(\zeta \cdot (d-1)) < f \cdot \beta$ for $T < 1/(d-1)$ and $2T/\zeta < f \cdot \beta$ for $1/(d-1) < T$ yield $\gamma < 2$ even in the two-dimensional hyperbolic space (i.e., at $d = 2$). Note that γ is a decreasing function of the multiplying factor f, meaning that the f factors that yield relatively small degree decay exponents are high enough to ensure that in the hyperbolic law of cosines

$$\cosh(\zeta x_{ij}(j)) = \cosh(\zeta r_{ij})\cosh(\zeta r_{jj}) - \sinh(\zeta r_{ij})\sinh(\zeta r_{jj})\cos(\theta_{ij}) \tag{S1.4.12}$$

the terms ζr_{ij} and ζr_{jj} are sufficiently large and the hyperbolic distance can be written for most of the node pairs as

$$x_{ij}(j) \approx r_{ij} + r_{jj} + \frac{2}{\zeta} \cdot \ln\left(\sin\left(\frac{\theta_{ij}}{2}\right)\right), \tag{S1.4.13}$$

which is an essential approximation in the derivation of the scale-free degree distribution. Nevertheless, to obtain higher values of γ, it is better to decrease only the popularity fading parameter β and not the multiplying factor f, since otherwise the approximation in Eq. (S1.4.13) becomes invalid due to the smallness of the radial coordinates.

To generate networks on the hyperbolic plane with initial radial coordinates $r_{\ell\ell} = f \cdot \ln \ell$, the cutoff distance R_j has to be calculated for $T \neq 0$. According to Eqs. (S1.2.10) and (S1.2.11), for $d = 2$

$$P(i,j) \approx \begin{cases} \frac{2 \cdot T}{(j-1)\cdot\sin(T\cdot\pi)} \cdot e^{-\frac{\zeta}{2}\cdot(r_{ij}+r_{jj}-R_j)} & \text{if } T < \frac{1}{d-1}, \\ \frac{1}{(j-1)\cdot\pi} \cdot \int_0^{\pi} \frac{1}{(\phi/2)^{1/T}} \,\mathrm{d}\phi \cdot e^{-\frac{\zeta}{2T}\cdot(r_{ij}+r_{jj}-R_j)} = \frac{2^{1/T}\cdot T}{(j-1)\cdot\pi^{1/T}\cdot(T-1)} \cdot e^{-\frac{\zeta}{2T}\cdot(r_{ij}+r_{jj}-R_j)} & \text{if } \frac{1}{d-1} < T. \end{cases} \tag{S1.4.14}$$

Note that here, in accordance with Ref. [1], $\sin(\phi/2)$ was approximated with $\phi/2$ above the critical temperature $T_c = 1/(d-1)$ too. Using the initial radial coordinate formula $r_{\ell\ell} = f \cdot \ln \ell$ and that $r_{ij} = \beta \cdot r_{ii} + (1-\beta) \cdot r_{jj}$, we arrive at

$$P(i,j) \approx \begin{cases} \frac{2\cdot T}{(j-1)\cdot\sin(T\cdot\pi)} \cdot i^{-\frac{\zeta\cdot f\cdot\beta}{2}} \cdot j^{-\frac{\zeta\cdot f\cdot(2-\beta)}{2}} \cdot e^{\frac{\zeta}{2}\cdot R_j} & \text{if } T < \frac{1}{d-1}, \\ \frac{2^{1/T}\cdot T}{(j-1)\cdot\pi^{1/T}\cdot(T-1)} \cdot i^{-\frac{\zeta\cdot f\cdot\beta}{2T}} \cdot j^{-\frac{\zeta\cdot f\cdot(2-\beta)}{2T}} \cdot e^{\frac{\zeta}{2T}\cdot R_j} & \text{if } \frac{1}{d-1} < T. \end{cases} \tag{S1.4.15}$$

The cutoff distance R_j of the connection probability at the appearance of node j can be expressed from the equation

$$m = (j-1)\cdot\int_1^j P(i,j)\,\mathrm{d}i = \begin{cases} \frac{2\cdot T}{\sin(T\cdot\pi)} \cdot \frac{j^{1-\zeta\cdot f\cdot\beta/2}-1}{1-\zeta\cdot f\cdot\beta/2} \cdot j^{-\frac{\zeta\cdot f\cdot(2-\beta)}{2}} \cdot e^{\frac{\zeta}{2}\cdot R_j} = \frac{2\cdot T}{\sin(T\cdot\pi)} \cdot \frac{j^{1-\zeta\cdot f} - j^{\zeta\cdot f\cdot(\beta-2)/2}}{1-\zeta\cdot f\cdot\beta/2} \cdot e^{\frac{\zeta}{2}\cdot R_j} & \text{if } T < \frac{1}{d-1}, \\ \frac{2^{1/T}\cdot T}{\pi^{1/T}\cdot(T-1)} \cdot \frac{j^{1-\zeta\cdot f\cdot\beta/(2T)}-1}{1-\zeta\cdot f\cdot\beta/(2T)} \cdot j^{-\frac{\zeta\cdot f\cdot(2-\beta)}{2T}} \cdot e^{\frac{\zeta}{2T}\cdot R_j} = \frac{2^{1/T}\cdot T}{\pi^{1/T}\cdot(T-1)} \cdot \frac{j^{1-\zeta\cdot f/T} - j^{\zeta\cdot f\cdot(\beta-2)/(2T)}}{1-\zeta\cdot f\cdot\beta/(2T)} \cdot e^{\frac{\zeta}{2T}\cdot R_j} & \text{if } \frac{1}{d-1} < T \end{cases} \tag{S1.4.16}$$

as

$$R_j = \begin{cases} \frac{2}{\zeta} \cdot \ln\left(\frac{m\cdot\sin(T\cdot\pi)\cdot(1-\zeta\cdot f\cdot\beta/2)}{2\cdot T\cdot\left(j^{1-\zeta\cdot f} - j^{\zeta\cdot f\cdot(\beta-2)/2}\right)}\right) & \text{if } T < \frac{1}{d-1}, \\ \frac{2T}{\zeta} \cdot \ln\left(\frac{m\cdot\pi^{1/T}\cdot(T-1)\cdot(1-\zeta\cdot f\cdot\beta/(2T))}{2^{1/T}\cdot T\cdot\left(j^{1-\zeta\cdot f/T} - j^{\zeta\cdot f\cdot(\beta-2)/(2T)}\right)}\right) & \text{if } \frac{1}{d-1} < T. \end{cases} \tag{S1.4.17}$$

Our implementation of this two-dimensional fPSO model is available from Ref. [7].

First, in Fig. S1.4.1, we show examples for fPSO network layouts generated in the native representation of the hyperbolic plane. Then, Fig. S1.4.2 compares the behaviour of the fPSO model and the dPSO model with regard to the degree distribution. Finally, Figs. S1.4.3 and S1.4.4 present the changes in the average clustering coefficient $\bar{c}$ and the modularity Q [8, 9] of the community structure detected by the Louvain algorithm [10, 11] in fPSO and dPSO networks as a function of the temperature T and the degree decay exponent γ. According to these plots, in that parameter regime where the degree distribution of the fPSO networks is well-described by the theory built on the approximating formula of the hyperbolic distance given by Eq. (S1.4.13), i.e. when the multiplying factor f of the initial radial coordinates is large enough, there is no substantial difference between a

two-dimensional fPSO network and a dPSO network of the same degree decay exponent and the possible lowest number of dimensions with regard to the strength of the clustering and the community structure. However, while it is easy to determine the lowest d at which a given small degree decay exponent γ is achievable ($d_{\min} = \lceil 1 + \frac{1}{\beta_{\max}\cdot(\gamma-1)} \rceil = \lceil 1 + \frac{1}{\gamma-1} \rceil$), it is rather burdensome to determine the exact limit of the factor f that separates the regions in which the hyperbolic distance is well or poorly approximable in practice. Therefore, instead of using the two-dimensional fPSO model and adjusting the $\beta - f$ parameter pair, we prefer the dPSO model where γ can be controlled via the $\beta - d$ setting.

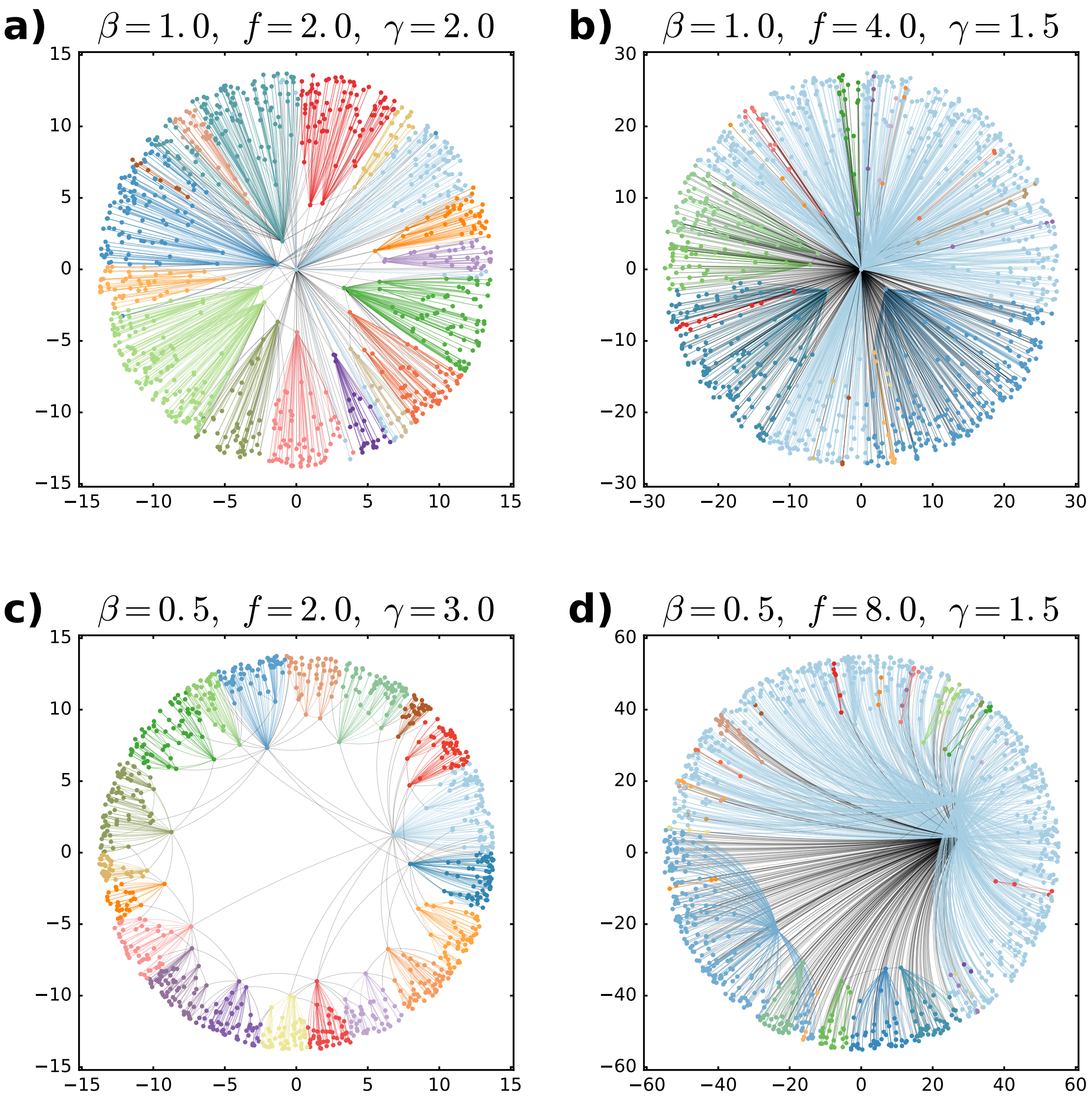

Figure S1.4.1. Native layouts of networks generated by the fPSO model on the hyperbolic plane. The curvature of the hyperbolic plane, the number of nodes, the half of the expected average degree and the temperature were the same for all networks, namely $K = -\zeta^2 = -1$, $N = 1000$, $m = 2$ and $T = 0$. Since $T < 1/(d-1) = 1$, the degree decay exponent γ can be expressed with the popularity fading parameter β and the multiplying factor f of the initial radial coordinates as $\gamma = 1 + \frac{2}{f\cdot\zeta\cdot(d-1)\cdot\beta}$ (see Eq. (S1.4.3)). Note that for the here-applied $d = 2$ and $\zeta = 1$ settings, $f = 2$ gives back the original PSO model. The colouring of the nodes and the links indicates communities found by the Louvain algorithm.

S1.4.1 Formulas of the radial coordinates and the popularity fading parameter in the case of d-dimensional embeddings

As it is described above, there is more than one possible choice regarding the multiplying factor of the initial radial coordinates in the extension of the two-dimensional PSO model to any integer number of dimensions $d \geq 2$. Although of the detailed two

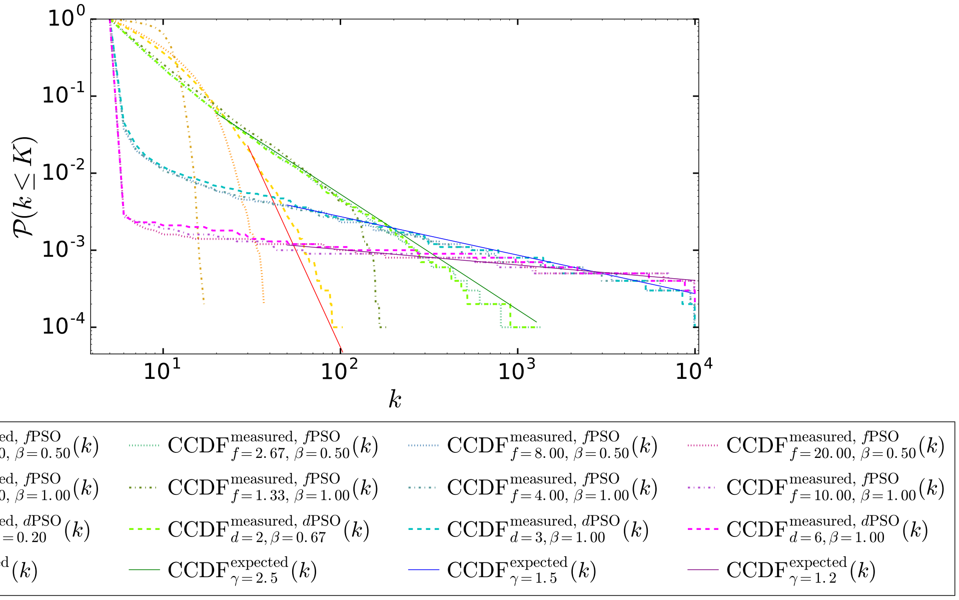

Figure S1.4.2. Complementary cumulative distribution function (CCDF) of the node degrees for networks generated by the fPSO model and the dPSO model. We examined two degree decay exponents that can not be obtained from the original PSO model: $\gamma = 1.5$ and $\gamma = 1.2$. Both of them are achievable with several $\beta - f$ settings in the two-dimensional fPSO model (where $\gamma = 1 + \frac{2}{f \cdot \zeta \cdot \beta}$ at $T < 1$), just as with the dPSO model of high enough number of dimensions d (where $\gamma = 1 + \frac{1}{(d-1)\cdot\beta}$ at $T < \frac{1}{d-1}$). However, with the decrease of the multiplying factor f of the initial radial coordinates in the fPSO model, the approximation of the hyperbolic distance formula given by Eq. (S1.4.13) becomes invalid. As a result, the fPSO model of $\beta = 1$ already failed to generate the degree distribution characterised by the exponent $\gamma = 2.5$, and both the $\beta = 1$ and the $\beta = 0.5$ settings of the fPSO model yielded considerable deviations from the expected curve of $\gamma = 6$, while the degree distribution of the original PSO model (corresponding to the dPSO model with $d = 2$ or the two-dimensional fPSO model with $f = 2/\zeta$) obtained at $\beta = 1/(\gamma - 1)$ behaves as expected even for these higher values of γ. The curvature of the hyperbolic space, the number of nodes, the half of the expected average degree and the temperature were the same for all networks, namely $K = -\zeta^2 = -1$ (using $\zeta = 1$), $N = 10{,}000$, $m = 5$ and $T = 0$. One network was generated with each parameter setting.

approaches given by

$$r_{\ell\ell} = \begin{cases} \frac{2}{\zeta\cdot(d-1)} \cdot \ln \ell & \text{if } 0 \leq T < \frac{1}{d-1}, \\ \frac{2T}{\zeta} \cdot \ln \ell & \text{if } \frac{1}{d-1} < T \end{cases} \tag{S1.4.1.1}$$

and

$$r_{\ell\ell} = \begin{cases} \frac{2}{\zeta} \cdot \ln \ell & \text{if } 0 \leq T < \frac{1}{d-1}, \\ \frac{2T(d-1)}{\zeta} \cdot \ln \ell & \text{if } \frac{1}{d-1} < T \end{cases} \tag{S1.4.1.2}$$

only the latter can be used to obtain degree decay exponents below 2, both of these approaches may be suitable for the generation, and thus also the hyperbolic embedding of networks with $2 \leq \gamma$. The two model variants provide two different ways for the radial arrangement of a network with size N and degree decay exponent γ in a d-dimensional hyperbolic space of curvature $K = -\zeta^2$. In both cases, unless the average clustering coefficient of the network to be embedded is very close to 0, it can be assumed that the temperature T that corresponds to the network is smaller than the critical value $T_c = 1/(d-1)$, and therefore the radial coordinate formulas of the $0 \leq T < 1/(d-1)$ case can be used. Accordingly, if the popularity fading

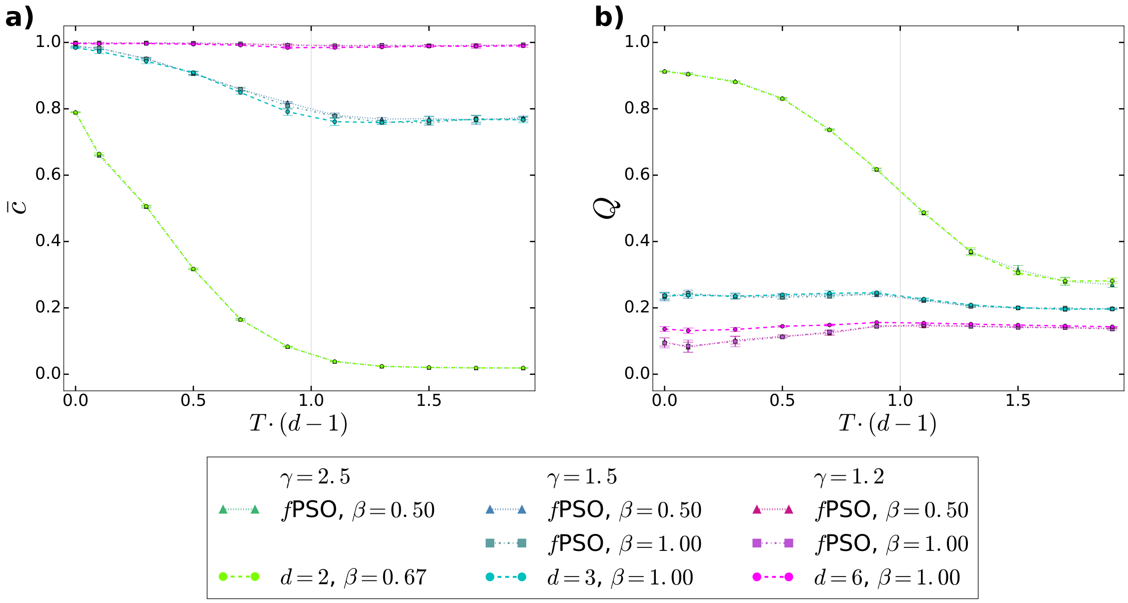

Figure S1.4.3. Average clustering coefficient $\bar{c}$ and modularity Q of the community structure detected by the Louvain algorithm in networks generated by the two-dimensional fPSO model and the dPSO model as a function of the rescaled temperature $T\cdot(d-1)$. In order to create a fair comparison with the corresponding dPSO networks, for the fPSO model we show here only the results of such parameter settings that, according to Fig. S1.4.2, actually yield the expected degree distribution, i.e. where the multiplying factor f of the initial radial coordinates is not too small. Each displayed data point was obtained by averaging over 5 networks generated independently with a given set of model parameters, setting the curvature of the hyperbolic space to -1 (using $\zeta=1$), the number of nodes to $10,000$ and the half of the expected average degree to 5 in each case. The error bars show the standard deviations measured among the 5 networks. The grey vertical lines indicate the critical point $T_c=1/(d-1)$.

parameter β is determined as

$$\beta=\frac{1}{\gamma-1}, \tag{S1.4.1.3}$$

then the radial coordinate of the node having the ℓth ($\ell=1,2,...,N$) largest degree (with ties in the order of node degrees broken arbitrarily) can be formulated as

$$r_{\ell N}=\beta\cdot r_{\ell\ell}+(1-\beta)\cdot r_{NN}=\beta\cdot\frac{2}{\zeta\cdot(d-1)}\cdot\ln\ell+(1-\beta)\cdot\frac{2}{\zeta\cdot(d-1)}\cdot\ln N, \tag{S1.4.1.4}$$

while if the formula

$$\beta=\frac{1}{(d-1)\cdot(\gamma-1)} \tag{S1.4.1.5}$$

is used, then the radial coordinate in question can be calculated as

$$r_{\ell N}=\beta\cdot r_{\ell\ell}+(1-\beta)\cdot r_{NN}=\beta\cdot\frac{2}{\zeta}\cdot\ln\ell+(1-\beta)\cdot\frac{2}{\zeta}\cdot\ln N. \tag{S1.4.1.6}$$

Note that angular coordinates can be assigned to the network nodes independently from the radial positions using e.g. a method proposed in Ref. [12].

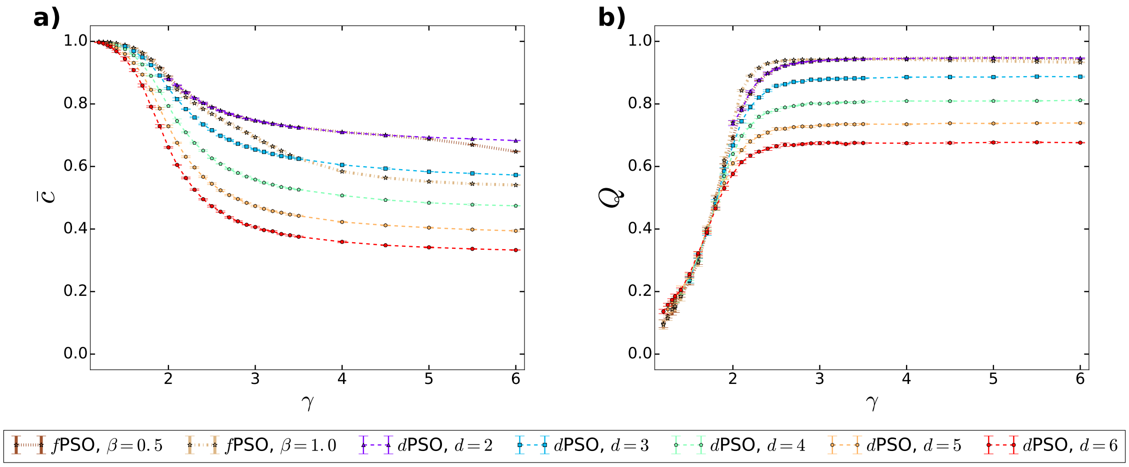

Figure S1.4.4. Average clustering coefficient $\bar{c}$ and modularity Q of the community structure detected by the Louvain algorithm in networks generated by the two-dimensional fPSO model and the dPSO model as a function of the degree decay exponent γ. The depicted data points correspond to the values averaged over 5 networks generated with the same parameter settings, while the error bars show the standard deviations measured among the 5 networks. The curvature of the hyperbolic space, the number of nodes, the half of the expected average degree and the temperature were the same for all networks, namely $K = -\zeta^2 = -1$, $N = 10,000$, $m = 5$ and $T = 0$. Note that for the fPSO model of a given popularity fading parameter β, the increase in the expected degree decay exponent γ corresponds to a decrease in the multiplying factor f of the initial radial coordinates, which ruins the approximation in Eq. (S1.4.13) and hereby increases the deviation between the degree distribution of the fPSO networks and the dPSO networks of the same expected degree decay exponent.

S2 Cutoff distance of the connection probability in the dPSO model

The cutoff distance R_j of the connection probability $p(x) = 1/[1+e^{\zeta(x-R_j)/(2T)}]$ applied at time j is set to the value ensuring that the expected number of nodes connecting to node j at its arrival is equal to m. As derived in Sect. S1.1, for $T = 0$ the cutoff distance can be calculated as

$$R_j = \frac{2}{\zeta\cdot(d-1)}\cdot\ln\left(\frac{(d-1)\cdot m}{\eta(d)\cdot 2^{d-1}\cdot e^{-\frac{\zeta\cdot(d-1)}{2}\cdot(2-\beta)\cdot r_{jj}}\cdot\int_1^j e^{-\frac{\zeta\cdot(d-1)}{2}\cdot\beta\cdot r_{ii}}\,\mathrm{d}i}\right), \tag{S2.1}$$

or, using that $r_{\ell\ell} = (2/\zeta)\cdot\ln\ell$, as

$$R_j = \frac{2}{\zeta\cdot(d-1)}\cdot\ln\left(\frac{(d-1)\cdot m}{\eta(d)\cdot 2^{d-1}\cdot j^{-(d-1)\cdot(2-\beta)}\cdot\int_1^j i^{-(d-1)\cdot\beta}\,\mathrm{d}i}\right) = \frac{2}{\zeta\cdot(d-1)}\cdot\ln\left(\frac{(d-1)\cdot m}{\eta(d)\cdot 2^{d-1}\cdot j^{-(d-1)\cdot(2-\beta)}\cdot\frac{j^{1-(d-1)\cdot\beta}-1}{1-(d-1)\cdot\beta}}\right) =$$
$$= \frac{2}{\zeta\cdot(d-1)}\cdot\ln\left(\frac{(d-1)\cdot m\cdot(1-(d-1)\cdot\beta)}{\eta(d)\cdot 2^{d-1}\cdot\left(j^{3-2\cdot d}-j^{(d-1)\cdot(\beta-2)}\right)}\right). \tag{S2.2}$$

Note that for $d = 2$ this formula is the same as the result

$$R_j = r_{jj} - \frac{2}{\zeta}\cdot\ln\left(\frac{2\cdot\left(1-e^{-\frac{\zeta}{2}(1-\beta)\cdot r_{jj}}\right)}{\pi\cdot m\cdot(1-\beta)}\right) \tag{S2.3}$$

of Ref. [1], which can be written as

$$R_j = \frac{2}{\zeta}\cdot\ln\left(\frac{\pi\cdot m\cdot(1-\beta)}{2\cdot\left(j^{-1}-j^{\beta-2}\right)}\right) \tag{S2.4}$$

after the substitution of $r_{jj} = (2/\zeta)\cdot\ln j$.

In the case of $0 < T$, the cutoff distance at time j is defined by the equation

$$m = (j-1)\cdot P(j), \tag{S2.5}$$

where $j-1$ is the number of already existing nodes at the appearance of node j and $P(j)$ is the probability that node j connects to any of the previously appeared nodes in a given link formation attempt. According to Sect. S1.2,

$$P(j) = \int_1^j P(i,j)\,\mathrm{d}i = \int_1^j \frac{\eta(d)}{j-1}\cdot\int_0^\pi \frac{\sin^{d-2}\phi}{1+\left(e^{\frac{\zeta}{2}\cdot(r_{ij}+r_{jj}-R_j)}\cdot\sin\left(\frac{\phi}{2}\right)\right)^{\frac{1}{T}}}\,\mathrm{d}\phi\,\mathrm{d}i \tag{S2.6}$$

with

$$\eta(d) = \begin{cases} \frac{\left(\frac{d}{2}-1\right)!\cdot\frac{d-2}{2}!\cdot 2^{d-2}}{(d-2)!\cdot\pi} & \text{if } d \text{ is even,} \\ \frac{(d-1)!}{\left(\frac{d-1}{2}-1\right)!\cdot\frac{d-1}{2}!\cdot 2^{d-1}} & \text{if } d \text{ is odd.} \end{cases} \tag{S2.7}$$

Knowing also that the radial coordinate of node ℓ at time j is

$$r_{\ell j} = \beta\cdot r_{\ell\ell} + (1-\beta)\cdot r_{jj} = \begin{cases} \beta\cdot\frac{2}{\zeta}\cdot\ln\ell + (1-\beta)\cdot\frac{2}{\zeta}\cdot\ln j & \text{if } 0 \le T < \frac{1}{d-1}, \\ \beta\cdot\frac{2T(d-1)}{\zeta}\cdot\ln\ell + (1-\beta)\cdot\frac{2T(d-1)}{\zeta}\cdot\ln j & \text{if } \frac{1}{d-1} < T, \end{cases} \tag{S2.8}$$

we arrive at the equation

$$m = \begin{cases} \eta(d)\cdot\int_1^j\int_0^\pi \frac{\sin^{d-2}\phi}{1+\sin^{\frac{1}{T}}(\phi/2)\cdot i^{\frac{\beta}{T}}\cdot j^{\frac{2-\beta}{T}}\cdot e^{-\frac{\zeta}{2T}\cdot R_j}}\,\mathrm{d}\phi\,\mathrm{d}i & \text{if } 0 < T < \frac{1}{d-1}, \\ \eta(d)\cdot\int_1^j\int_0^\pi \frac{\sin^{d-2}\phi}{1+\sin^{\frac{1}{T}}(\phi/2)\cdot i^{\beta\cdot(d-1)}\cdot j^{(2-\beta)\cdot(d-1)}\cdot e^{-\frac{\zeta}{2T}\cdot R_j}}\,\mathrm{d}\phi\,\mathrm{d}i & \text{if } \frac{1}{d-1} < T, \end{cases} \tag{S2.9}$$

which can be solved numerically to obtain the cutoff distance R_j.

However, in the case of sufficiently large networks, for most of the nodes formula S2.6 can be approximated as

$$P(j) \approx \begin{cases} \frac{\eta(d)\cdot\pi\cdot 2^{d-1}\cdot T}{(j-1)\cdot\sin((d-1)\cdot T\cdot\pi)}\cdot\frac{j^{3-2\cdot d}-j^{(d-1)\cdot(\beta-2)}}{1-(d-1)\cdot\beta}\cdot e^{\frac{\zeta\cdot(d-1)}{2}\cdot R_j} & \text{if } 0<T<\frac{1}{d-1}, \\ \frac{\eta(d)}{j-1}\cdot\int_0^{\pi}\frac{\sin^{d-2}\phi}{\sin^{\frac{1}{T}}(\phi/2)}\mathrm{d}\phi\cdot\frac{j^{3-2\cdot d}-j^{(d-1)\cdot(\beta-2)}}{1-(d-1)\cdot\beta}\cdot e^{\frac{\zeta}{2\cdot T}\cdot R_j} & \text{if } \frac{1}{d-1}<T. \end{cases} \tag{S2.10}$$

Substituting this in Eq. (S2.5), after some rearrangement of the terms one can write up the cutoff distance of the connection probability at the appearance of node j as

$$R_j \approx \begin{cases} \frac{2}{\zeta\cdot(d-1)}\cdot\ln\left(\frac{m\cdot\sin((d-1)\cdot T\cdot\pi)\cdot(1-(d-1)\cdot\beta)}{\eta(d)\cdot\pi\cdot 2^{d-1}\cdot T\cdot\left(j^{3-2\cdot d}-j^{(d-1)\cdot(\beta-2)}\right)}\right) & \text{if } 0<T<\frac{1}{d-1}, \\ \frac{2\cdot T}{\zeta}\cdot\ln\left(\frac{m\cdot(1-(d-1)\cdot\beta)}{\eta(d)\cdot\int_0^{\pi}\sin^{d-2}(\phi)\cdot\sin^{-1/T}(\phi/2)\,\mathrm{d}\phi\cdot\left(j^{3-2\cdot d}-j^{(d-1)\cdot(\beta-2)}\right)}\right) & \text{if } \frac{1}{d-1}<T, \end{cases} \tag{S2.11}$$

where $\int_0^{\pi}\sin^{d-2}(\phi)\cdot\sin^{-1/T}(\phi/2)\,\mathrm{d}\phi$ can be calculated numerically. Note that since $\lim_{T\to 0}\sin((d-1)\cdot T\cdot\pi)/(T\cdot\pi) = = d-1$, for $T\to 0$ the approximated cutoff distance formula of the $0<T<1/(d-1)$ case becomes Eq. (S2.2) as expected. For $d=2$, the approximating formula of the $0<T<1/(d-1)$ case gives back

$$R_j = r_{jj}-\frac{2}{\zeta}\cdot\ln\left(\frac{2\cdot T\cdot\left(1-e^{-\frac{\zeta}{2}(1-\beta)\cdot r_{jj}}\right)}{\sin(T\cdot\pi)\cdot m\cdot(1-\beta)}\right) = \frac{2}{\zeta}\cdot\ln\left(\frac{m\cdot\sin(T\cdot\pi)\cdot(1-\beta)}{2\cdot T\cdot\left(j^{-1}-j^{\beta-2}\right)}\right) \tag{S2.12}$$

derived in Ref. [1] for $0<T<1$ using $r_{jj}=(2/\zeta)\cdot\ln j$. Furthermore, if one uses the approximation $\sin(\phi/2)\approx\phi/2$ also in the $1/(d-1)<T$ case as in Ref. [1], then Eq. (S2.11) yields for $d=2$ and $1<T$

$$R_j = \frac{2\cdot T}{\zeta}\cdot\ln\left(\frac{m\cdot(1-\beta)}{\frac{1}{\pi}\cdot 2^{1/T}\cdot\int_0^{\pi}\phi^{-1/T}\,\mathrm{d}\phi\cdot\left(j^{-1}-j^{\beta-2}\right)}\right), \tag{S2.13}$$

which corresponds to

$$R_j = r_{jj}-\frac{2\cdot T}{\zeta}\cdot\ln\left(\left(\frac{2}{\pi}\right)^{\frac{1}{T}}\cdot\frac{T}{T-1}\cdot\frac{1-e^{-\frac{\zeta}{2\cdot T}\cdot(1-\beta)\cdot r_{jj}}}{m\cdot(1-\beta)}\right) = \frac{2\cdot T}{\zeta}\cdot\ln\left(\frac{\pi^{1/T}\cdot(T-1)\cdot m\cdot(1-\beta)}{2^{1/T}\cdot T\cdot(j^{-1}-j^{\beta-2})}\right) \tag{S2.14}$$

derived in Ref. [1] for $1<T$ using $r_{jj}=(2T/\zeta)\cdot\ln j$.

Finally, it is important to clarify how Eqs. (S2.2) and (S2.11) behave in the $\beta\to 1/(d-1)$ limit. As

$$\lim_{\beta\to\frac{1}{d-1}}\frac{1-(d-1)\cdot\beta}{j^{3-2\cdot d}-j^{(d-1)\cdot(\beta-2)}} = \frac{j^{2\cdot d-3}}{\ln j}, \tag{S2.15}$$

in the $\beta=1/(d-1)$ case the cutoff distance takes the form of

$$R_j \approx \begin{cases} \frac{2}{\zeta\cdot(d-1)}\cdot\ln\left(\frac{(d-1)\cdot m\cdot j^{2\cdot d-3}}{\eta(d)\cdot 2^{d-1}\cdot\ln j}\right) & \text{if } T=0, \\ \frac{2}{\zeta\cdot(d-1)}\cdot\ln\left(\frac{m\cdot\sin((d-1)\cdot T\cdot\pi)\cdot j^{2\cdot d-3}}{\eta(d)\cdot\pi\cdot 2^{d-1}\cdot T\cdot\ln j}\right) & \text{if } 0<T<\frac{1}{d-1}, \\ \frac{2\cdot T}{\zeta}\cdot\ln\left(\frac{m\cdot j^{2\cdot d-3}}{\eta(d)\cdot\int_0^{\pi}\sin^{d-2}(\phi)\cdot\sin^{-1/T}(\phi/2)\,\mathrm{d}\phi\cdot\ln j}\right) & \text{if } \frac{1}{d-1}<T. \end{cases} \tag{S2.16}$$

In our simulations, in order to reduce the computational time, we always calculated the cutoff distances at $0<T$ based on the approximating formulas given by Eqs. (S2.11) and (S2.16) instead of solving numerically Eq. (S2.9). According to Fig. S2.1, the difference between the results of the approximating formulas and the numerical equation solution measured for the 1000th node is already acceptable at most of the examined parameter settings. Therefore, in the case of the studied networks of size $N=10,000$ we can assume for most of the network nodes that the approximated cutoff distance was close enough to the value that could have been obtained by solving numerically Eq. (S2.9).

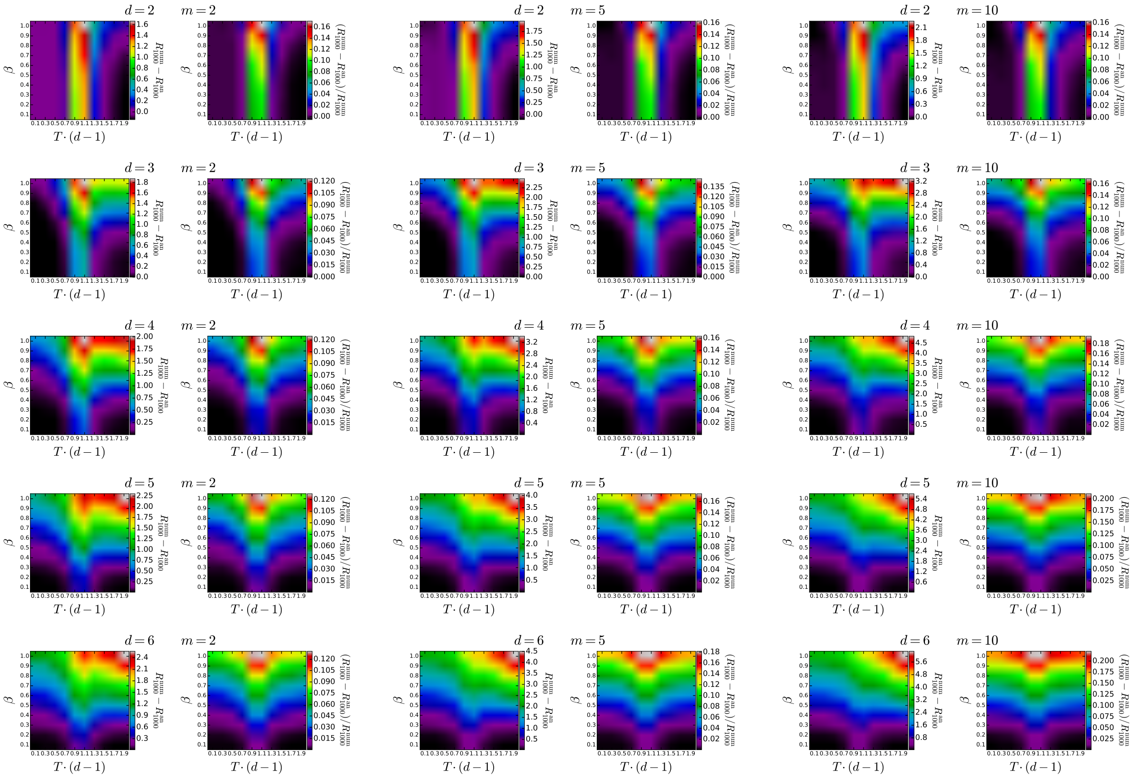

Figure S2.1. Absolute and relative difference between the results of the two methods proposed for calculating the cutoff distance of the connection probability at $0 < T$. We calculated the cutoff distance for the node that arrives at the 1000th time step using Eq. (S2.9), or Eqs. (S2.11) and (S2.16). The result of the first approach is denoted by R_{1000}^{num}, since it is based on the numerical solution of Eq. (S2.9), while R_{1000}^{an} stands for the cutoff distance obtained from the latter approach that is, at least for $T < 1/(d-1)$, an analytical calculation. Each pair of subplots depicts the effect of changing the popularity fading parameter β and the rescaled temperature $T \cdot (d-1)$, with the number of dimensions d and the half m of the expected average degree $\bar{k}$ given in the title of the subplot pair. The curvature K of the hyperbolic space was set to -1 in each case, i.e. we always used $\zeta = 1$.

S3 Simulation results for dPSO networks

This section presents simulation results additional to the figures of the main text. First, we confirm by Fig. S3.1 that in networks generated by the dPSO model of different expected average degree $2m$, the tail of the complementary cumulative distribution function (CCDF) of the node degrees follows a power-law written as $\mathcal{P}(k \leq K) \sim k^{-(\gamma-1)}$, where the degree decay exponent γ can be expressed with the dimension d of the hyperbolic space and the popularity fading parameter β as $1+\frac{1}{(d-1)\cdot\beta}$. As it is demonstrated by Fig. 2 of the main text, the temperature T does not have any substantial impact on the degree distribution; therefore, here we study only the $T=0$ case.

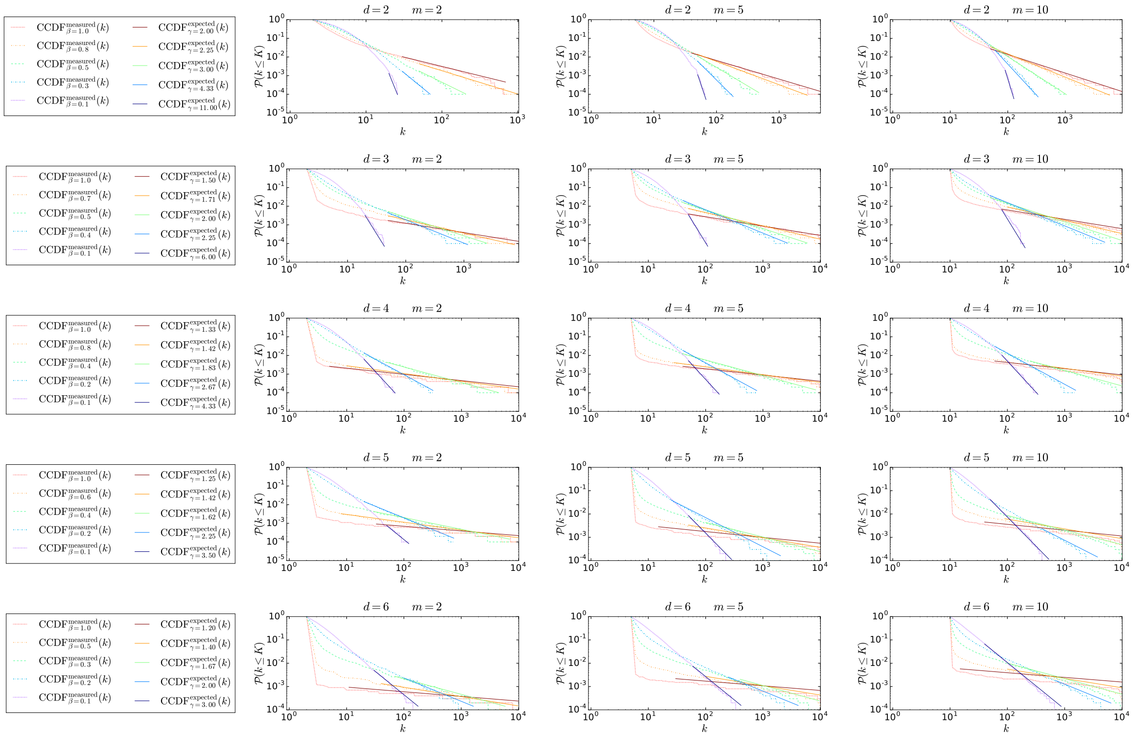

Figure S3.1. Complementary cumulative distribution function (CCDF) of the node degrees for networks generated by the dPSO model using different parametrisations. Each row of panels was created using a given dimension d, while each column of subplots presents the results obtained with a given value of m, as indicated in the title of the subplots. The popularity fading parameters and the corresponding degree decay exponents tested at each value of m for a given dimension d are listed in the leftmost panel of each row. The curvature of the hyperbolic space, the number of nodes and the temperature were the same for all networks, namely $K=-\zeta^2=-1$, $N=10,000$ and $T=0$. One network was generated with each of the parameter sets.

Next, to supplement Fig. 3 of the main text, in Fig. S3.2 we depict on a 10×10 grid in the $T - \beta$ parameter plane the average clustering coefficient $\bar{c}$ measured in dPSO networks of different number of dimensions d and expected average degree $2m$. Similarly, we add more detail to Fig. 5 of the main text by Figs. S3.3, S3.5 and S3.7, where we present for several different parameter settings the modularity Q (described in Sect. 3.3 of the main text) achieved by each of the examined community detection algorithms, namely Louvain [10, 11], Infomap [13, 14] and asynchronous label propagation [15, 16]. In Figs. S3.4, S3.6 and S3.8 we also plot the average and the standard deviation of the size of the communities found by the given algorithms.

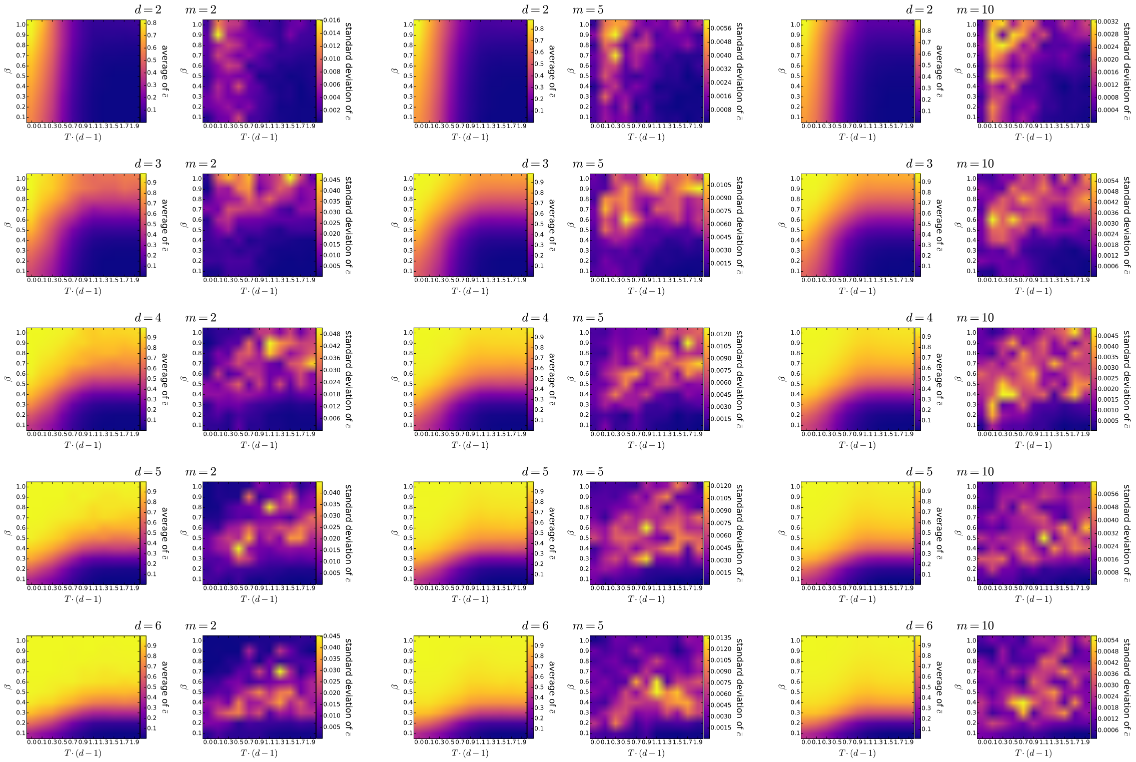

Figure S3.2. The mean and the standard deviation of the average clustering coefficient $\bar{c}$ measured in 5 dPSO networks in the case of different parametrisations. Each pair of subplots depicts the effect of changing the popularity fading parameter β and the rescaled temperature $T \cdot (d-1)$, with the number of dimensions d and the half m of the expected average degree $\bar{k}$ given in the title of the subplot pair. The number of nodes was $N = 10,000$ in each network. The curvature K of the hyperbolic space was always set to -1, i.e. we used $\zeta = 1$.

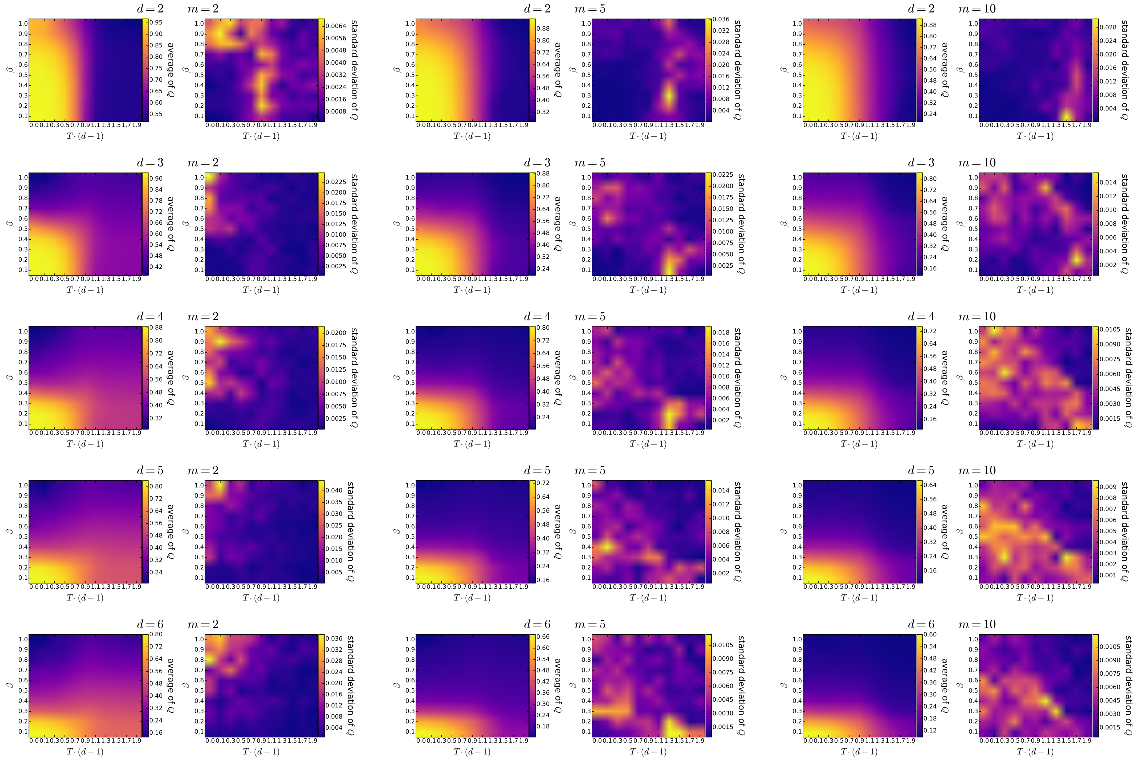

Figure S3.3. The mean and the standard deviation of the modularity Q of the community structure detected by the *Louvain* algorithm in 5 *d*PSO networks in the case of different parametrisations. Each pair of subplots depicts the effect of changing the popularity fading parameter β and the rescaled temperature $T \cdot (d-1)$, with the number of dimensions d and the half m of the expected average degree $\bar{k}$ given in the title of the subplot pair. The number of nodes was $N = 10,000$ in each network. The curvature K of the hyperbolic space was always set to -1, i.e. we used $\zeta = 1$.

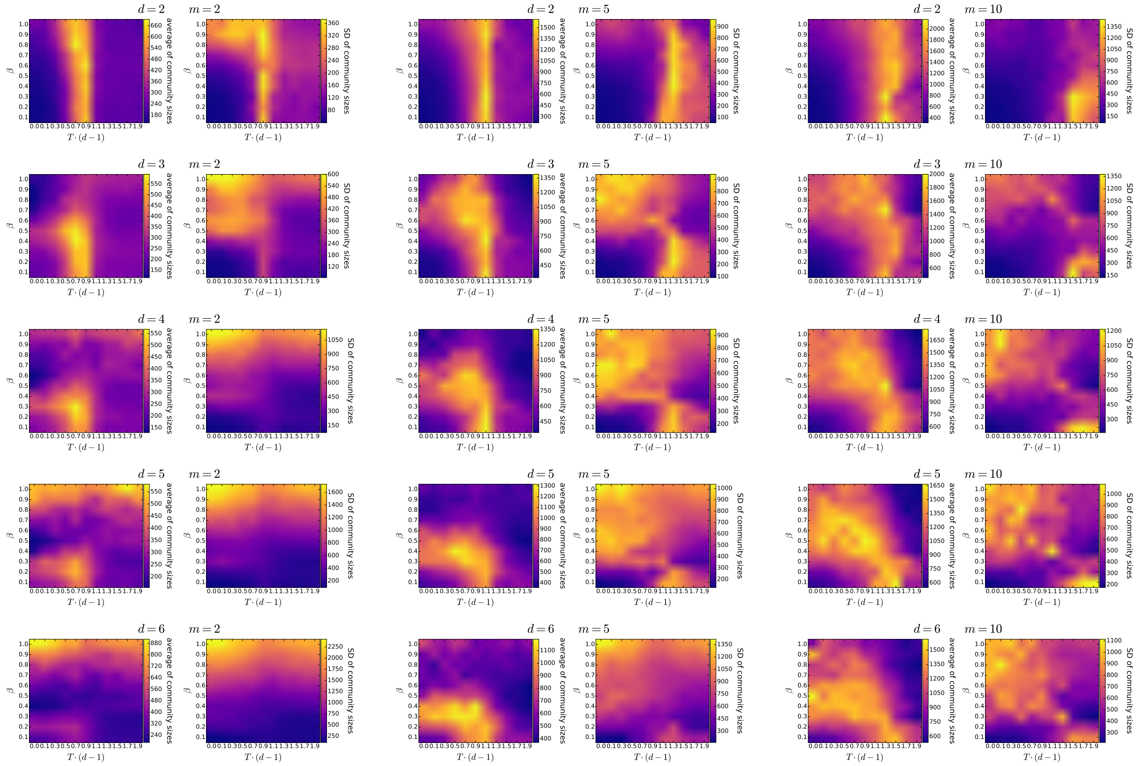

Figure S3.4. The mean and the standard deviation of the size of the communities detected by the *Louvain* algorithm in 5 dPSO networks in the case of different parametrisations. Each pair of subplots depicts the effect of changing the popularity fading parameter β and the rescaled temperature $T \cdot (d-1)$, with the number of dimensions d and the half m of the expected average degree $\bar{k}$ given in the title of the subplot pair. The number of nodes was $N = 10,000$ in each network. The curvature K of the hyperbolic plane was always set to -1, i.e. we used $\zeta = 1$.

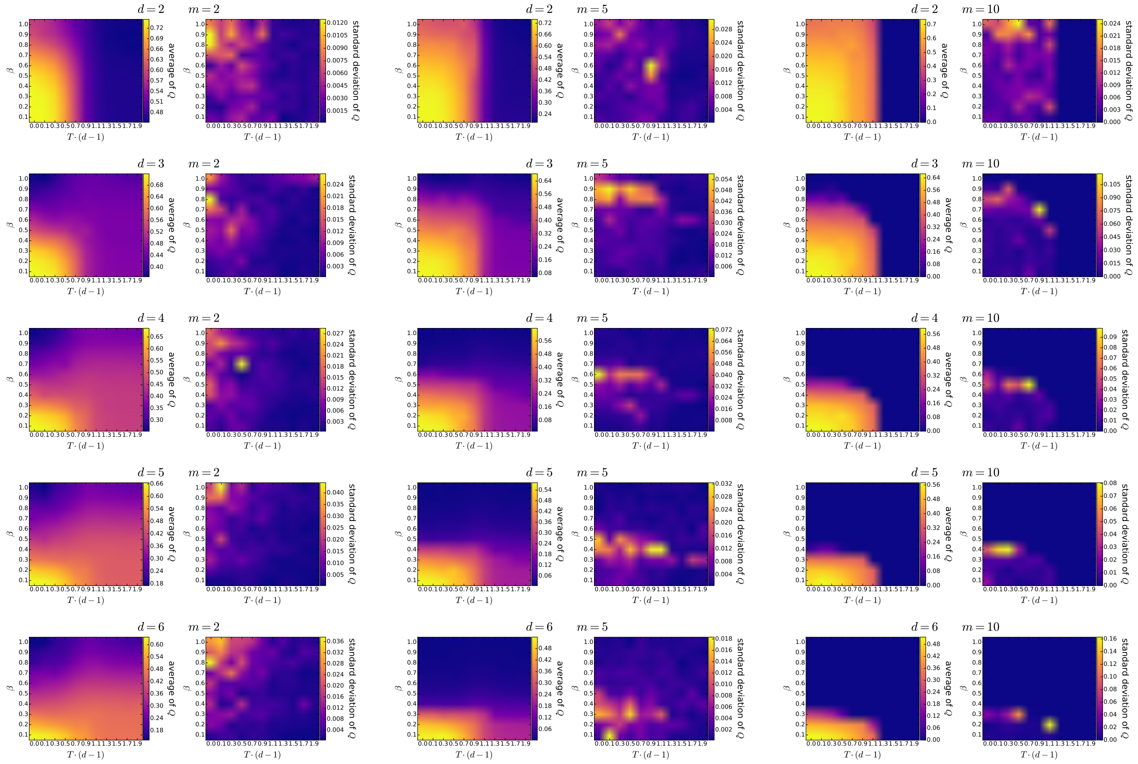

Figure S3.5. **The mean and the standard deviation of the modularity Q of the community structure detected by the *Infomap* algorithm in 5 dPSO networks in the case of different parametrisations.** Each pair of subplots depicts the effect of changing the popularity fading parameter β and the rescaled temperature $T \cdot (d-1)$, with the number of dimensions d and the half m of the expected average degree $\bar{k}$ given in the title of the subplot pair. The number of nodes was $N = 10,000$ in each network. The curvature K of the hyperbolic space was always set to -1, i.e. we used $\zeta = 1$.

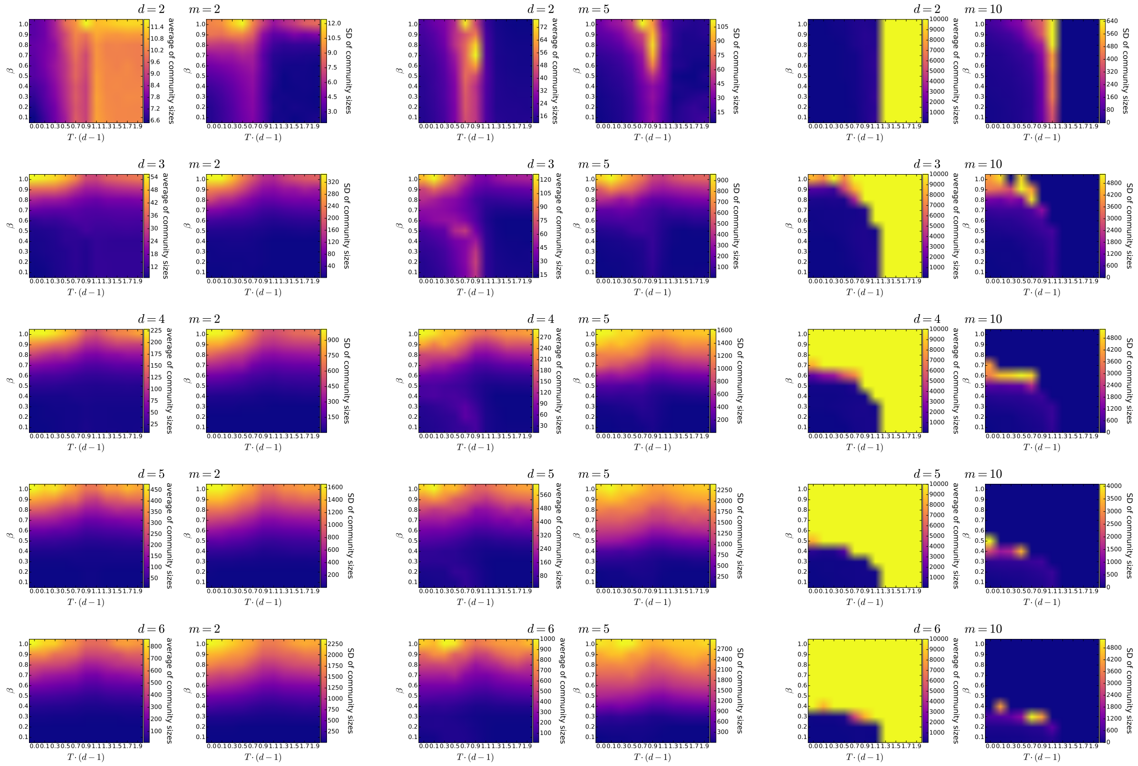

Figure S3.6. **The mean and the standard deviation of the size of the communities detected by the *Infomap* algorithm in 5 *d*PSO networks in the case of different parametrisations.** Each pair of subplots depicts the effect of changing the popularity fading parameter β and the rescaled temperature $T \cdot (d-1)$, with the number of dimensions d and the half m of the expected average degree $\bar{k}$ given in the title of the subplot pair. The number of nodes was $N = 10,000$ in each network. The curvature K of the hyperbolic plane was always set to -1, i.e. we used $\zeta = 1$.

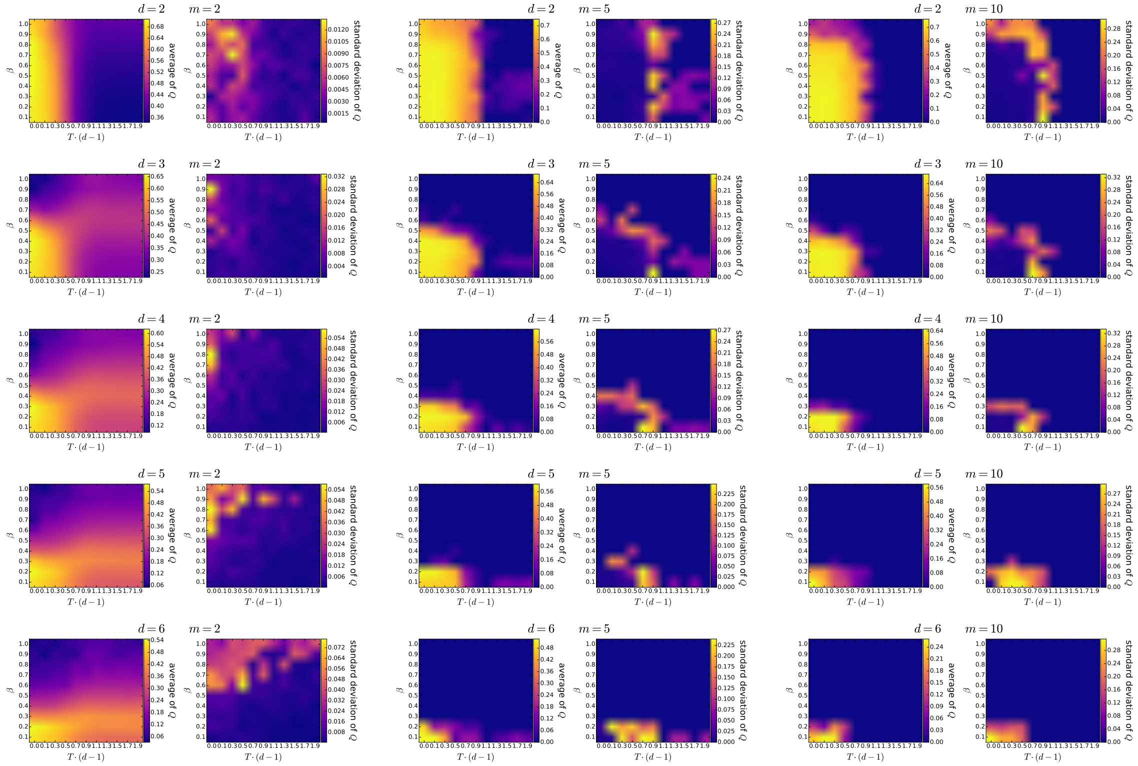

Figure S3.7. The mean and the standard deviation of the modularity Q of the community structure detected by the *asynchronous label propagation* algorithm in 5 dPSO networks in the case of different parametrisations. Each pair of subplots depicts the effect of changing the popularity fading parameter β and the rescaled temperature $T\cdot(d-1)$, with the number of dimensions d and the half m of the expected average degree $\bar{k}$ given in the title of the subplot pair. The number of nodes was $N = 10,000$ in each network. The curvature K of the hyperbolic space was always set to -1, i.e. we used $\zeta = 1$.

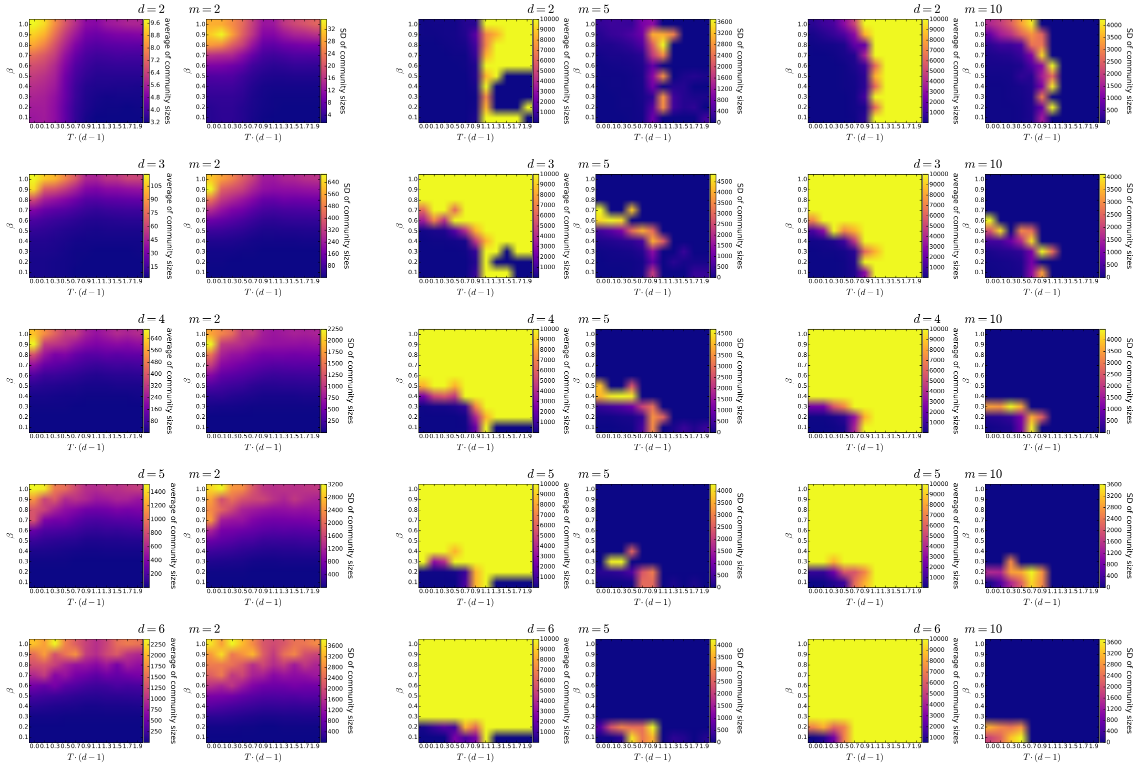

Figure S3.8. The mean and the standard deviation of the size of the communities detected by the *asynchronous label propagation* algorithm in 5 *d*PSO networks in the case of different parametrisations. Each pair of subplots depicts the effect of changing the popularity fading parameter β and the rescaled temperature $T \cdot (d-1)$, with the number of dimensions d and the half m of the expected average degree $\bar{k}$ given in the title of the subplot pair. The number of nodes was $N = 10,000$ in each network. The curvature K of the hyperbolic plane was always set to -1, i.e. we used $\zeta = 1$.

We also repeated our community analysis with a slight modification, taking into account the hyperbolic distances along the links. For this, we adopted the practice suggested in Ref. [12] and assigned weights to the links calculated from the hyperbolic distances between adjacent nodes as

$$w_{ij} \equiv w_{ji} = \frac{1}{1+x_{ij}}. \tag{S3.1}$$

Then, we searched for the communities of the obtained weighted graphs with the Louvain, the Infomap and the asynchronous label propagation methods (where all algorithms allow link weights to be taken into account). As before, for characterising the strength of the detected community structures we used modularity. However, instead of its original version described in Sect. 3.3 of the main text, here we used an extended form defined for weighted networks [17], where the total number of links E is replaced by $M = \frac{1}{2} \cdot \sum_{i=1}^{N} \sum_{j=1}^{N} w_{ij}$ (with w_{ij} denoting the link weight between nodes i and j), and the node degrees k_i and k_j are replaced by the node strengths s_i and s_j, defined e.g. for node i as $s_i = \sum_{\ell=1}^{N} w_{i\ell}$, resulting in the formula

$$Q = \frac{1}{2M} \cdot \sum_{i=1}^{N} \sum_{j=1}^{N} \left[w_{ij} - \frac{s_i s_j}{2M} \right] \delta_{c_i,c_j}. \tag{S3.2}$$

Similarly to Figs. S3.3-S3.8 dealing with the results of the community detection on unweighted networks, we present the averages and the standard deviations of the obtained modularities and community sizes in Figs. S3.9-S3.14 for the weighted case.

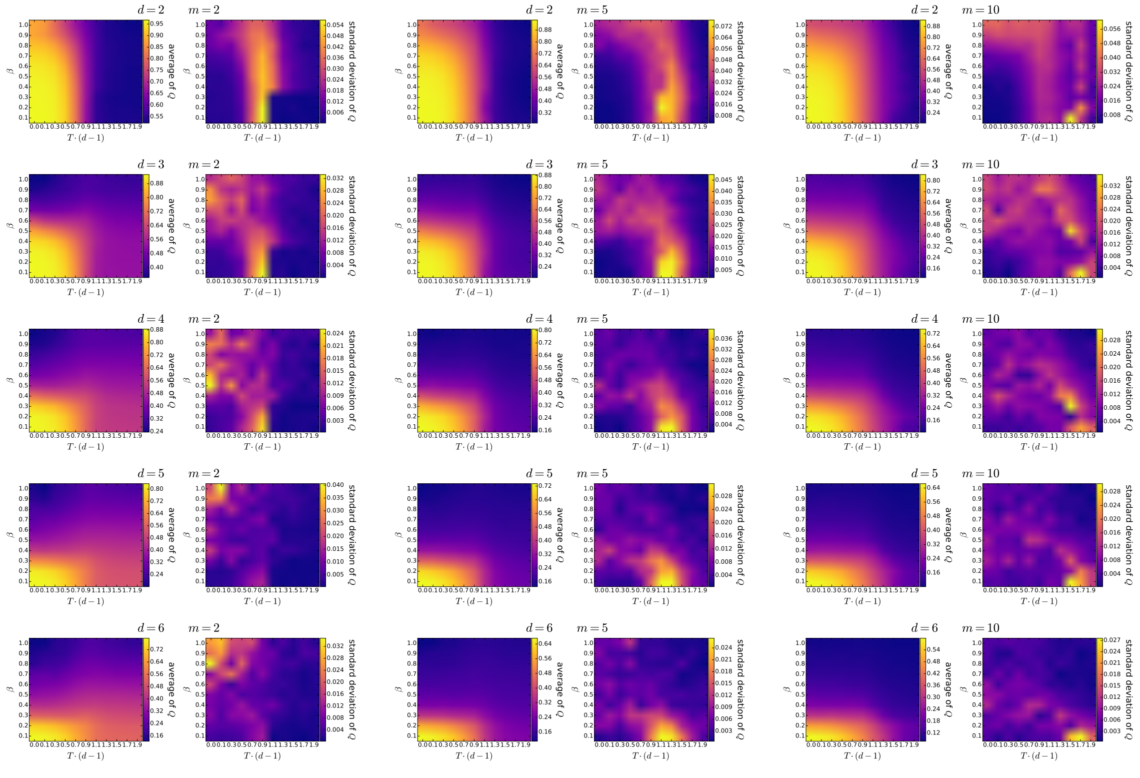

Figure S3.9. The mean and the standard deviation of the *weighted* modularity Q of the community structure detected by the *Louvain* algorithm in 5 dPSO networks in the case of different parametrisations, using link weights calculated from the hyperbolic distances between the connected nodes according to Eq. (S3.1). Each pair of subplots depicts the effect of changing the popularity fading parameter β and the rescaled temperature $T \cdot (d-1)$, with the number of dimensions d and the half m of the expected average degree $\bar{k}$ given in the title of the subplot pair. The number of nodes was $N = 10,000$ in each network. The curvature K of the hyperbolic space was always set to -1, i.e. we used $\zeta = 1$.

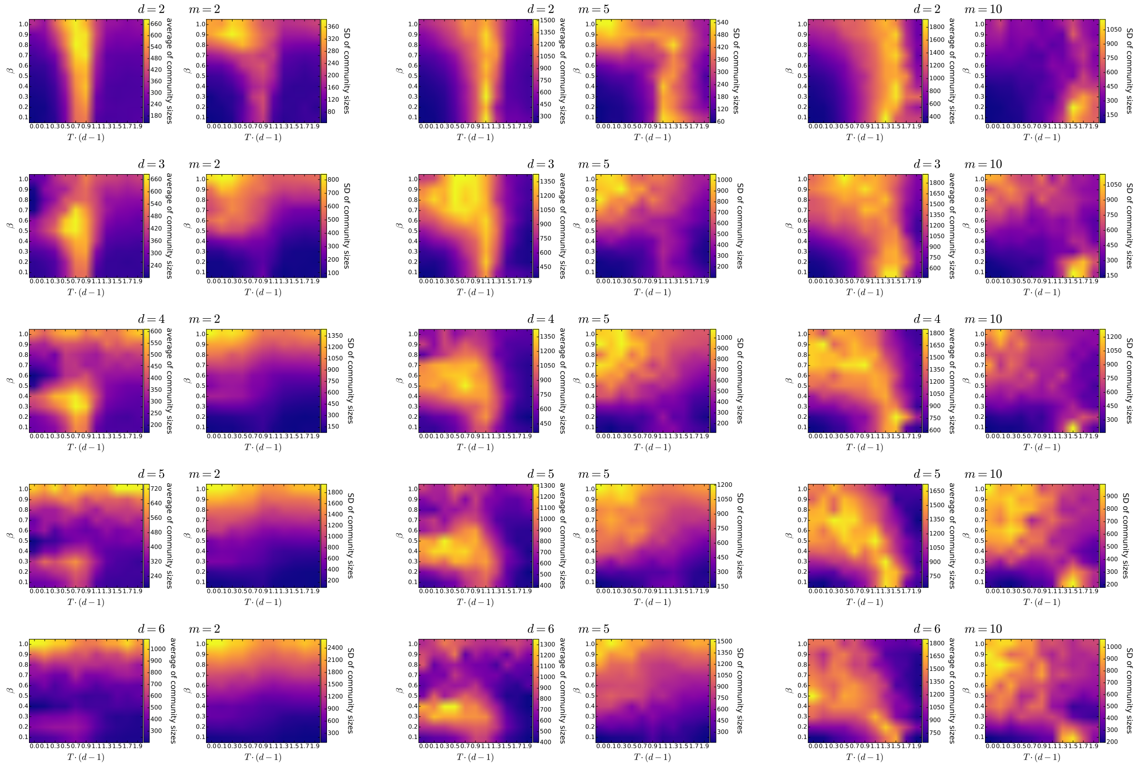

Figure S3.10. The mean and the standard deviation of the size of the communities detected by the *Louvain* algorithm in 5 *weighted d*PSO networks in the case of different parametrisations. Each pair of subplots depicts the effect of changing the popularity fading parameter β and the rescaled temperature $T \cdot (d-1)$, with the number of dimensions d and the half m of the expected average degree $\bar{k}$ given in the title of the subplot pair. The number of nodes was $N = 10,000$ in each network. The curvature K of the hyperbolic plane was always set to -1, i.e. we used $\zeta = 1$.

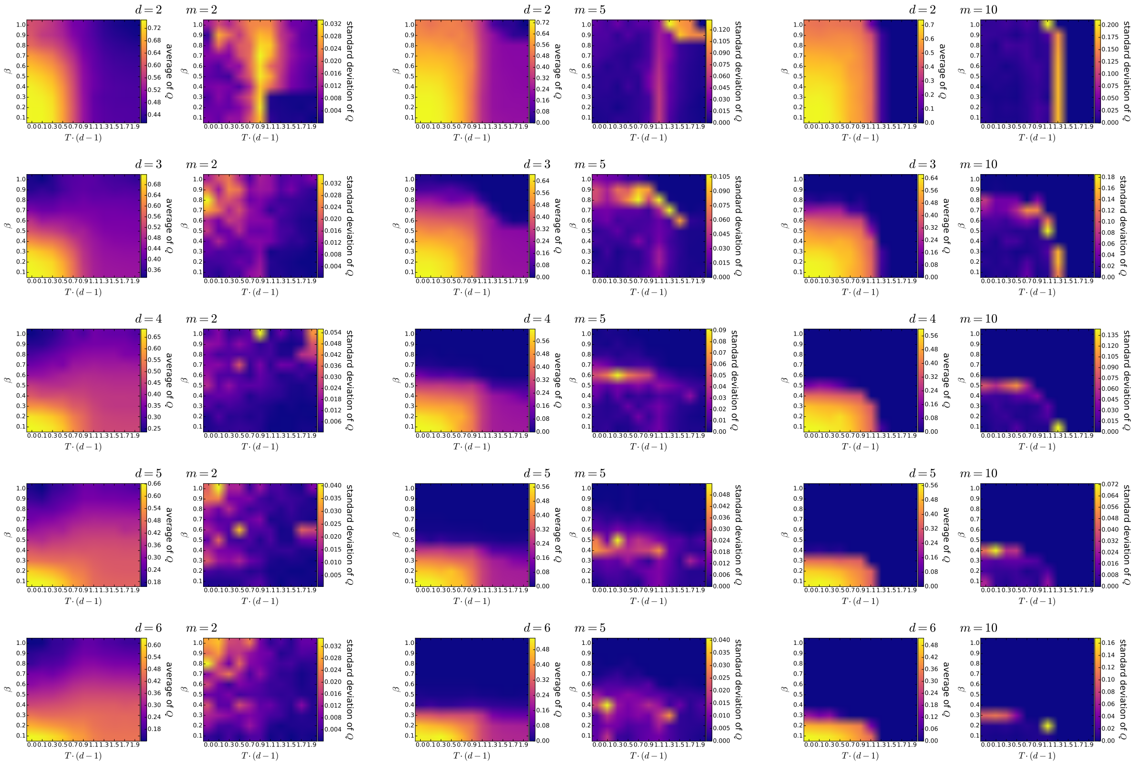

Figure S3.11. The mean and the standard deviation of the *weighted* modularity Q of the community structure detected by the *Infomap* algorithm in 5 dPSO networks in the case of different parametrisations, using link weights calculated from the hyperbolic distances between the connected nodes according to Eq. (S3.1). Each pair of subplots depicts the effect of changing the popularity fading parameter β and the rescaled temperature $T \cdot (d-1)$, with the number of dimensions d and the half m of the expected average degree $\bar{k}$ given in the title of the subplot pair. The number of nodes was $N = 10,000$ in each network. The curvature K of the hyperbolic space was always set to -1, i.e. we used $\zeta = 1$.

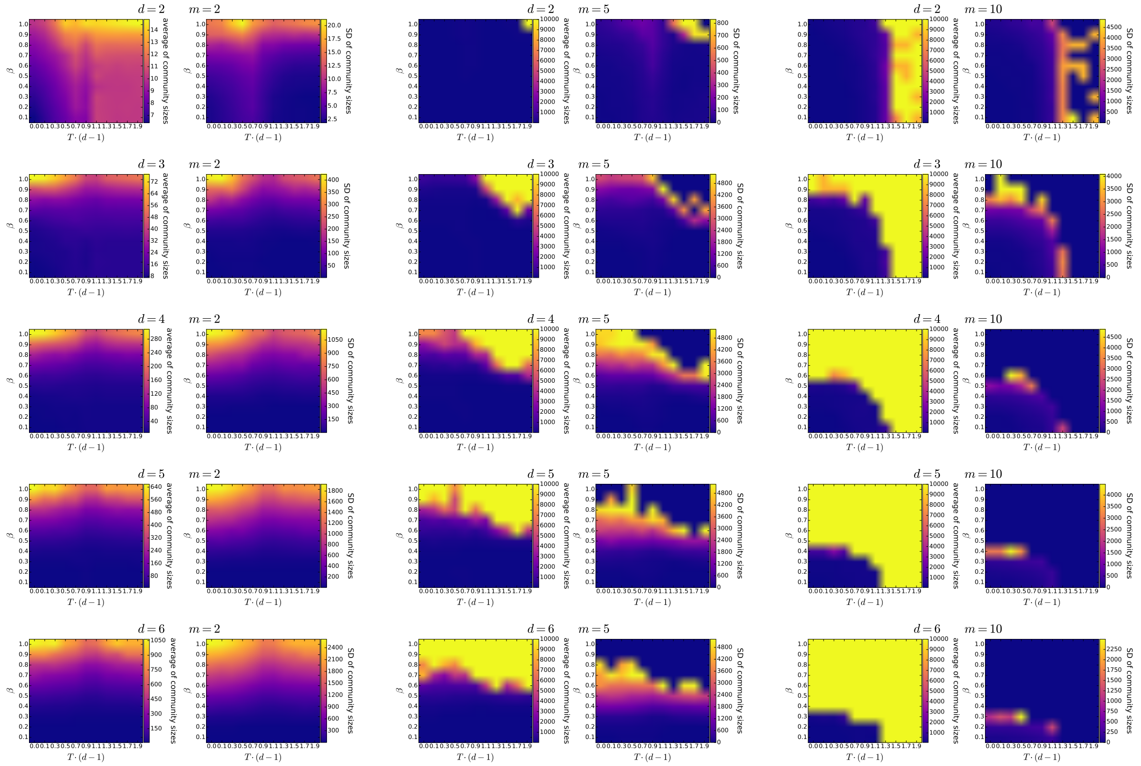

Figure S3.12. The mean and the standard deviation of the size of the communities detected by the *Infomap* algorithm in 5 *weighted d*PSO networks in the case of different parametrisations. Each pair of subplots depicts the effect of changing the popularity fading parameter β and the rescaled temperature $T \cdot (d-1)$, with the number of dimensions d and the half m of the expected average degree $\bar{k}$ given in the title of the subplot pair. The number of nodes was $N = 10,000$ in each network. The curvature K of the hyperbolic plane was always set to -1, i.e. we used $\zeta = 1$.

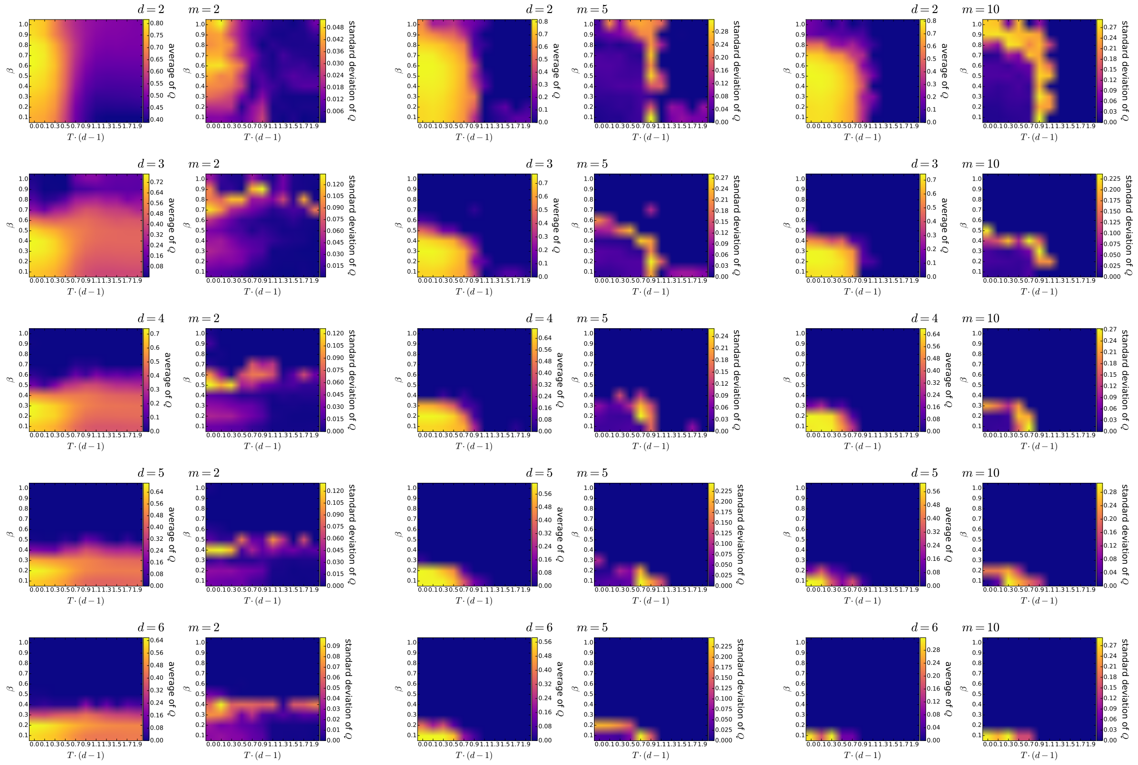

Figure S3.13. The mean and the standard deviation of the *weighted* modularity Q of the community structure detected by the *asynchronous label propagation* algorithm in 5 dPSO networks in the case of different parametrisations, using link weights calculated from the hyperbolic distances between the connected nodes according to Eq. (S3.1). Each pair of subplots depicts the effect of changing the popularity fading parameter β and the rescaled temperature $T \cdot (d-1)$, with the number of dimensions d and the half m of the expected average degree $\bar{k}$ given in the title of the subplot pair. The number of nodes was $N = 10,000$ in each network. The curvature K of the hyperbolic space was always set to -1, i.e. we used $\zeta = 1$.

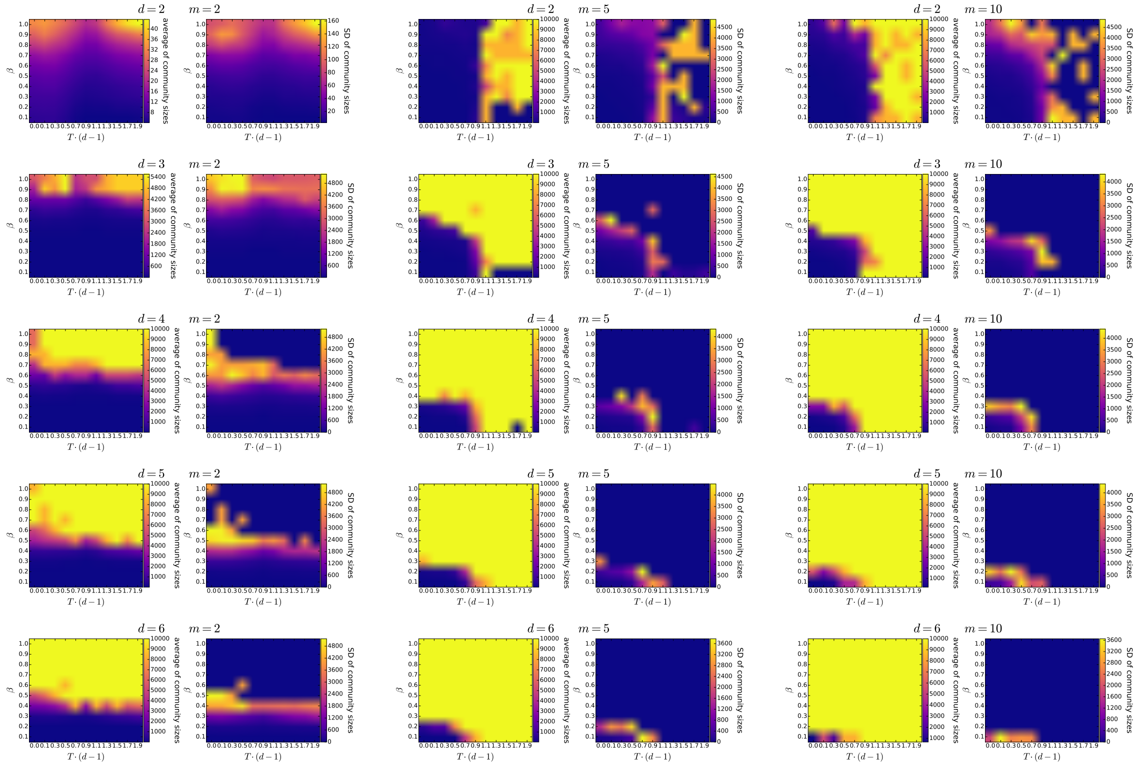

Figure S3.14. **The mean and the standard deviation of the size of the communities detected by the *asynchronous label propagation* algorithm in 5 *weighted d*PSO networks in the case of different parametrisations.** Each pair of subplots depicts the effect of changing the popularity fading parameter β and the rescaled temperature $T \cdot (d-1)$, with the number of dimensions d and the half m of the expected average degree $\bar{k}$ given in the title of the subplot pair. The number of nodes was $N = 10,000$ in each network. The curvature K of the hyperbolic plane was always set to -1, i.e. we used $\zeta = 1$.

Finally, we show some examples of the community size distributions obtained with the applied community finding methods for dPSO networks of different number of dimensions in Fig. S3.15. With regard to the shape of the curves, the same conclusion can be drawn for each dimension d as in Ref. [18] for $d = 2$, namely that Louvain yields relatively narrow, bell-shaped community size distributions concentrated at higher community sizes, while the community size distributions provided by asynchronous label propagation and Infomap are rather skewed, following more or less a power law in the former case and decaying somewhat faster towards the larger sizes in the latter case. As indicated by the slight right shift of the community size distributions in Fig. S3.15, when the dimension d of the hyperbolic space is increased while keeping the curvature K, the number of nodes N, the expected average degree $2m$, the degree decay exponent γ and the temperature T unaltered, all the examined community detection methods tend to find larger modules in the networks.

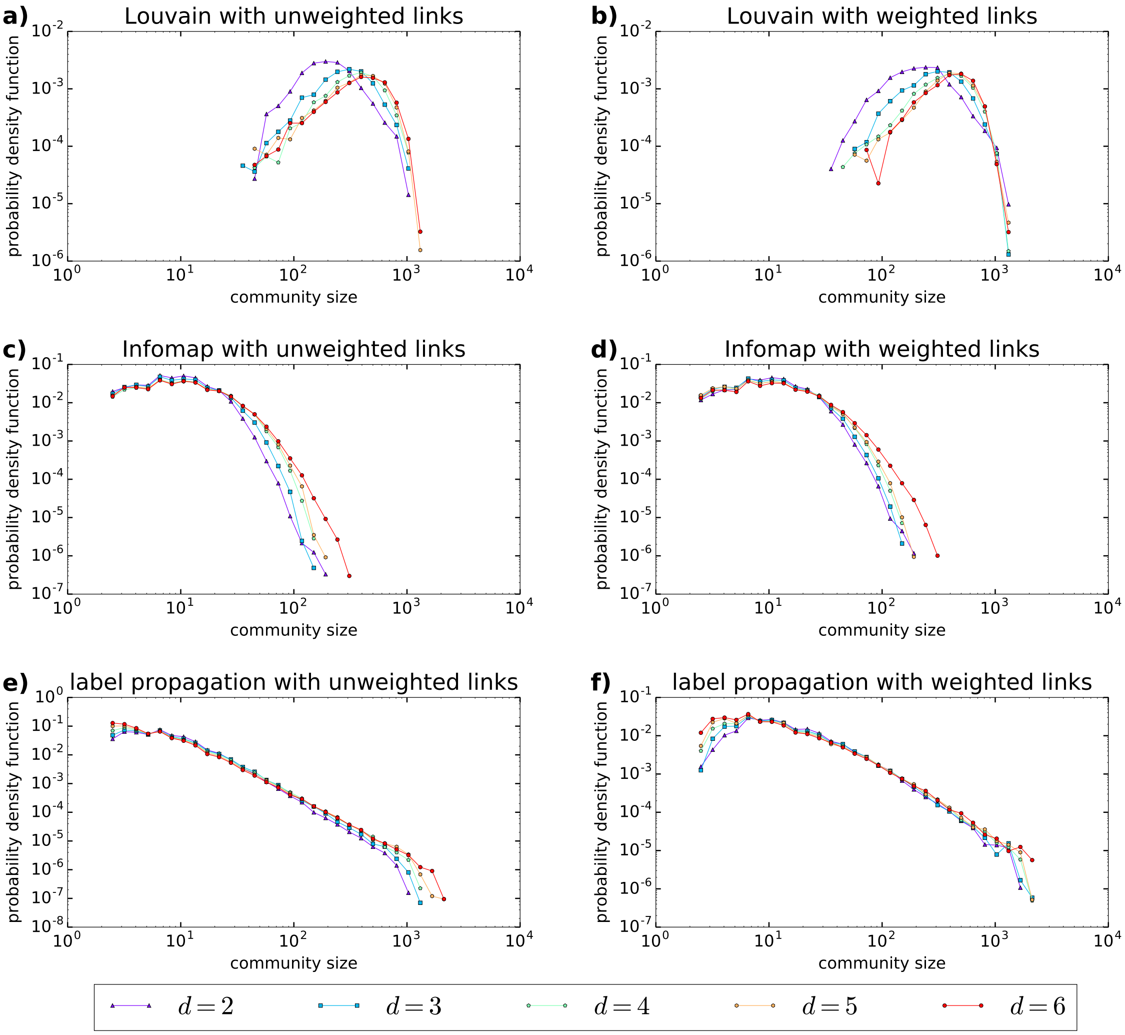

Figure S3.15. The size distribution of the communities detected by different community detection algorithms in 100 dPSO networks in the case of different values of the dimension d. Each panel shows the results of a community detection method specified in the panel title. The panels in the left column were obtained setting all the link weights in the networks to 1, whereas the panels in the right column were created using link weights calculated from the hyperbolic distances between the connected nodes according to Eq. (S3.1). The different colours of the curves correspond to the different values of the dimension d, listed in the legend. The curvature of the hyperbolic plane K was always set to -1, i.e. we used $\zeta = 1$. The number of nodes N was 10,000, the expected average degree $\bar{k} = 2m$ was 10, the temperature T was 0 and the degree decay exponent γ was set to 2.5 by using the popularity fading parameter $\beta = \frac{1}{(d-1)\cdot(2.5-1)}$ in each case.

S4 Nonuniform angular distribution

We have shown for the two-dimensional popularity-similarity optimisation model [1] in Ref. [18] and then for higher-dimensional cases in the present article that despite the absence of any intentional community formation mechanisms built into the model construction, dPSO networks possess an inherent, relevant community structure for a wide range of parameter settings. However, the dPSO model does not allow control over the number and the size of the communities. To deal with this problem, one can introduce heterogeneity in the angular node arrangement and generate networks using a nonuniform angular distribution of the network nodes, where denser angular regions can serve as built-in communities. This nonuniform popularity-similarity optimisation (nPSO) model has been studied in details in the two-dimensional case in Refs. [19, 20]. Here, as an example, we examine a simple three-dimensional case, where the N number of network nodes are distributed in equal proportions among C number of planted (angular) groups that are created by setting the angular node distribution to a mixture of C number of von Mises–Fisher distributions [21] of the same concentration parameter κ, with their mean directions $\underline{\mu}_i$ $(i = 1,2,...,C)$ distributed equidistantly over the unit 2-sphere. That is, we determined the angular coordinates of each network node by first choosing randomly one of the C number of mean directions $\underline{\mu}_i$ (representing the central locations of the angular sectors) and then sampling [22] a 3-dimensional unit vector $\underline{y}$ from the corresponding von Mises–Fisher distribution for which the probability density function is given by Eq. (S4.1). Note that a higher value of the concentration parameter κ means a higher concentration of the distributions around their mean direction, i.e. less spread and overlapping angular sectors, while $\kappa = 0$ pertains to the uniform angular distribution of the nodes. Our implementation of this three-dimensional nPSO model is available from Ref. [7].

$$f(\underline{y};\underline{\mu}_i,\kappa) = \frac{\kappa}{2\pi(e^{\kappa}-e^{-\kappa})} \cdot e^{\kappa \underline{\mu}_i^{\mathrm{T}} \underline{y}} \tag{S4.1}$$

The degree of separation among the planted groups is primarily determined by the concentration parameter κ and the temperature T, on the level of the angular node arrangement and the links, respectively. The effect of these two model parameters is demonstrated via some layouts in Fig. S4.1. Although e.g. $\kappa = 20$ already yields a noticeable separation among the patches of the network nodes, when T is increased to $1.5 \cdot T_{\mathrm{c}}$, then so many interconnections emerge between the patches that the planted modules eventually do not form actual communities. On the other hand, if the temperature is set to 0, the links become so localised that the planted groups split according to narrower angular regions. Nevertheless, for high enough values of the concentration parameter κ and moderate temperatures T, the planted groups were successfully identified by the community detection algorithm Louvain [10, 11] based on the edge list, without inputting any information about the network geometry.

In Fig. S4.2, we show how the average clustering coefficient $\bar{c}$, the adjusted mutual information AMI [23, 24] of the planted community structure and the one detected by the Louvain algorithm (setting all the link weights to 1), and the modularity [8, 9] of the planted and the detected network partitions depend on the temperature for different concentration parameters. Similarly to what has been shown in Fig. 3 of the main text for the uniform dPSO model, the average clustering coefficient $\bar{c}$ measured in three-dimensional nPSO networks gradually decreases with the increase in the temperature T before settling to a more or less constant value just above the critical temperature $T_{\mathrm{c}} = 1/(d-1) = 0.5$. One can also observe that if κ is higher, i.e. the angular patches of the network nodes are more separated from each other, then the drop in $\bar{c}$ occurring when switching from the deterministic connection rule ($T = 0$) to the probabilistic one ($0 < T$) is larger.

As expected, the adjusted mutual information of the planted and the detected partitions is 0 for $\kappa = 0$, when the angular position of the nodes is sampled from a mixture of uniform distributions and the nodes are assigned to the planted groups randomly, regardless of their angular coordinates. As we increase κ and create hereby larger angular gaps between the regions occupied by the network nodes, the AMI tends to become higher. At small temperatures, as exemplified by Fig. S4.1, the links are strongly localised, i.e. crowded into narrower sectors within the region of each planted group. Similarly to how the nodes of the whole hyperbolic space become grouped according to different angular regions in the case of the uniform PSO model [18], this angular confinement of the links splits the planted modules into smaller parts, yielding weaker agreement between the planted and the detected partitions. As the temperature begins to increase, at first the boundaries within the planted groups blur, and thus the AMI increases. Note that at large enough κ and moderate values of T, even AMI $= 1$ was achieved, meaning that the planted and the detected partitions were identical. However, even higher temperatures and farther-reaching connections already raise the interconnectedness of the adjacent planted groups too, until eventually the nodes lose their preference for primarily connecting to the members of their own planted group. Obviously, the larger the gaps between the occupied patches, the higher temperature is needed to enable the neighbouring patches to reach each other; thus, as κ increases, the AMI decreases more slowly to 0 as a function of T.

The behaviour of the modularities Q_{planted} and Q_{detected} as a function of the temperature T agrees with the results shown in Fig. 5 of the main text for the uniform dPSO model: according to this measure, both the planted and the detected community structure of three-dimensional nPSO networks lose from their strength as the temperature increases, and eventually both

modularities settle to a constant value. It is important to note that the modularity of the planted network partitions practically does not exceed the modularity of the detected community structures, meaning that whenever we measure low modularity values, these arise due to the network structure and not because of some failure in the applied community finding algorithm. Since the presence of wider angular gaps between the patches of the nodes decreases the probability of interconnections, larger values of κ lead to higher modularities. Nevertheless, at high enough temperatures the probability for the emergence of connections can become non-negligible even for the members of angularly well-separated patches, diminishing the modularity differences among the networks characterised by different concentration parameters.

According to Fig. S4.2, while the maximum point of both the average clustering coefficient $\bar{c}$ and the modularities Q_{planted} and Q_{detected} is at $T = 0$, with respect to the adjusted mutual information of the planted and the detected partitions it is better to choose higher temperatures. Nonetheless, Fig. S4.3 demonstrates that this is not a general rule, and with proper parameter settings one can generate networks that are simultaneously highly clustered and possess a strong planted community structure that is also well detectable.

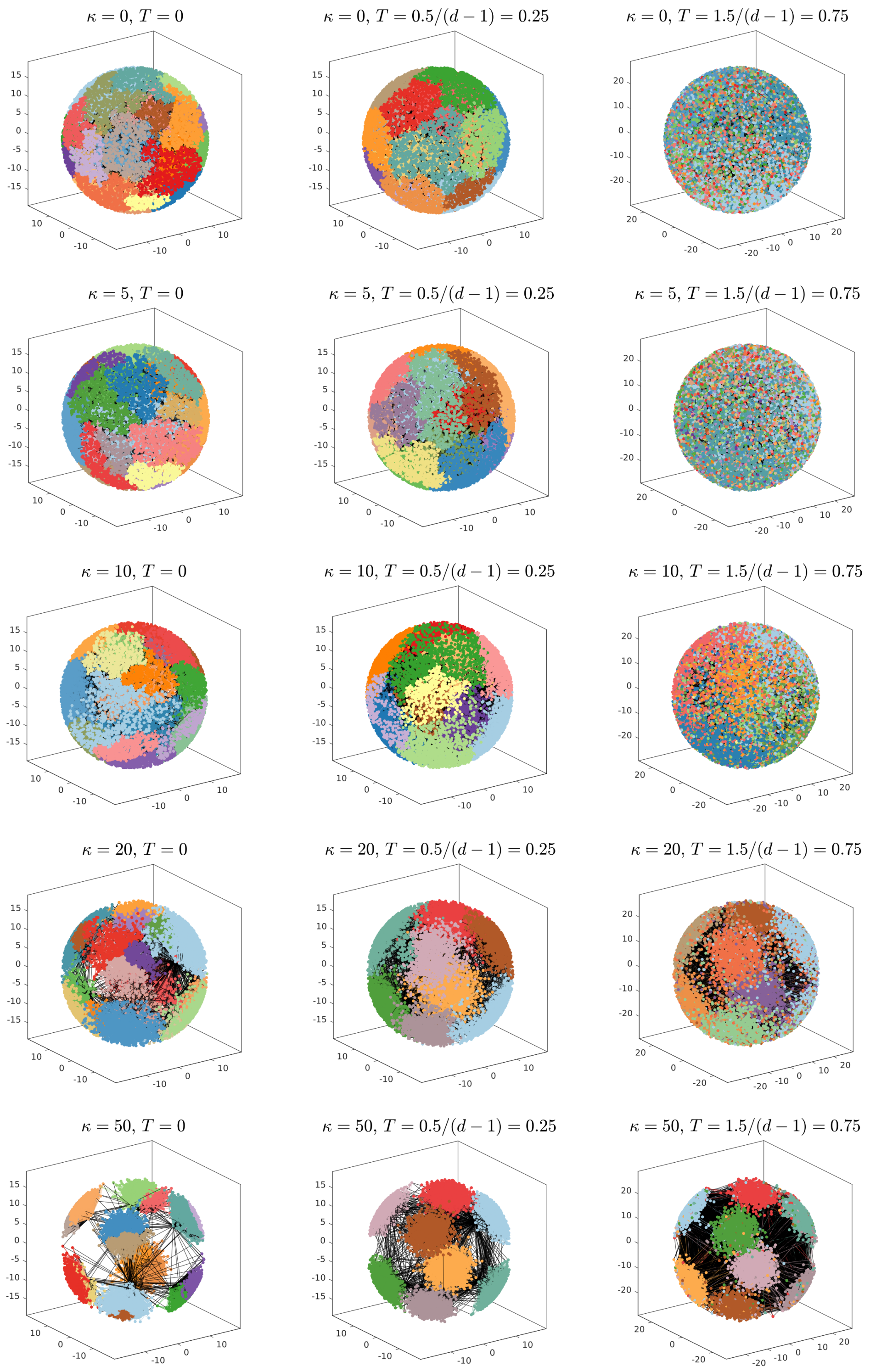

Figure S4.1. Layout of three-dimensional nPSO networks in the native representation of the hyperbolic space of curvature $K = -1$ at different values of the concentration parameter κ and the temperature T. Each row of panels was created using a given concentration parameter κ, and each column of subplots presents the results obtained with a given value of the temperature T, as written in the panel titles. Each network was generated setting the number N of nodes to 10,000, the half m of the expected average degree $\bar{k}$ to 5, the popularity fading parameter β to 1/3 (yielding the degree decay exponent $\gamma = 2.5$) and the number C of components of the mixture distribution describing the angular arrangement of the network nodes to 8. Note that the critical temperature was $T_c = 1/(d-1) = 0.5$.

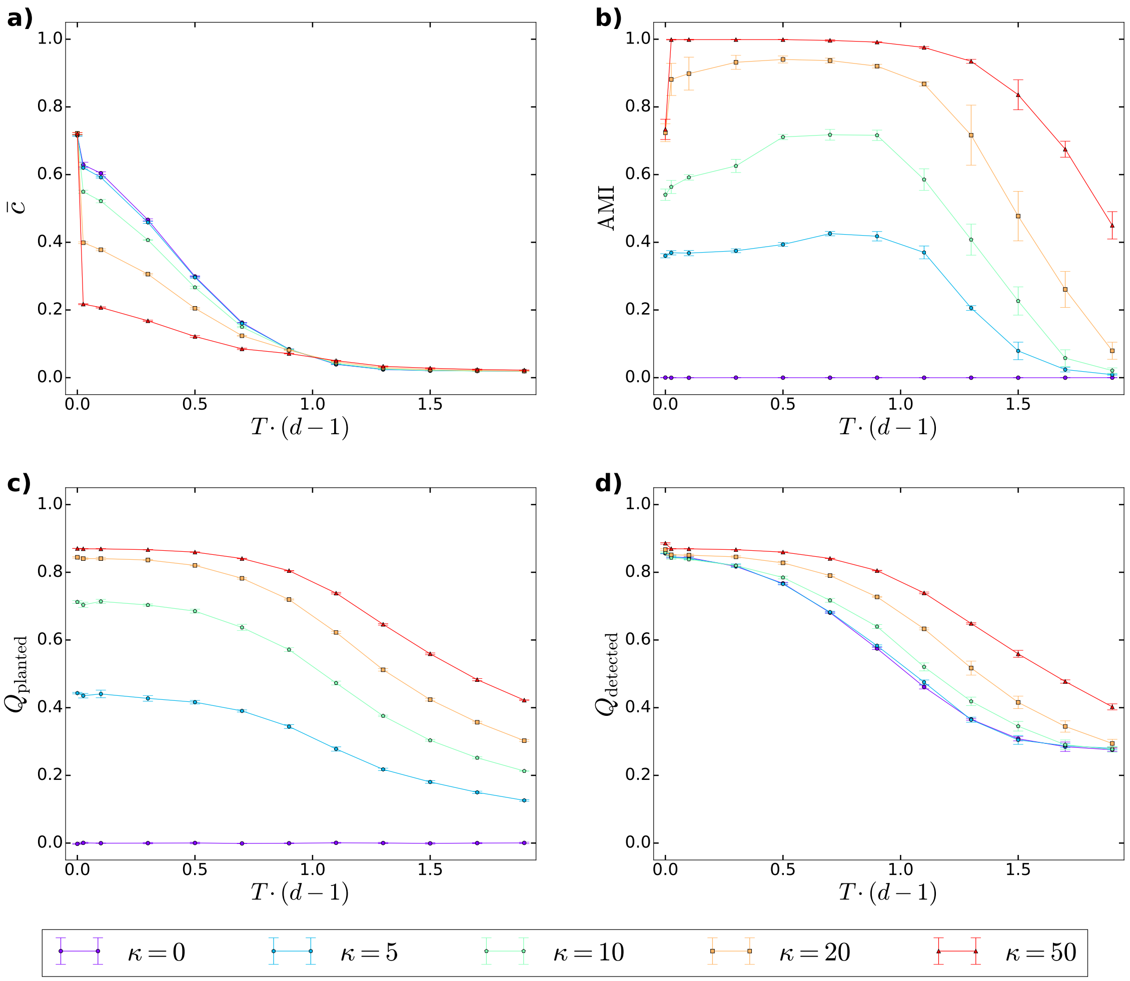

Figure S4.2. Average clustering coefficient $\bar{c}$, adjusted mutual information AMI of the planted modules and the ones detected by the Louvain algorithm and modularity Q of the planted and the detected network partitions as a function of the rescaled temperature $T \cdot (d-1)$ in 3-dimensional nPSO networks of different values of the concentration parameter κ. The data points show the measured values averaged over 5 networks of the given parameter setting, and the error bars indicate the standard deviations among the 5 networks. Each network was generated in the 3-dimensional hyperbolic space of curvature $K = -1$, setting the number N of nodes to 10,000, the half m of the expected average degree $\bar{k}$ to 5, the popularity fading parameter β to 1/3 (i.e., the degree decay exponent γ to 2.5), and the number C of components of the mixture distribution describing the angular arrangement of the network nodes to 8.

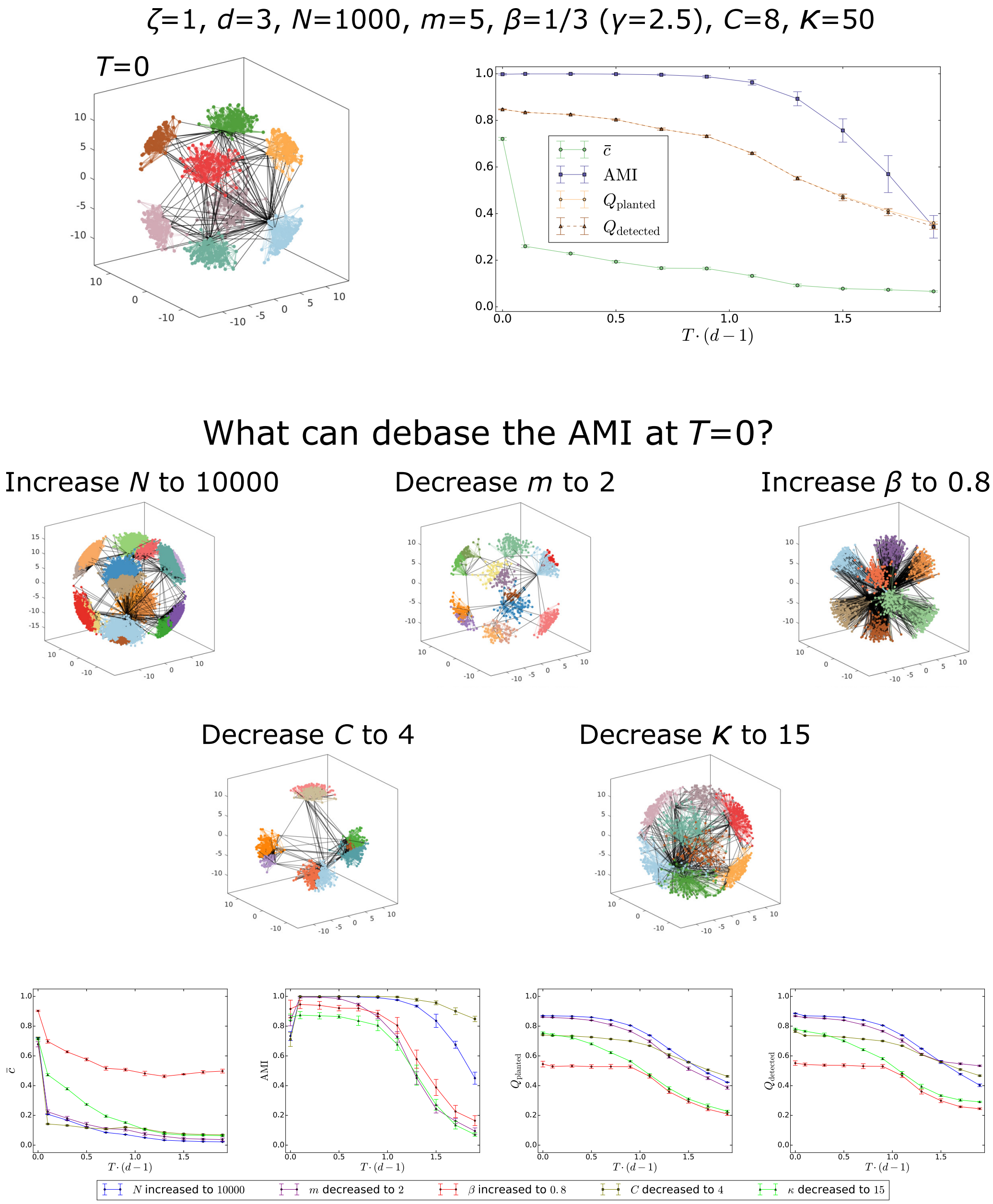

Figure S4.3. With proper settings of the parameters of the three-dimensional nPSO model, large adjusted mutual information of the planted and the detected modules can be achieved even at the temperature minimum $T = 0$, where the average clustering coefficient and the modularities are maximised for the given parametrisation. However, increasing the number N of nodes, decreasing the expected average degree $2 \cdot m$, increasing the popularity fading parameter β, decreasing the number C of components of the mixture distribution describing the angular arrangement of the network nodes, or decreasing the concentration parameter κ may reduce the AMI at $T = 0$ compared to the values obtained at slightly higher temperatures. In the right uppermost chart we show an example where – in contrast with Fig. S4.2 – the decay of the AMI towards the smallest possible temperatures does not appear. The layouts below exemplify how the change of the different model parameters compared to the settings of the uppermost figures can debase the high AMI value achieved at $T = 0$. The lowermost charts depict how the increase in the rescaled temperature $T \cdot (d-1)$ affects the average clustering coefficient $\bar{c}$, the adjusted mutual information AMI and the modularity Q of the planted partitions and the ones detected by Louvain in the five cases presented by the layouts above. The plotted data points correspond to the values averaged over 5 networks in the case of $N = 10,000$ and 10 networks for $N = 1000$. The error bars indicate the standard deviations among the networks of the same parameter setting.

Lastly, we demonstrate in Fig. S4.4 that when each network node is assigned randomly to one of the 8 planted groups corresponding to von Mises–Fisher distributions of the same concentration parameter κ and mean directions pointing toward the vertices of a cube, then the resulting degree distribution reasonably preserves its form of $\mathcal{P}(K=k) \sim k^{-\gamma}$ with $\gamma = 1 + \frac{1}{(d-1)\cdot\beta}$, even if the angular distribution of the nodes becomes more and more heterogeneous due to the increasing separation of the occupied angular regions obtained at higher and higher values of κ. Similarly to what has been shown in Fig. 2 of the main text concerning the uniform dPSO model, the temperature T does not have a significant effect on the degree distribution even for the nonuniform three-dimensional PSO model.

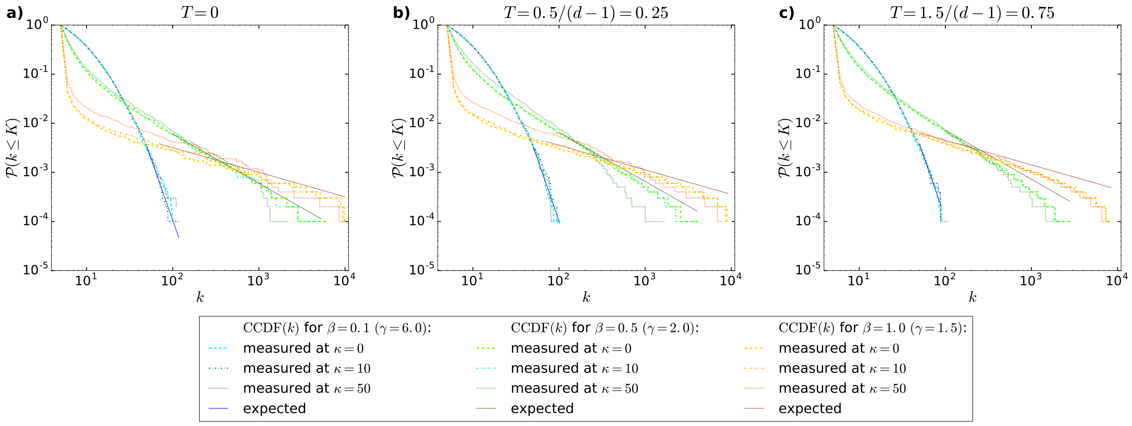

Figure S4.4. Degree distribution of networks generated by the three-dimensional nPSO model using different values of the temperature T, the popularity fading parameter β and the concentration parameter κ. As expected, the tail of the complementary cumulative distribution function (CCDF) of the node degrees follows a power-law that can be written in the form of $\mathcal{P}(k \leq K) \sim k^{-(\gamma-1)}$ with $\gamma = 1 + \frac{1}{(d-1)\cdot\beta}$, independently of the choice of κ and T. One network was generated with all the parameter settings. The curvature of the three-dimensional hyperbolic space, the number of nodes, the half of the expected average degree and the number of components of the mixture distribution describing the angular arrangement of the network nodes were the same for each network, namely $K = -\zeta^2 = -1$, $N = 10,000$, $m = 5$ and $C = 8$. Panel a) corresponds to the case of the deterministic connection rule ($T = 0$), panel b) shows the curves obtained at the half of the critical temperature $T_c = 1/(d-1)$, while panel c) presents the results at a higher temperature of $1.5 \cdot T_c$. The CCDF curves obtained with the different values of the concentration parameter κ are well grouped according to the popularity fading parameters, i.e. the expected degree decay exponents in all panels.

References

1. Papadopoulos, F., Kitsak, M., Serrano, M. Á., Boguñá, M. & Krioukov, D. Popularity versus similarity in growing networks. *Nature* **489**, 537 EP – (2012).
2. Dorogovtsev, S. N., Mendes, J. F. F. & Samukhin, A. N. Structure of growing networks with preferential linking. *Phys. Rev. Lett.* **85**, 4633–4636, DOI: 10.1103/PhysRevLett.85.4633 (2000).
3. Krioukov, D., Papadopoulos, F., Kitsak, M., Vahdat, A. & Boguñá, M. Hyperbolic geometry of complex networks. *Phys. Rev. E* **82**, 036106, DOI: 10.1103/PhysRevE.82.036106 (2010).
4. Zuev, K., Boguñá, M., Bianconi, G. & Krioukov, D. Emergence of soft communities from geometric preferential attachment. *Sci. Rep.* **5**, 9421, DOI: doi:10.1038/srep09421 (2015).
5. García-Pérez, G., Allard, A., Serrano, M. Á. & Boguñá, M. Mercator: uncovering faithful hyperbolic embeddings of complex networks. *New J. Phys.* **21**, 123033, DOI: 10.1088/1367-2630/ab57d2 (2019).
6. Kitsak, M., Aldecoa, R., Zuev, K. & Krioukov, D. Random hyperbolic graphs in $d+1$ dimensions (2020). Preprint at arXiv:2010.12303 [physics.soc-ph].
7. The code will be available at https://github.com/BianKov/dPSO upon publication.
8. Newman, M. E. J. & Girvan, M. Finding and evaluating community structure in networks. *Phys. Rev. E* **69**, 026113 (2004).
9. We calculated the modularity values with the python function 'modularity' available in the 'networkx.algorithms.community.quality' package.
10. Blondel, V. D., Guillaume, J.-L., Lambiotte, R. & Lefebvre, E. Fast unfolding of communities in large networks. *J. Stat. Mech. Theory Exp.* **2008**, P10008, DOI: 10.1088/1742-5468/2008/10/p10008 (2008).
11. We used the python implementation of the louvain algorithm available at https://github.com/taynaud/python-louvain.
12. Muscoloni, A., Thomas, J. M., Ciucci, S., Bianconi, G. & Cannistraci, C. V. Machine learning meets complex networks via coalescent embedding in the hyperbolic space. *Nat. Commun.* **8**, 1615, DOI: 10.1038/s41467-017-01825-5 (2017).
13. Rosvall, M. & Bergstrom, C. T. Multilevel compression of random walks on networks reveals hierarchical organization in large integrated systems. *PLOS ONE* **6**, 1–10, DOI: 10.1371/journal.pone.0018209 (2011).
14. We used the python package for the infomap algorithm available at https://pypi.org/project/infomap/.
15. Raghavan, U. N., Albert, R. & Kumara, S. Near linear time algorithm to detect community structures in large-scale networks. *Phys. Rev. E* **76**, 036106, DOI: 10.1103/PhysRevE.76.036106 (2007).
16. We used the python function 'asyn_lpa_communities', an implementation of the asynchronous label propagation algorithm available in the 'networkx.algorithms.community.label_propagation' package.
17. Newman, M. E. J. Analysis of weighted networks. *Phys. Rev. E* **70**, 056131, DOI: 10.1103/PhysRevE.70.056131 (2004).
18. Kovács, B. & Palla, G. The inherent community structure of hyperbolic networks. *Sci. Reports* **11**, 16050, DOI: 10.1038/s41598-021-93921-2 (2021).
19. Muscoloni, A. & Cannistraci, C. V. A nonuniform popularity-similarity optimization (npso) model to efficiently generate realistic complex networks with communities. *New J. Phys.* **20**, 052002 (2018).
20. Muscoloni, A. & Cannistraci, C. V. Leveraging the nonuniform pso network model as a benchmark for performance evaluation in community detection and link prediction. *New J. Phys.* **20**, 063022 (2018).
21. Mardia, K. V. & Jupp, P. E. *Directional Statistics.* (John Wiley and Sons Ltd., 2000), 2nd edn.
22. For sampling positions from the von mises–fisher distribution, we used the python implementation available at https://www.tensorflow.org/probability/api_docs/python/tfp/distributions/vonmisesfisher.
23. Vinh, N. X., Epps, J. & Bailey, J. Information theoretic measures for clusterings comparison: Variants, properties, normalization and correction for chance. *J. Mach. Learn. Res.* **11**, 2837–2854 (2010).
24. We calculated the adjusted mutual information values with the python function 'adjusted_mutual_info_score' available in the 'sklearn.metrics.cluster' package.
